# Light Metal Pyrazolates Grafted onto Periodic Mesoporous Silica for Carbon Dioxide Capture and Transformation

**DOI:** 10.1021/acs.inorgchem.5c05578

**Published:** 2026-02-03

**Authors:** Felix Kracht, Jitpisut Poolwong, Natascha M. Roth, Yucang Liang, Cäcilia Maichle-Mössmer, Reiner Anwander

**Affiliations:** Institut für Anorganische Chemie, Eberhard Karls Universität Tübingen (EKUT), Auf der Morgenstelle 18, Tübingen 72076, Germany

## Abstract

Recent advances have shown that light metal pyrazolate complexes not only achieve high CO_2_ uptake but are also able to convert epoxides and CO_2_ to cyclic carbonates catalytically. Surface organometallic chemistry (SOMC) combines reactive metal complexes with the durability and robustness of a support material to form environmentally even more benign materials for CO_2_ capture and conversion. In this study, light metal pyrazolates with a variety of oxidation states and ionic radii were grafted onto mesoporous silica SBA-15_500_ affording the hybrid materials [Mg­(pz^*t*Bu2^)_2_]_2_@SBA-15_500_, Al­(pz^*t*Bu2^)_3_@SBA-15_500_, Ti^+IV^(pz^Me2^)_4_@SBA-15_500_, and Ti^+III^(pz^*t*Bu2^)_3_@SBA-15_500_. The hybrid materials were characterized via N_2_ physisorption, elemental analysis, ICP/OES, DRIFTS, and solid-state NMR spectroscopy, suggesting successful grafting with monometallic surface species and revealing a CO_2_ uptake of up to 11 wt%. In addition, Ti^+IV^(pz^Me2^)­[OSi­(O*t*Bu)_3_]_3_ was synthesized as a model complex for surface species likely present for Ti^+IV^(pz^Me2^)_4_@SBA-15_500_. Complex Ti^+IV^(pz^Me2^)­[OSi­(O*t*Bu)_3_]_3_ is also able to insert CO_2_ under the formation of the carbamate complex Ti^+IV^(CO_2_·pz^Me2^)­[OSi­(O*t*Bu)_3_]_3_, emulating material CO_2_@Ti^+IV^(pz^Me2^)_4_@SBA-15_500_. All hybrid materials under study are active catalysts in the cycloaddition of epoxides with CO_2_ to form cyclic carbonates. The magnesium hybrid material [Mg­(pz^*t*Bu2^)_2_]_2_@SBA-15_500_ exceeds its homogeneous congener, featuring high conversion even for bulkier epoxides along with a desirable reusability.

## Graphical abstract

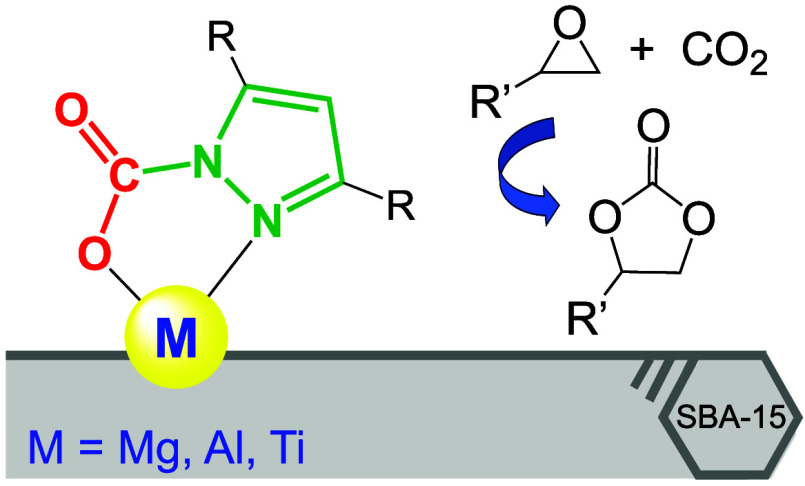

## Introduction

Combatting the steady rise of anthropogenic carbon dioxide emissions and associated global climate change is one of the most pressing concerns of our times, particularly in the environmental and chemical sectors.^1^ Two prominent approaches to thwart the rising CO_2_ level are carbon capture and storage, as well as using CO_2_ as C1 feedstock for synthesizing more valuable organic compounds.^2–11^ Carbon capture can be effective by chemisorption in both the liquid and solid phases. In industry, aqueous amine scrubbers or alkali/alkaline-earth-metal hydroxide/carbonate solutions (caustizication processes) are used as CO_2_ capture media; however, they display low capacities and require high regeneration energies.^12–14^ Capture systems that are currently under research include amino-functionalized materials such as porous silica, zeolites, and metal–organic frameworks (MOF), as well as ionic liquids.^5,7–9,15–17^

Surface organometallic chemistry (SOMC) is a promising material approach for targeting the catalytic conversion of CO_2_.^18,19^ By definition, metal–organic complexes are grafted onto a surface-rich support like periodic mesoporous silica (PMS) to generate well-defined surface species of enhanced/distinct reactivity.^20,21^ Moreover, surface confinement and site isolation counteract (intermolecular) deactivation processes and promote increased stability under ambient conditions. Several SOMC-derived heterogeneous catalysts have been recently reported to promote the cycloaddition of epoxides and CO_2_.^22–25^ Although such heterogeneous catalysts can exhibit lower activity compared to that of their homogeneous derivatives, this apparent drawback is often compensated by higher stability and reusability.

Recently, we found that cerium pyrazolates like homoleptic ceric [Ce­(pz^Me2^)_4_]_2_ insert CO_2_ exhaustively and reversibly under formation of the carbamate complex Ce­(CO_2_·pz^Me2^)_4_.^26^ This conceptional approach could be also applied to light metal pyrazolates of magnesium, aluminum and titanium, leading to the isolation of Mg­(CO_2_·pz^*t*Bu2^)_2_(thf)_2_, Al­(CO_2_·pz^*t*Bu2^)_2_(pz^*t*Bu2^) and Ti­(CO_2_·pz^Me2^)_2_(pz^Me2^)_2_.^27–29^ Reversible and excessive CO_2_ uptake was also found when cerium and lanthanum pyrazolates, [Ce­(pz^Me2^)_4_]_2_, [Ce­(pz^Me2^)_3_]_4_ and [Ln­(pz^Me2^)_3_(thf)]_2_ (Ln = La, Ce) were grafted onto mesoporous silica SBA-15_500_.^24^ High capacities of up to 20 wt% CO_2_ were found for [Ln­(pz^Me2^)_3_(thf)]_2_@SBA-15_500_. In addition, tetravalent [Ce­(pz^Me2^)_4_]_2_@SBA-15_500_ acts as an effective and easily recyclable heterogeneous catalyst. Naturally, we were interested in the synthesis of hybrid materials based on light metal pyrazolates and silica SBA-15 with large mesopores and their reactivity toward CO_2_. Compared to rare-earth metals, light metals should implicate higher weight percentages of inserted CO_2_.^27–29^ Moreover, the envisaged light metals magnesium, aluminum, and titanium are abundant, inexpensive, nontoxic, and thus favorably environmentally benign.

## Results and Discussion

### Parent PMS Material

SBA-15 silica seemed an appropriate choice of the PMS support because of its large mesopores facilitating intrapore chemistry. Periodic, long-range ordered silicas with a smaller pore size such as MCM-48 or MCM-41 are more likely to incur pore blockage when the relatively large pyrazolate complexes are immobilized on the surface. The synthesis of SBA-15 was conducted as described in the literature.^31^ The calcined material was activated at 500 °C and 10^–3^ mbar (denoted as SBA-15_500_). The synthesis, silylation, and full characterization of the parent SBA-15_500_ (including N_2_ physisorption analysis) were reported in a previous work.^25^ The surface Si-OH population was determined as 2.80 mmol/g of Si-OH groups.^30^ The silanol groups are clearly visible in the DRIFT spectrum by a sharp signal at ṽ = 3746 cm^–1^. N_2_ physisorption analysis revealed a surface area of 848 m^2^/g, a pore volume of 1.11 cm^3^/g, and a pore diameter of 8.2 nm (Table 1).

**Table 1:** Analytical Data of Parent and Hybrid Materials

material	*c*_SiOH surface_ ^a^ [mmol/g]	*a*_BET_ ^b^ [m^2^/g]	*V*_pore_ ^c^ [cm^3^/g]	*d*_pore_ ^d^ [nm]	*M* ^e^ [wt%]	N/M ratio^f^	M/SiOH ratio	CO_2_ uptake^g^ [wt%]
SBA-15_500_ ^h^	2.80	848	1.11	8.2				
**H1-Mg**		410	0.57	6.2	1.85	3.43	0.28	7
**H2-Al**		418	0.55	6.4	1.21	5.67	0.16	6
**H3-Ti^+IV^**		405	0.53	6.4	4.39	5.40	0.32	11
**H4-Ti^+III^**		397	0.55	6.5	2.66	4.63	0.20	

a Calculated according to published procedures using HN­(SiHMe_2_)_2_-promoted surface silylation.^30^

b Brunauer–Emmett–Teller (BET) surface area calcd. between *p*/*p*_0_ 0.07 and 0.15.

c Barrett–Joyner–Halenda (BJH) adsorption cumulative pore volume between 2.0 and 12 nm.

d Maximum pore size distribution calcd. by the BJH method from the isotherm adsorption branch.

e Metal content determined by inductively coupled plasma optical emission spectrometry (ICP-OES).

f Metal/nitrogen ratio determined via elemental analysis and ICP-OES.

g Uptake determined by weight increase of the sample after exposure to an excess of CO_2_;

h Characterized in a previous work.^25^

### Synthesis and Characterization of Light Metal (M) Pyrazolate Hybrid Materials H-M

To cover a variety of metals and oxidation states, the four pyrazolate complexes [Mg­(pz^*t*Bu2^)_2_]_2_, Al­(pz^*t*Bu2^)_3_, Ti^+IV^(pz^Me2^)_4_, and Ti^+III^(pz^*t*Bu2^)_3_ were chosen as metal–organic precursors (Scheme 1). The grafting reaction was performed by suspending the parent SBA-15 material in *n*-hexane (toluene for Ti^+IV^) and the addition of equimolar amounts of the metal–organic precursor dissolved in *n*-hexane (toluene for Ti^+IV^) (*c*(M)/*c*(OH) = 1). Such SOMC yielded the four hybrid materials [Mg­(pz^*t*Bu2^)_2_]_2_@SBA-15_500_ (**H1-Mg**), Al­(pz^*t*Bu2^)_3_@SBA-15_500_ (**H2-Al**), Ti^+IV^(pz^Me2^)_4_@SBA-15_500_ (**H3-Ti** ^**+IV**^) and Ti^+III^(pz^*t*Bu2^)_3_@SBA-15_500_ (**H4-Ti** ^**+III**^). In all cases, complete consumption of the Si-OH surface sites was confirmed by DRIFT spectroscopy, in agreement with successful intrapore amide grafting reactions.^21^ In addition, all hybrid materials revealed the presence of new C/H vibration bands. The obtained fine powders (white for **H1-Mg** and **H2-Al**, pale yellow for **H3-Ti** ^**+IV**^, and pale purple for **H4-Ti** ^**+III**^) were washed with an excess of toluene and *n*-hexane. The supernatant solutions were dried under reduced pressure, and the remaining solids were analyzed by ^1^H NMR experiments. Since the supernatants contained only the metal–organic precursors, the released pyrazoles Hpz^R2^ are retained at the surface, likely coordinating as a donor to the metal center.

**Scheme 1:**
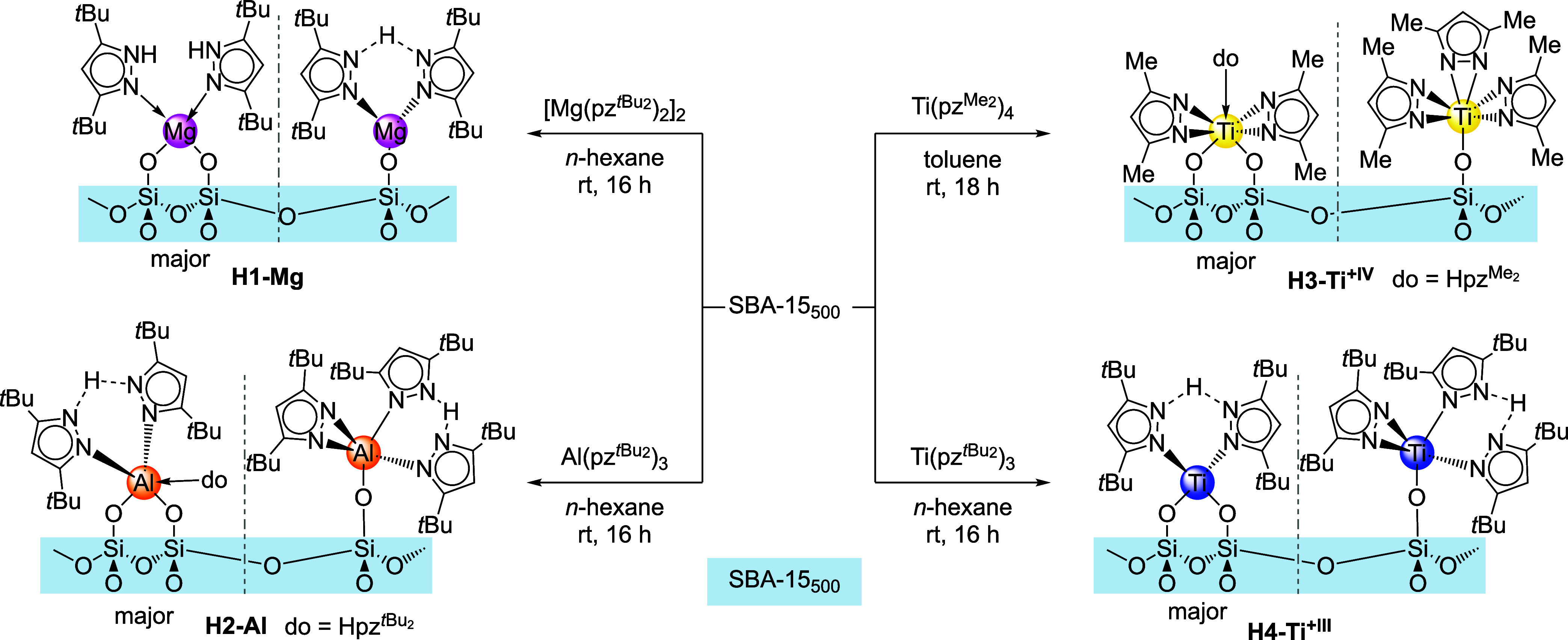
Grafting of Light Metal Pyrazolates onto Mesoporous Silica SBA-15_500_ and Proposed Surface Species Based on Elemental Analysis, ICP-OES, DRIFT, and Solid-State NMR Spectroscopy

The pyrazole originates from the protonolysis reaction with the Si-OH groups during the pyrazolate grafting process. The presence of Hpz^R2^ on the surface was further proven by ^1^H CP/MAS experiments, which revealed the pyrazole NH proton signal at δ = 12.66 ppm for **H1-Mg** and δ = 11.03 ppm for **H2-Al**. For **H3-Ti** ^**+IV**^, such a proton NMR signal was not perceived; however, the DRIFT spectrum featured a characteristic stretching vibration for N–H bonds at ṽ = 3396 cm^–1^. Conversely, the magnesium and aluminum hybrid materials did not show this vibrational band in the DRIFT spectra. The ^13^C CP/MAS spectra also confirmed the presence of pyrazolate/pyrazole species with typical signal sets. For **H1-Mg**, two signals for the carbon atoms in the 3/5-position of the pyrazolato ring were observed, and for **H3-Ti** ^**+IV**^, two signals for the ring proton in the 4-position. Both can originate from the pyrazolato ligand and the pyrazole donor. For all other carbon atoms of the pyrazolato/pyrazole ligands, only one broad signal was detected. CP/MAS experiments were not feasible for paramagnetic **H4-Ti** ^**+III**^.

ICP-OES experiments revealed metal contents of 1.85 wt% (**H1-Mg**), 1.21 wt% (**H2-Al**), 4.39 wt% (**H3-Ti** ^**+IV**^), and 2.66 wt% (**H4-Ti** ^**+III**^), respectively (see Table 1). Compared to the cerium-grafted materials [Ce^+IV^(pz^Me2^)_4_]_2_@SBA-15_500_ (10.80 wt%) and [Ce^+III^(pz^Me2^)_3_]_4_@SBA-15_500_ (22.19 wt%), the metal content is significantly lower, due to light metals used in this study and the higher-mass *t*Bu groups (except for **H3-Ti** ^**+IV**^, which bears Me groups).^24^ Taking into consideration the surface species proposed for similar hybrid materials,^24^ as well as the results obtained from C/H/N analyses, it is proposed that the surface species of **H1-Mg** consist of either a bipodal species coordinated by two donor pyrazoles of the type [(≡SiO)_2_Mg­(Hpz^*t*Bu2^)_2_] or a monopodal species of the type [(≡SiO)­Mg­(pz^*t*Bu2^)­(Hpz^*t*Bu2^)] (Scheme 1). Monopodal [(≡SiO)­Al­(pz^*t*Bu2^)_2_(Hpz^*t*Bu2^)] or bipodal surface species [(≡SiO)_2_Al­(pz^*t*Bu2^)­(Hpz^*t*Bu2^)_2_] are also favored for aluminum hybrid material **H2-Al**. The analytic results for the tetravalent titanium-based hybrid material **H3-Ti** ^**+IV**^ match closest the bipodal species [(≡SiO)_2_Ti^+IV^(pz^Me2^)_2_(Hpz^Me2^)]; however, the values do not fit as well as for the other materials, so a more complex mixture of surface species is highly likely. For trivalent **H4-Ti** ^**+III**^ a 1:1 mixture of monopodal [(≡SiO)­Ti^+III^(pz^*t*Bu2^)_2_(Hpz^*t*Bu2^)] and bipodal [(≡SiO)_2_Ti^+III^(pz^*t*Bu2^)­(Hpz^*t*Bu2^)] fits best to the analytical results. Due to the small ionic radii of the three metals employed (CN6; Mg: 0.72 Å, Al: 0.54 Å, Ti: 0.61 Å (+IV), 0.67 Å (+III)) a bimetallic surface species as it was observed for [Ln^+III^(pz^Me2^)_3_]_4_@SBA-15_500_ (Ln: La, Ce; CN6 La: 1.03 Å, Ce^+III^: 1.01 Å) seems unlikely.^24,32^ Further, the ceric material [Ce^+IV^(pz^Me2^)_4_]_2_@SBA-15_500_ (CN6 Ce^+IV^: 0.87 Å) was proposed to exclusively consist of a monometallic surface species. As mentioned above, the metal content of material **H3-Ti** ^**+IV**^ is approximately a third of the three times heavier Ce^+IV^ congener.^24^ A similar correlation is also found for magnesium-based **H1-Mg**. These similarities further indicate that in both cases a monometallic surface species is present. When considering the relatively low M/Si-OH content ratios (<0.35) of all hybrid materials, bipodal species constitute the majority of surface species in all cases. The N/M content ratios are in line with the species discussed above, suggesting two pyrazolato/pyrazole ligands for **H1-Mg** (3.43), three for **H2-Al** (5.67) and **H3-Ti** ^**+IV**^ (5.4), and between two and three for **H4-Ti** ^**+III**^ (4.63). A more precise categorization of the surface species is difficult, especially because several other surface species (not depicted in Scheme 1) could coexist.

The N_2_ physisorption experiments revealed a drastic reduction of the surface area of the hybrid materials (ca. 50%, 397–418 m^2^/g, see Table 1 and Figure 1) compared with the parent material. As expected, a similar decrease can be observed for the pore volume (0.53–0.57 cm^3^/g) and the pore diameter (6.2–6.5 nm). The titanium material Ti^+IV^(pz^Me2^)_4_@SBA-15_500_ (**H3-Ti** ^**+IV**^) features the comparatively smallest decrease in the pore diameter, which is in line with the small Me moieties at the pyrazolato ligand. This change in pore diameter is comparable to [Ce^+IV^(pz^Me2^)_4_]_2_@SBA-15_500_, which further suggests a monometallic surface species. For trivalent rare-earth-metal pyrazolate materials containing bimetallic surface species, a significantly higher decrease of the surface area was observed.^24^ More precisely, [Ce^+III^(pz^Me2^)_3_]_4_@SBA-15_500_ revealed a surface change to less than a quarter of that of the parent material.

**Figure 1:**
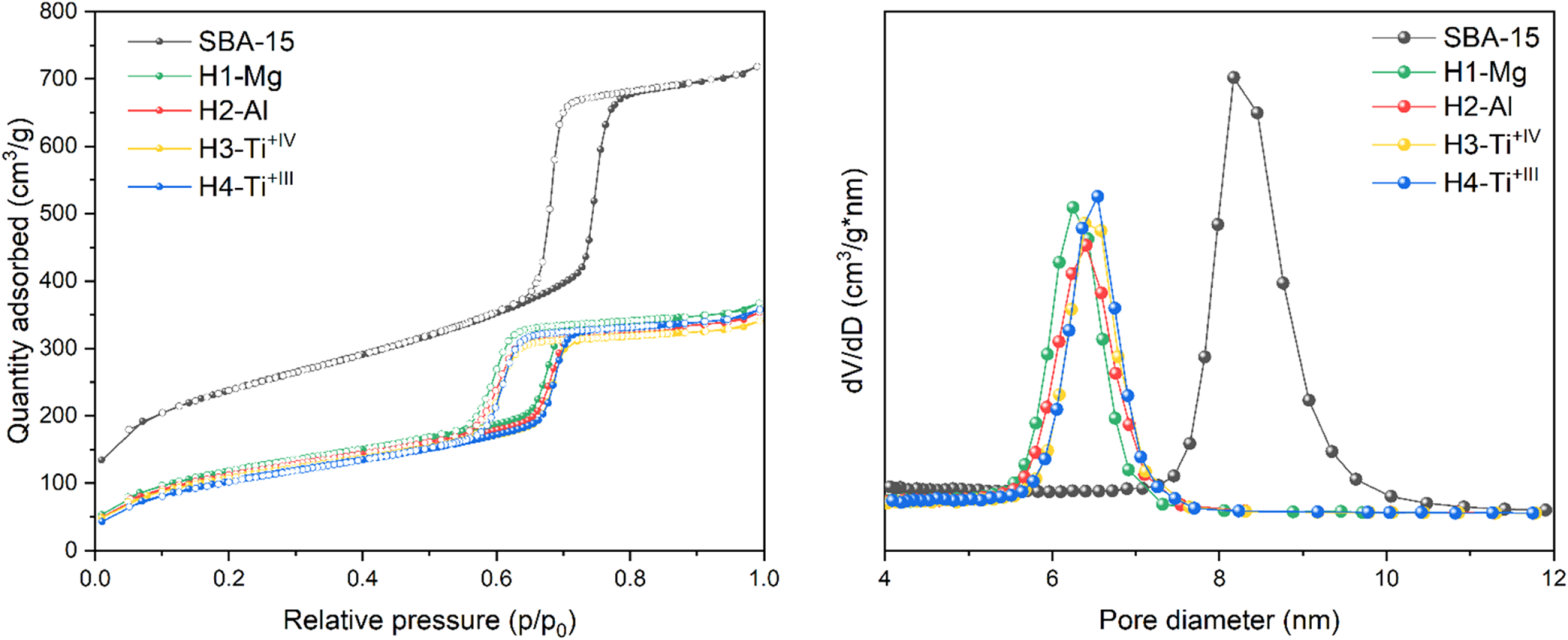
N_2_ physisorption isotherms (left) and pore size distribution (right) of parent material SBA-15 (black), [Mg­(pz^tBu2^)_2_]_2_@SBA-15_500_ (**H1-Mg**, green), Al­(pz^tBu2^)_3_@SBA-15_500_ (**H2-Al**, red), Ti^+IV^(pz^Me2^)_4_@SBA-15_500_ (**H3-Ti^+IV^**, yellow), and Ti^+III^(pz^tBu2^)_4_@SBA-15_500_ (**H4-Ti^+III^**, blue).

### Carbon Dioxide Uptake by Mesoporous Hybrid Materials

When exposed to an atmosphere of 1 bar of CO_2_, a significant mass gain for three hybrid materials was observed, with 7% for **H1-Mg**, 6% for **H2-Al**, and 11% for **H3-Ti** ^**+IV**^ (see Table 1). Further, solid-state NMR/DRIFT spectroscopy and elemental analyses confirmed a successful CO_2_ insertion and the formation of hybrid materials denoted as CO_2_@[Mg­(pz^*t*Bu2^)_2_]_2_@SBA-15_500_ (**CO**_**2**_ **@H1-Mg**), CO_2_@Al­(pz^*t*Bu2^)_3_@SBA-15_500_ (**CO**_**2**_ **@H2-Al**), and CO_2_@Ti^+IV^(pz^Me2^)_4_@SBA-15_500_ (**CO**_**2**_ **@H3-Ti** ^**+IV**^). The DRIFT spectra of **CO**_**2**_ **@H2-Al** and **CO**_**2**_ **@H3-Ti** ^**+IV**^ revealed the characteristic C–O double bond stretching vibration for pyrazolato-inserted CO_2_ at ṽ = 1786 and 1744 cm^–1^, respectively (Figures S48/S52). For magnesium-based **CO**_**2**_ **@H1-Mg**, a broad signal at ṽ = 1655 cm^–1^ was observed, which is indicative of a C–O stretching vibration of κ^2^(O,O) coordinated carboxylate ligands.^33^ Such a stretch vibration band would also be typical of carbamato ligands bridging between two metal centers. However, as discussed above, the existence of a bimetallic surface species is highly unlikely. Pyrazole donors are also able to insert CO_2_ under the formation of a carbamic acid when coordinated to a metal center.^27,28^ The carbamic acid is able to form a carboxylic group via isomerization. This corroborates that the surface species of **H1-Mg** mostly consist of a bipodal magnesium center stabilized by two pyrazole donors, [(≡SiO)_2_Mg­(Hpz^*t*Bu2^)_2_].

The ^13^C CP/MAS spectrum of **CO**_**2**_ **@H1-Mg** (Figures S10/S11) is almost indistinguishable from that of **H1-Mg** (Figure S2). The characteristic splitting of the carbon signal at the pyrazolato ring upon CO_2_ insertion is not detectable, because two signals already appeared for **H1-Mg** in this region. However, for **CO**_**2**_ **@H2-Al** and **CO**_**2**_ **@H3-Ti** ^**+IV**^, this characteristic splitting into two distinct signals can be observed, which further confirms the successful CO_2_ uptake. In the case of **CO**_**2**_ **@H2-Al**, a signal for the carbon atom of the inserted CO_2_ was observed at δ = 144.9 ppm, which is reminiscent of the signal found for solvated [Al­(CO_2_·pz^*t*Bu2^)_2_(pz^*t*Bu2^)] at δ = 146.9 ppm.^28^

In contrast to the three materials discussed above, trivalent **H4-Ti** ^**+III**^ did not show any mass gain when exposed to a CO_2_ atmosphere. Only a color change from light purple to yellow was observed, which indicates the oxidation of the metal center. Furthermore, the DRIFT spectrum and elemental analyses were almost identical to the starting material, in agreement with the absence of CO_2_ insertion. This reaction behavior similar to the oxo-bridged tetravalent complex, O­[Ti­(pz^*t*Bu2^)_3_]_2_, which did not show any CO_2_ insertion. The [OTi­(pz^*t*Bu2^)_3_] moiety of this complex is reminiscent of the surface species of **H4-Ti** ^**+III**^ after a possible oxidation of the metal center.^29^ The steric bulk of the *t*Bu groups might hinder the insertion,^34^ because both the complex Ti^+IV^(η^2^-pz^Me2^)_4_ and material **H3-Ti** ^**+IV**^ bearing Me groups are able to insert CO_2_. Such a pyrazolato-centered steric criterion might in part apply for the CO_2_ insertion at the grafted species **H1-Mg** and **H4-Ti** ^**+III**^ with the comparatively larger magnesium center (CN6; Mg: 0.72 Å, Ti­(+III): 0.67 Å). However, given the larger size of titanium­(III) compared to aluminum­(III) (CN6; Al: 0.54 Å), enhanced steric repulsion at the Ti­(III) surface centers might originate from additional significant interactions with surface siloxane species (oxophilicity criterion: Θ = 1.0 (Ti), 0.8 (Al), 0.6 (Mg))^35^ and the distinct M–N bonding of transition metals.

### A Mixed Pyrazolato/Siloxy Titanium Complex as a Surface Model

Complexes bearing *tert*-butoxysiloxy moieties are often used as model complexes for silica surface species.^24,36–41^ Initially, the synthesis of a mixed titanium pyrazolato/tris­(*tert*-butoxy)­siloxy titanium complex was attempted by applying a protonolysis protocol with Ti^+IV^(pz^Me2^)_4_ and HOSi­(O*t*Bu)_3_. However, the obtained crystalline product was a mixture of a titanium complex and formed Hpz^Me2^, which could not be separated by fractional crystallization. Therefore, we chose the detour via the literature known titanium amide Ti^+IV^(NMe_2_)­[OSi­(O*t*Bu)_3_]_3_ (Scheme 2).^41^ A subsequent transamination of Ti^+IV^(NMe_2_)­[OSi­(O*t*Bu)_3_]_3_ with pyrazole Hpz^Me2^ gave the mixed pyrazolato/tris­(*tert*-butoxy)­siloxy model complex Ti^+IV^(pz^Me2^)­[OSi­(O*t*Bu)_3_]_3_ (**M1-Ti** ^**+IV**^) as colorless crystals. The connectivity obtained from a single-crystal X-ray diffraction measurement revealed that the titanium center in **M1-Ti** ^**+IV**^ adopts a tetrahedral geometry (Figure 2).

**Figure 2:**
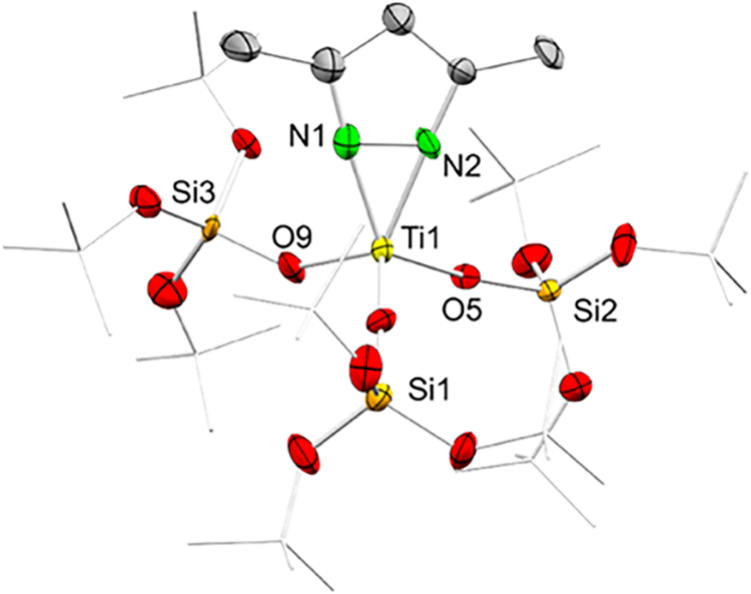
Connectivity of Ti^+IV^(pz^Me2^)­[OSi­(O*t*Bu)_3_]_3_ (**M1-Ti^+IV^**); thermal ellipsoids are set at 50% probability; hydrogen atoms are omitted, and *t*Bu moieties are set as wireframe for clarity.

**Scheme 2:**
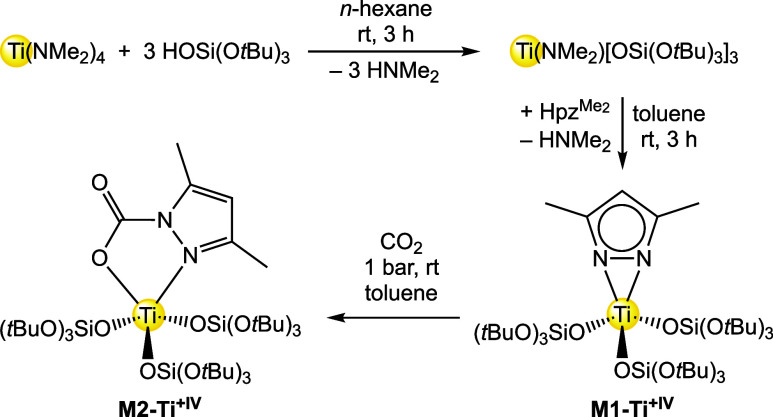
Synthesis of Ti^+IV^(pz^Me2^)­[OSi­(O*t*Bu)_3_]_3_ (M1-Ti^+IV^) and Subsequent Reaction with CO_2_ to Ti^+IV^(CO_2_·pz^Me2^)­[OSi­(O*t*Bu)_3_]_3_ (M2-Ti^+IV^)

The ^1^H NMR spectrum of **M1-Ti** ^**+IV**^ shows the expected three signals, two resonances of the pyrazolato ligand, one for the ring proton at δ = 6.11 ppm and one for the Me groups at δ = 2.42 ppm, as well as the resonance of *t*Bu methyl groups at δ = 1.45 ppm (Figure S16). The ^13^C NMR spectrum of **M1-Ti** ^**+IV**^ revealed three signals for the pyrazolato ligand and two signals for the siloxy ligand. The ring carbon atoms of the pyrazolato ligand were detected at δ = 116.0 ppm (4-position) and at δ = 146.3 ppm (3/5-position). The two pyrazolato Me groups appeared as one signal at δ = 13.2 ppm (Figure S17).

When compound **M1-Ti** ^**+IV**^ was exposed to an atmosphere of 1 bar CO_2_ in toluene-*d*_8_ in a NMR tube equipped with a J.Young valve, the formation of a new signal set could be observed in both ^1^H and ^13^C NMR spectra (see S18–S21). These signatures can be assigned to the CO_2_ insertion complex Ti^+IV^(CO_2_·pz^Me2^)­[OSi­(O*t*Bu)_3_]_3_ (**M2-Ti** ^**+IV**^). The ^1^H NMR spectrum revealed that the signal for the carbamato Me groups splits up into two distinct singlets, δ = 2.75 and 2.30 ppm, respectively, due to the introduced asymmetry upon CO_2_ insertion. Similarly, in the ^13^C NMR spectrum, the signals for the quaternary carbon atoms as well as the methyl groups split up into two distinct signals at δ = 151.9 and 142.7 ppm and at δ = 15.8 and 11.9 ppm, respectively. In addition, a signal for a carbamate-type species was detected at δ = 149.4 ppm, which is reminiscent of other titanium carbamate complexes.^29^ However, only 15% of **M1-Ti** ^**+IV**^ was converted into **M2-Ti** ^**+IV**^ at ambient temperature. The conversion could be further increased to 30% when the temperature was decreased to 0 °C, but further cooling did not improve the efficiency of the CO_2_ insertion reaction. Up to this date, no crystalline material of **M2-Ti** ^**+IV**^ could be obtained.

### Catalytic Conversion of CO_2_ and Epoxides to Cyclic Carbonates

The new hybrid materials were tested as catalysts in the conversion of CO_2_ and epoxides to cyclic carbonates. Initial reactions were conducted with a catalytic load of 0.5 mol% metal center of the hybrid material, 0.5 mol% of the cocatalyst tetra-*n*-butyl ammonium bromide/iodide (TBAB/TBAI), at ambient temperature, 1 bar of CO_2_, and in neat epoxide for 24 h. In later conversions, the temperature was increased to 90 °C and the pressure was increased to 10 bar of CO_2_. The four epoxides propylene oxide (PO), styrene oxide (SO), 3,3-dimethyl-1,2-epoxybutane (*t*BO), and 1,2-epoxyhexane (EH) were employed as substrates.

The magnesium hybrid material **H1-Mg** converted PO into a mixture of the desired propylene carbonate and a polymeric side product (likely a polyether) when TBAB was used as cocatalyst (see Table 2, entry 1). The conversion of 63% is not representative due to the formation of the side product. Changing the cocatalyst to TBAI drastically increased the selectivity, and the cyclic carbonate was almost exclusively formed (entry 2). The catalytic conversion of 88% is significantly higher than the 56% achieved by the solvated molecular [Mg­(pz^*t*Bu2^)_2_]_2_ congener.^27^ Strikingly, in the absence of cocatalyst, the selectivity is reversed and the polymeric product was formed exclusively (entry 3). Hence, not only could the catalytic conversion be significantly increased when the magnesium pyrazolate is immobilized on the PMS material, but also the selectivity appeared to be tunable by the choice of cocatalyst. In contrast, the immobilized cerium pyrazolates have similar conversions as their molecular congeners.^24^ At 90 °C and 10 bar CO_2_, the catalytic activity of **H1-Mg** could be increased, resulting in conversions of >99% for PO as well as the bulkier SO and EH (entry 4). For the even bulkier *t*BO, a conversion of 60% was achieved. These findings are similar to those achieved for the cerium hybrid material [Ce^+IV^(pz^Me2^)_4_]_2_@SBA-15_500_ (entry 10). The significantly increased catalytic activity of **H1-Mg** compared to molecular [Mg­(pz^*t*Bu2^)_2_]_2_ might be explained by the highly active surface species [(≡SiO)_2_Mg­(Hpz^*t*Bu2^)_2_], which is coordinated only by donor pyrazoles. Initially formed carbamic acid could easily dissociate from the metal centers. A quantitative conversion equals a turnover number (TON) of 198 and a turnover frequency (TOF) of 8 h^–1^. When the catalytic load was reduced to 0.01 mol% metal center, the TON could be increased to 2100, affording a TOF of 88 h^–1^ at 90 °C (entry 5). In general, surface-grafted metal centers can exhibit increased reactivity/Lewis acidity originating from site isolation/surface confinement/distorted coordination geometry and the electron-withdrawing effect of the activated silica surface.^20^ A catalytic scenario for the surface species [(≡SiO)_2_Mg­(Hpz^*t*Bu2^)_2_] of material **H1-Mg** is proposed in Figure S59.

**Table 2:** Catalytic Performance of Light Metal Pyrazolates Grafted onto SBA-15 Mesoporous Silica in the Cycloaddition of CO_2_ and Epoxides to Cyclic Carbonates^a^,^b^

entry	catalyst	mol%/*T*/*p* [bar]	PO	SO	*t*BO	EH	cocatalyst
1	**H1-Mg**	0.5/26 °C/1	63%^c^				TBAB
2	**H1-Mg**	0.5/26 °C/1	88%				TBAI
3	**H1-Mg**	0.5/26 °C/1	<1^d^				
4	**H1-Mg**	0.5/90 °C/10	>99%	99%	60%	99%	TBAI
5	**H1-Mg**	0.01/90 °C/10	21%				TBAI
6	**H2-Al**	0.5/26 °C/1	48%^e^				TBAB
7	**H3-Ti** ^**+IV**^	0.5/26 °C/1	15%				TBAB
8^f^	**H3-Ti** ^**+IV**^	0.5/90 °C/10	97%	91%	41%	74%	TBAB
9	**H4-Ti** ^**+III**^	0.5/26 °C/1	46%^g^				TBAB
10^h^	**[Ce(pz** ^**Me2**^ **)**_**4**_ **]**_**2**_ **@SBA-15**_**500**_ ^h^	0.5/90 °C/10	>99%	91%	66%	94%	TBAB

a Reaction conditions not stated in the table: [cocatalyst] = [catalyst (referred to metal center)], 24 h, neat in epoxide.

b Conversion determined by comparison of the proton integrals in the α-position of the epoxide and the corresponding cyclic carbonate (except for *t*BO, where the integral of the *t*Bu moieties was used).

c Not representative due to polymer formation as side reaction.

d Polymeric product.

e Not representative because of the formation of an unidentified side product.

f Hybrid material **H3-Ti** ^**+IV**^ is thermally stable under these conditions; molecular Ti^+IV^(pz^Me2^)_4_ is not released at 90 °C/10^–2^ mbar/6 h, although it can be sublimed under these conditions.

g Oxidation to a Ti^+IV^ species occurred.

h Previous work.^24^

The aluminum-based hybrid material **H2-Al** exhibited a lower conversion of 48% for PO (entry 6). However, this conversion is not representative due to the formation of a brown side product, which could not be further identified (see Figure S29). The titanium-based hybrid material **H3-Ti** ^**+IV**^ showed a low PO conversion of 15% at ambient temperature (entry 7). However, at 90 °C and 10 bar, the conversion could be drastically increased to 97% (entry 8). Even for the bulkier epoxides SO (91%), *t*BO (41%), and EH (74%), high conversions could be obtained. For trivalent **H4-Ti** ^**+III**^, an immediate color change of the material from light purple to yellow was observed as soon as the material was in contact with PO, indicative of instant oxidation of the titanium center. However, the catalytic conversion of this material reached 46% at ambient conditions (entry 9), which is higher than that for tetravalent **H3-Ti** ^**+IV**^ under the same conditions.

To examine the recyclability of the hybrid materials, cycling experiments were conducted with **H1-Mg** as the best catalyst system of this study. The catalytic conversion of CO_2_ and PO was conducted in eight consecutive cycles at ambient temperature and 1 bar of CO_2_ with TBAI as the cocatalyst (Figure 3). After each cycle, the catalyst was recovered by centrifugation, washed three times with Et_2_O, and dried under reduced pressure.

**Figure 3:**
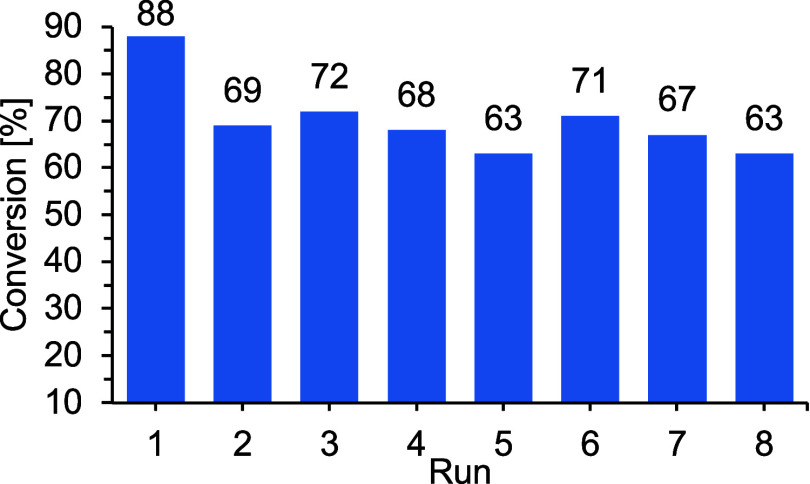
Catalytic conversion of propylene oxide and CO_2_ upon recycling by using 0.5 mol% of **H1-Mg** and 1.0 mol% TBAI as cocatalyst at 1 bar CO_2_ pressure and ambient temperature.

After an initial drop of ∼20% (from 88% in the first run to 69% in the second run), the catalytic conversion stayed consistent between 63 and 72%. This initial drop in catalytic conversion can be explained by the formation of carbamic acid HO_2_Cpz^*t*Bu2^ via CO_2_ insertion into donor Hpz^*t*Bu2^, which was observed in earlier studies.^27–29^ Since the ^1^H NMR spectrum of the supernatants of the initial crude reaction mixture did not show any signals of the carbamic acid, it is retained on the surface, which most likely reduces the catalytic activity. To clarify that the loss of catalytic activity is not caused by leaching of the magnesium species from the material surface, ICP-OES analysis of the supernatants was conducted in the first six cycles (see Table S1). The results showed that either only traces or no detectable amounts of magnesium were leached from the surface. In total, only a loss of 0.09% of the initial magnesium mass could be detected. In addition, the comparison of the DRIFT spectra of fresh **H1-Mg** and used **H1-Mg** after eight catalytic cycles did not show any difference in signals besides the appearance of a signal for the C–O double bond stretching vibration at ṽ = 1784 cm^–1^, which can be assigned to propylene carbonate (see S40, S56, and S57). To further rule out the presence of a catalytically active species in the supernatant solution, the conversion was stopped after 6 h, and the insoluble material was filtered off. The remaining supernatant solution was set under a CO_2_ atmosphere for an additional 18 h. During this time, the conversion only slightly increased (∼1%), which can be attributed to the presence of the cocatalyst (S36–S38). Accordingly, the leaching of magnesium species seems highly unlikely. Therefore, it can be hypothesized that the drop in catalytic conversion is caused by the formation of the carbamic acid, small losses of the fine powdered material during the washing process, as well as coordination of propylene carbonate to the metal centers, which has been reported previously.^22,24,25,42^

The catalytic performance of the hybrid materials under study is comparable to other reported SOMC systems based on light metals.^22,25,42–47^ For example the titanium-based material Ti­(O*n*Bu)_4_@SBA-15 and its organo-functionalized derivatives R@Ti­(O*n*Bu)_4_@SBA-15 (R = 3-amino, 3-adenino or (3-chloropropyl)­triethoxysilane) achieved TOFs of 3.0 and 7.3 h^–1^, respectively, for epichlorohydrin at 120 °C and 6.9 bar CO_2_.^44^ The immobilized rare-earth metal chloride YCl_3_@SiO_2‑700_ showed a quantitative conversion of PO with TBAI as a cocatalyst at already mild conditions of 60 °C and 2 bar CO_2_.^22^ Recently, we reported the immobilized di/trivalent rare-earth-metal silylamides HOEt@Ln­(N­(SiMe_3_)_2_)_*x*_@PMS (Ln = Sm­(II), Sm­(III), Yb­(II); PMS = SBA-15_500_, MCM-41_500_, MCM-48_500_), which also accomplished quantitative conversions for PO, EH and epichlorohydrin with TBAI as cocatalyst at 80 °C and 10 bar CO_2_.^25^

## Conclusions

The light metal pyrazolates [Mg­(pz^*t*Bu2^)_2_]_2_, Al­(pz^*t*Bu2^)_3_, Ti^+IV^(pz^Me2^)_4_, and Ti^+III^(pz^*t*Bu2^)_3_ were grafted for the first time onto the surface of periodic mesoporous silica. The “pseudo” surface organometallic chemistry approach gave the hybrid materials [Mg­(pz^*t*Bu2^)_2_]_2_@SBA-15_500_, Al­(pz^*t*Bu2^)_3_@SBA-15_500_, Ti^+IV^(pz^Me2^)_4_@SBA-15_500_, and Ti^+III^(pz^*t*Bu2^)_3_@SBA-15_500_. A comprehensive physicochemical characterization and analysis of the new materials suggest the occurrence of effective intrapore chemistry with high metal loadings and the preservation of mesoporosity as featured by site-isolated monometallic surface species. Depending on the pyrazolato content, a CO_2_ uptake of up to 11 wt% can be achieved, as revealed for Ti^+IV^(pz^Me2^)_4_@SBA-15_500_. Such CO_2_ uptake is comparable to that observed for amino-functionalized SBA-15 materials.^48^ The mixed pyrazolato/siloxy complex Ti^+IV^(pz^Me2^)­[OSi­(O*t*Bu)_3_]_3_ could be obtained as a molecular model for potential surface species, being capable of CO_2_ insertion. All reported hybrid materials are active catalysts in the cycloaddition of epoxides and CO_2_ to cyclic carbonates. The magnesium hybrid material [Mg­(pz^*t*Bu2^)_2_]_2_@SBA-15_500_ with the highly active surface species [(≡SiO)_2_Mg­(Hpz^*t*Bu2^)_2_] showed a significant increase in catalytic activity when compared to its homogeneous congeners even for bulky epoxides, in addition to a high stability of conversion over eight consecutive catalytic runs. Finally, the selectivity of products (cyclic carbonate via polymeric product) could be adjusted with the choice of cocatalyst.

## Experimental Section

### General Considerations

No uncommon hazards are noted. All manipulations were performed under rigorous exclusion of air and moisture under argon atmosphere (<0.1 ppm of O_2_, <0.1 ppm of H_2_O) in an MB200B glovebox (MBraun) or according to standard Schlenk techniques in oven-dried glassware. Solvents (THF, Et_2_O *n*-pentane, *n*-hexane, and toluene) were purified by using Grubbs-type columns (MBraun SPS-800, solvent purification system) and stored inside a glovebox. THF was dried further over molecular sieves. [D_8_]­toluene, C_6_D_6_, and [D_8_]­THF were obtained from Sigma-Aldrich and dried over Na/K alloy and filtered prior to use. 3,5-Dimethyl pyrazole (99%), hydrazine hydrate, styrene oxide (>97%, degassed prior to use), tetrakis­(dimethylamido)titanium (99.999%), 2,2,6,6-tetramethylheptane-3,5-dione, and tris­(*tert*-butoxy)­silanol (99.999%) were purchased from Sigma-Aldrich and used as received. Propylene oxide was purchased from Acros Organics, and the former was degassed prior to use. 2-*tert*-Butyloxirane was purchased from abcr and degassed prior to use. 1,2-Epoxyhexane (97%) was purchased from Thermo Fischer and degassed prior to use. [Mg­(pz^*t*Bu2^)_2_]_2_,^49^ Al­(pz^*t*Bu2^)_3_,^50^ Ti^+IV^(pz^Me2^)_4_,^51^ Ti^+III^(pz^*t*Bu2^)_3_,^52^ Ti^+IV^(NMe_2_)­[OSi­(O*t*Bu)_3_]_3_,^41^ and SBA-15 ^31^ were synthesized according to literature procedures. Argon and CO_2_ were supplied by Westfalen AG. Solution ^1^H and ^13^C spectra were recorded in NMR tubes equipped with a J.Young valve on a Bruker AVII+400 spectrometer (^1^H: 400.11 MHz; ^13^C: 100.61 MHz) at 299 K. The chemical shifts listed in the experimental section are referenced to solvent residual resonances in parts per million in relation to tetramethylsilane. Solid-state NMR spectra were obtained at ambient temperature on a Bruker AVIIIHD-300WB spectrometer (^1^H: 300.13 MHz, ^13^C: 75.47 MHz, ^29^Si: 59.62 MHz) equipped with MAS (magic angle spinning) hardware using a ZrO_2_ rotor with an outside diameter of 4 mm. IR spectra were recorded on a Bruker Invenio R spectrometer (Bruker). The samples were mixed with KBr powder and measured in a DRIFT cell with KBr windows. DRIFT data were converted using the Kubelka-Munk refinement. N_2_ physisorption measurements were carried out on an ASAP2020 volumetric adsorption apparatus (Micromeritics Instrument Corp.) at 77 K (*a*_m_(N_2_, 77 K) = 0.162 nm^2^). The samples were degassed at <5 μmHg and ambient temperatures for 2 h, prior to analysis. The Brunauer–Emmett–Teller (BET) specific surface area was calculated from the nitrogen adsorption branch of the isotherm in the relative pressure range of 0.07–0.15.^53^ Pore size distributions (d*V*/d*D*) were calculated from the nitrogen desorption branch using the Barrett–Joyner–Halenda (BJH) method.^54^ The low-angle powder X-ray diffraction (PXRD) pattern was recorded on a Bruker Advance D8 instrument using monochromatic Cu Kα radiation (λ = 1.5406 Å) in the 2θ range of 0.50–9.99° with a scan speed of 2 s per step. Elementary analyses were performed on an Elementar vario MICRO cube in CHNS mode. ICP-OES measurements were conducted on a Thermo Scientific iCAP 7000 series.

### [Mg­(pz^*t*Bu2^)_2_]_2_@SBA-15_500_ (**H1-Mg**)

SBA-15_500_ (367 mg, 1.03 mmol of surface SiOH) was suspended in 2 mL of *n*-hexane, and a solution of [Mg­(pz^*t*Bu2^)_2_]_2_ (394 mg, 0.52 ≡ 1.03 mmol Mg center) in 8 mL of *n*-hexane was added dropwise. The suspension was stirred for 16 h at ambient temperature. The hybrid material was separated by centrifugation and was washed two times with toluene (5 mL) and five times with *n*-hexane (5 mL). The solvent was removed under reduced pressure, and **H1-Mg** was obtained as a colorless powder (426 mg). The supernatant solution was filtered, and the solvent was removed under reduced pressure. ^1^H NMR spectrum revealed the recovery of some magnesium pyrazolate precursor. ^1^H CP/MAS spectrum (300.13 MHz, MAS at 8 kHz): δ = 12.66 (N*H*), 6.94 (4-*H*(pz)), 5.81 (4-*H*(pz)), 1.03 (C­(C*H*_3_)_3_) ppm. ^13^C CP/MAS spectrum (75.47 MHz, MAS at 8 kHz): δ = 165.1 (3/5-*C*(pz)), 155.4 (3/5-*C*(pz)), 99.0 (4-*C*(pz)), 29.4 (*C*(*C*H_3_)_3_) ppm. ^29^Si CP/MAS spectrum (59.63 MHz, MAS at 5 kHz): δ = −101.6 ppm. *a*_BET_ = 410 m^2^/g, *V*_pore_ = 0.57 cm^3^/g, and *d*_pore_ = 6.2 nm. DRIFTS: ṽ = 2967 (w), 2911 (vw), 2873 (vw), 1560 (vw), 1510 (vw), 1491 (vw), 1467 (vw), 1367 (vw), 1281 (vw), 1200 (m), 1079 (vs), 807 (vw), 457 (w) cm^–1^. Elemental analysis found: Mg 1.85 (ICP-OES); C 19.25, H 2.67, N 3.71.

### Al­(pz^*t*Bu2^)_3_@SBA-15_500_ (**H2-Al**)

SBA-15_500_ (221 mg, 620 μmol of surface SiOH) was suspended in 2 mL of *n*-hexane, and a solution of Al­(pz^*t*Bu2^)_3_ (350 mg, 620 μmol) in 8 mL of *n*-hexane was added dropwise. The suspension was stirred for 16 h at ambient temperature. The hybrid material was separated by centrifugation and was washed two times with toluene (5 mL) and five times with *n*-hexane (5 mL). The solvent was removed under reduced pressure, and **H2-Al** was obtained as a colorless powder (239 mg). The supernatant solution was filtered, and the solvent was removed under reduced pressure. ^1^H NMR spectrum revealed the recovery of some aluminum pyrazolate precursor. ^1^H CP/MAS spectrum (300.13 MHz, MAS at 8 kHz): δ = 11.03 (N*H*), 6.87 (4-*H*(pz)), 5.73 (4-*H*(pz)), 0.97 (C­(C*H*_3_)_3_) ppm. ^13^C CP/MAS spectrum (75.47 MHz, MAS at 8 kHz): δ = 161.7 (3/5-*C*(pz)), 99.3 (4-*C*(pz)), 30.9 (*C*(CH_3_)_3_), 29.4 (C­(*C*H_3_)_3_) ppm. ^29^Si CP/MAS spectrum (59.63 MHz, MAS at 5 kHz): δ = −101.6 ppm. *a*_BET_ = 418 m^2^/g, *V*_pore_ = 0.55 cm^3^/g, and *d*_pore_ = 6.4 nm. DRIFTS: ṽ = 2968 (vw), 2911 (vw), 2873 (vw), 1561 (vw), 1467 (vw), 1368 (vw), 1187 (m), 1075 (vs), 814 (vw), 454 (w) cm^–1^. Elemental analysis found: Al 1.21 (ICP-OES); C 19.03, H 2.88, N 3.55.

### Ti^+IV^(pz^Me2^)_4_@SBA-15_500_ (**H3-Ti^+IV^**)

SBA-15_500_ (337 mg, 943 μmol of surface SiOH) was suspended in 2 mL toluene, and a solution of Ti^+IV^(pz^Me2^)_4_ (404 mg, 943 μmol) in 8 mL toluene was added dropwise. The suspension was stirred for 18 h at ambient temperature. The hybrid material was separated by centrifugation and was washed eight times with toluene (5 mL) and three times with *n*-hexane (5 mL). The solvent was removed under reduced pressure, and **H3-Ti** ^**+IV**^ was obtained as a pale-yellow powder (392 mg). The supernatant solution was filtered, and the solvent was removed under reduced pressure. ^1^H NMR spectrum revealed the recovery of some titanium pyrazolate precursor. ^1^H CP/MAS spectrum (300.13 MHz, MAS at 8 kHz): δ = 6.75 (4-*H*(pz)), 5.67 (4-*H*(pz)), 1.84 (C*H*_3_) ppm. ^13^C CP/MAS spectrum (75.47 MHz, MAS at 8 kHz): δ = 146.3 (3/5-*C*(pz)), 113.9 (4-*C*(pz)), 104.5 (4-*C*(pz)), 10.6 (*C*H_3_) ppm. ^29^Si CP/MAS spectrum (59.63 MHz, MAS at 5 kHz): δ = −103.4 ppm. *a*_BET_ = 405 m^2^/g, *V*_pore_ = 0.53 cm^3^/g, and *d*_pore_ = 6.4 nm. DRIFTS: ṽ = 3396 (vw), 2931 (vw), 2871 (vw), 1572 (vw), 1526 (vw), 1440 (vw), 1422 (vw), 1374 (vw), 1074 (vs), 804 (w), 460 (w) cm^–1^. Elemental analysis found: Ti 4.39 (ICP-OES); C 17.36, H 2.35, N 7.00.

### Ti^+III^(pz^*t*Bu2^)_3_@SBA-15_500_ (**H4-Ti^+III^**)

SBA-15_500_ (183 mg, 512 μmol of surface SiOH) was suspended in 2 mL of *n*-hexane, and a solution of Ti^+III^(pz^*t*Bu2^)_3_ (300 mg, 512 μmol) in 8 mL of *n*-hexane was added dropwise. The suspension was stirred for 18 h at ambient temperature. The hybrid material was separated by centrifugation and washed two times with toluene (5 mL) and five times with *n*-hexane (5 mL). The solvent was removed under reduced pressure, and **H4-Ti** ^**+III**^ was obtained as a pale-purple powder (221 mg). The supernatant solution was filtered, and the solvent was removed under reduced pressure. ^1^H NMR spectrum revealed the recovery of some titanium­(III) pyrazolate precursor. *a*_BET_ = 358 m^2^/g, *V*_pore_ = 0.55 cm^3^/g, and *d*_pore_ = 6.5 nm. DRIFTS: ṽ = 2967 (vw), 2909 (vw), 2872 (vw), 1561 (vw), 1511 (vw), 1487 (vw), 1464 (vw), 1435 (vw), 1366 (vw), 1194 (s), 1144 (s), 1075 (vs), 1057 (vs), 807 (vw), 561 (vw), 470 (w), 446 (vw) cm^–1^. Elemental analysis found: Ti 2.66 (ICP-OES); C 18.99, H 2.70, N 3.58.

### CO_2_@[Mg­(pz^*t*Bu2^)_2_]_2_@SBA-15_500_ (**CO_2_@H1-Mg**)

A Schlenk tube was loaded with solid **H1-Mg** (100.7 mg), and the atmosphere was changed to 1 bar CO_2_. After 3 h, **CO**_**2**_ **@H1-Mg** could be collected as a white powder (108.8 mg). ^1^H CP/MAS spectrum (300.13 MHz, MAS at 8 kHz): δ = 12.12 (COO*H*/N*H*), 6.91 (4-*H*(pz)), 5.77 (4-*H*(pz)), 1.03 (C­(C*H*_3_)_3_) ppm. ^13^C CP/MAS spectrum (75.47 MHz, MAS at 8 kHz): δ = 163.3 (3/5-*C*(pz)), 156.0 (3/5-*C*(pz)), 98.1 (4-*C*(pz)), 30.6 (*C*(CH_3_)_3_), 29.0 (C­(*C*H_3_)_3_) ppm. DRIFTS: ṽ = 2367 (w), 2911 (vw), 2874 (vw), 1655 (vw), 1560 (vw), 1491 (vw), 1467 (vw), 1368 (vw), 1281 (vw), 1189 (s), 1080 (vs), 806 (w), 461 (w) cm^–1^. Elemental analysis found: C 18.47, H 3.19, N 3.66.

### CO_2_@Al­(pz^*t*Bu2^)_3_@SBA-15_500_ (**CO_2_@H2-A**l)

A Schlenk tube was loaded with solid **H2-Al** (70.6 mg), and the atmosphere was changed to 1 bar CO_2_. After 3 h, **CO**_**2**_ **@H2-Al** could be collected as a white powder (75.3 mg). ^1^H CP/MAS spectrum (300.13 MHz, MAS at 8 kHz): δ = 11.15 (COO*H*/N*H*), 6.75 (4-*H*(pz)), 5.86 (4-*H*(pz)), 1.08 (C­(C*H*_3_)_3_) ppm. ^13^C CP/MAS spectrum (75.47 MHz, MAS at 8 kHz): δ = 163.4 (3-*C*(pz)), 156.3 (5-*C*(pz)), 144.9 (*C*O_2_), 107.2 (4-*C*(pz)), 98.5 (4-*C*(pz)), 30.6 (*C*(CH_3_)_3_), 29.1 (C­(C*H*_3_)_3_) ppm. DRIFTS: ṽ = 2967 (w), 2911 (vw), 2875 (vw), 1786 (vw), 1760 (vw), 1561 (vw), 1490 (vw), 1467 (vw), 1368 (vw), 1333 (vw), 1191 (s), 1075 (vs), 812 (w), 461 (m) cm^–1^. Elemental analysis found: C 17.81, H 2.91, N 3.56.

### CO_2_@Ti^+IV^(pz^Me2^)_4_@SBA-15_500_ (**CO_2_@H3-Ti^+IV^**)

A Schlenk tube was loaded with solid **H3-Ti** ^**+IV**^ (66.4 mg), and the atmosphere was changed to 1 bar CO_2_. After 3 h, **CO**_**2**_ **@H3-Ti** ^**+IV**^ could be collected as a pale-yellow powder (74.7 mg). ^1^H CP/MAS spectrum (300.13 MHz, MAS at 8 kHz): δ = 6.67 (4-*H*(pz)), 5.34 (4-*H*(pz)), 1.83 (C*H*_3_), 0.84 (C*H*_3_) ppm. ^13^C CP/MAS spectrum (75.47 MHz, MAS at 8 kHz): δ = 149.9 (3-*C*(pz)), 142.7 (5-*C*(pz)), 108.1 (4-*C*(pz)), 10.6 (*C*H_3_) ppm. DRIFTS: ṽ = 3390 (vw), 2995 (vw), 2934 (vw), 2864 (vw), 1744 (w), 1573 (vw), 1526 (vw), 1467 (vw), 1419 (vw), 1381 (vw), 1350 (vw), 1068 (vs), 804 (w), 580 (vw), 456 (w) cm^–1^. Elemental analysis found: C 16.39, H 1.97, N 6.77.

### Ti^+IV^(pz^Me2^)­[OSi­(O*t*Bu)_3_]_3_ (**M1-Ti^+IV^**)

Ti^+IV^(NMe_2_)­[OSi­(O*t*Bu)_3_]_3_ (531 mg, 601 μmol) was dissolved in 5 mL toluene, and a solution of Hpz^Me2^ (57.8 mg, 601 μmol) in 8 mL toluene was added dropwise. The solution changed from yellow to colorless. After stirring for 3 h at ambient temperature, the solvent was removed under reduced pressure, the residue was dissolved in *n*-hexane, and stored at −40 °C. Overnight colorless crystals of **M1-Ti** ^**+IV**^ (90%, 503 mg, 538 μmol) were obtained. ^1^H NMR (400.11 MHz, toluene-*d*_8_, 26 °C): δ = 6.11 (s, 1 H, 4-*H*(pz)), 2.42 (s, 6 H, C*H*_3_(pz)), 1.45 (s, 81 H, OC­(C*H*_3_)_3_) ppm. ^13^C­{^1^H} NMR (125.76 MHz, toluene-*d*_8_, 26 °C): δ = 146.3 (3/5-*C*(pz)), 116.0 (4-*C*(pz)), 72.7 (O*C*(CH_3_)_3_), 32.1 (OC­(*C*H_3_)_3_), 13.2 (*C*H_3_(pz)) ppm. DRIFTS: ṽ = 2975 (vs), 2930 (w), 2872 (vw), 1526 (vw), 1462 (vw), 1389 (vw), 1365 (m), 1242 (w), 1217 (vw), 1192 (m), 1056 (vs), 1028 (m), 916 (m), 831 (vw), 703 (w), 497 (vw), 478 (vw), 430 (vw) cm^–1^. Elemental analysis calcd (%) for C_41_H_88_N_2_O_12_Si_3_Ti (933.28 g/mol): C 52.77, H 9.50, N 3.00; found C 52.88, H 9.20, N 3.04.

### Ti^+IV^(CO_2_·pz^Me2^)­[OSi­(O*t*Bu)_3_]_3_ (M2-Ti^+IV^)

**M1-Ti** ^**+IV**^ (531 mg, 601 μmol) was dissolved in 0.5 mL toluene-*d*_8_ in a J. Young NMR tube. The atmosphere was exchanged to a 1 bar CO_2_ atmosphere. ^1^H NMR (400.11 MHz, toluene-*d*_8_, 26 °C): δ = 5.52 (s, 1 H, 4-*H*(pz)), 2.75 (s, 3 H, 5-C*H*_3_(pz)), 2.30 (s, 5 H, 5-C*H*_3_(pz)), 1.46 (OC­(C*H*_3_)_3_) ppm. ^13^C­{^1^H} NMR (100.61 MHz, toluene-*d*_8_, 26 °C): δ = 151.9 (5-*C*(pz)), 149.4 (*C*O_2_), 142.7 (3-*C*(pz)), 110.7 (4-*C*(pz)), 72.2 (O*C*(CH_3_)_3_), 32.1 (OC­(*C*H_3_)_3_), 15.8 (5-*C*H_3_(pz)), 11.9 (3-*C*H_3_(pz)) ppm.

### General Procedure of the Catalytic Conversion of Epoxides to Cyclic Carbonate

Representative example: A small Schlenk tube was charged with 30 mg of [Mg­(pz^*t*Bu2^)_2_]_2_@SBA-15_500_ (≡ 0.55 mg and 22.8 μmol of magnesium) and TBAI (8.4 mg, 22.8 μmol), and the corresponding epoxide (4.567 mmol) was added. The atmosphere was exchanged with 1 bar of CO_2_, and the reaction mixture was stirred for 24 h. The state of conversion from epoxide to cyclic carbonate was determined via ^1^H NMR by dissolving the reaction mixture in CDCl_3_ and filtering off the insoluble material.

## Supplementary Material



## Notes

Solid-state NMR spectra, solution NMR, N_2_ physisorption isotherms, DRIFT spectra, X-ray crystallographic data, as well as ^1^H NMR spectra of the catalytic conversions (PDF)
