# Supplementary material for: Light Metal Pyrazolates Grafted onto Periodic Mesoporous Silica for Carbon Dioxide Capture and Transformation
Source: Inorg Chem. 2026 Feb 3;65(6):3596–605. doi: 10.1021/acs.inorgchem.5c05578 (PMC12914636; doi:10.1021/acs.inorgchem.5c05578)
Supplement: Supplementary file 1 [file ic5c05578_si_001.pdf]

Supporting Information

Light Metal Pyrazolates Grafted onto Periodic Mesoporous Silica for Carbon Dioxide Capture and Transformation

Felix Kracht, Jitpisut Poolwong, Natascha Roth, Yucang Liang, Cäcilia Maichle-Mössmer, and Reiner Anwander*

Institut für Anorganische Chemie, Eberhard Karls Universität Tübingen, Auf der Morgenstelle 18, 72076 Tübingen (Germany)

* to whom correspondence should be addressed: E-Mail reiner.anwander@uni-tuebingen.de

Table of Contents

Solid-state NMR spectra	S3
Solution NMR spectra	S11
Solution NMR spectra catalysis	S14
Leaching test	S21
IR spectra	S23
Crystallographic Data	S30
Proposed Catalytic Scenario	S31
References	S32

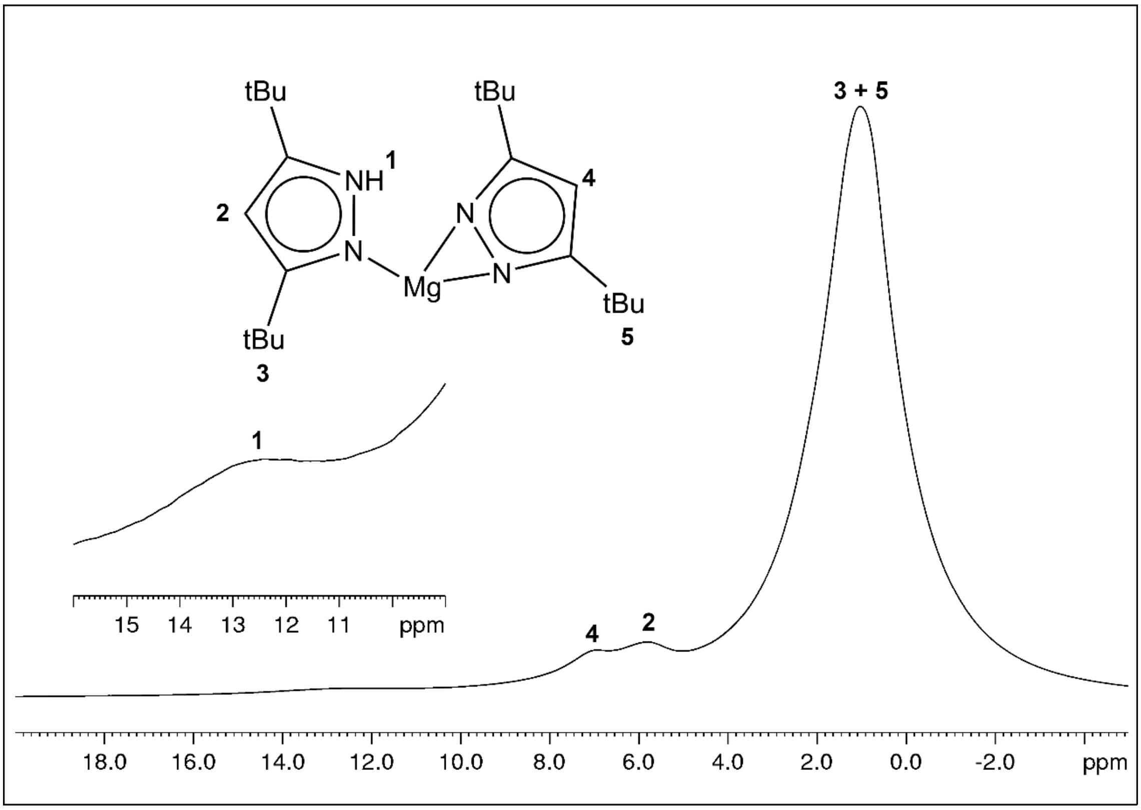

Figure S1. ^{1}H MAS NMR spectrum (300.13 MHz, MAS at 8 kHz) of $[Mg(pz^{tBu_2})_2]_2$@SBA-15_{500} (**H1-Mg**).

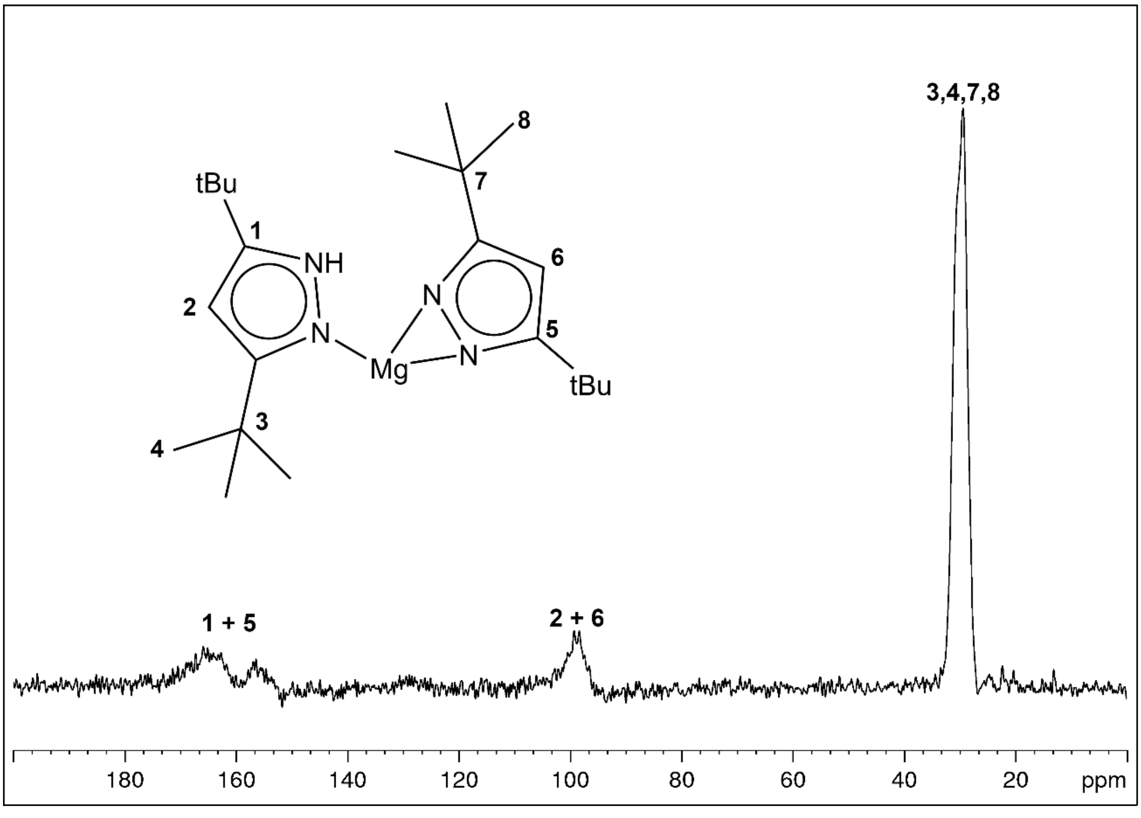

Figure S2. ^{13}C CP/MAS NMR spectrum (75.47 MHz, MAS at 8 kHz) of $[Mg(pz^{tBu_2})_2]_2$@SBA-15_{500} (**H1-Mg**).

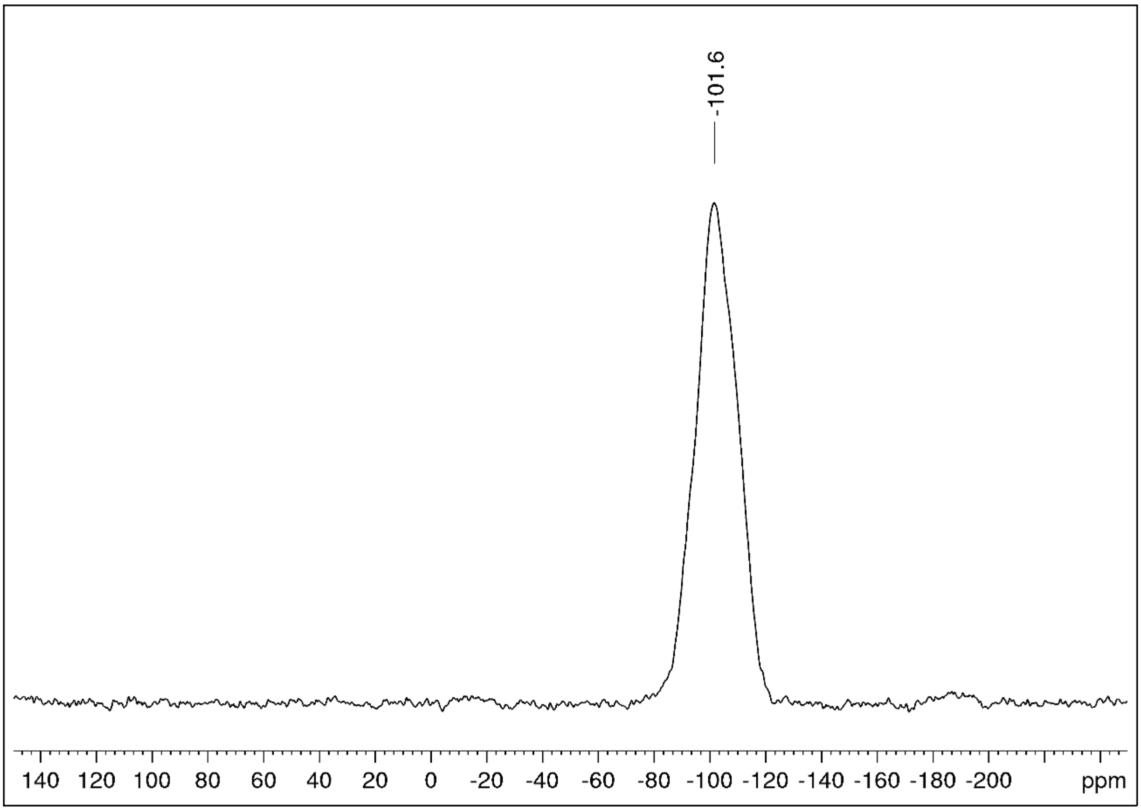

Figure S3. ^{29}Si CP/MAS NMR spectrum (59.63 MHz, MAS at 5 kHz) of [Mg(pz^{tBu_2})$_2$]$_2$@SBA-15$_{500}$ (**H1-Mg**).

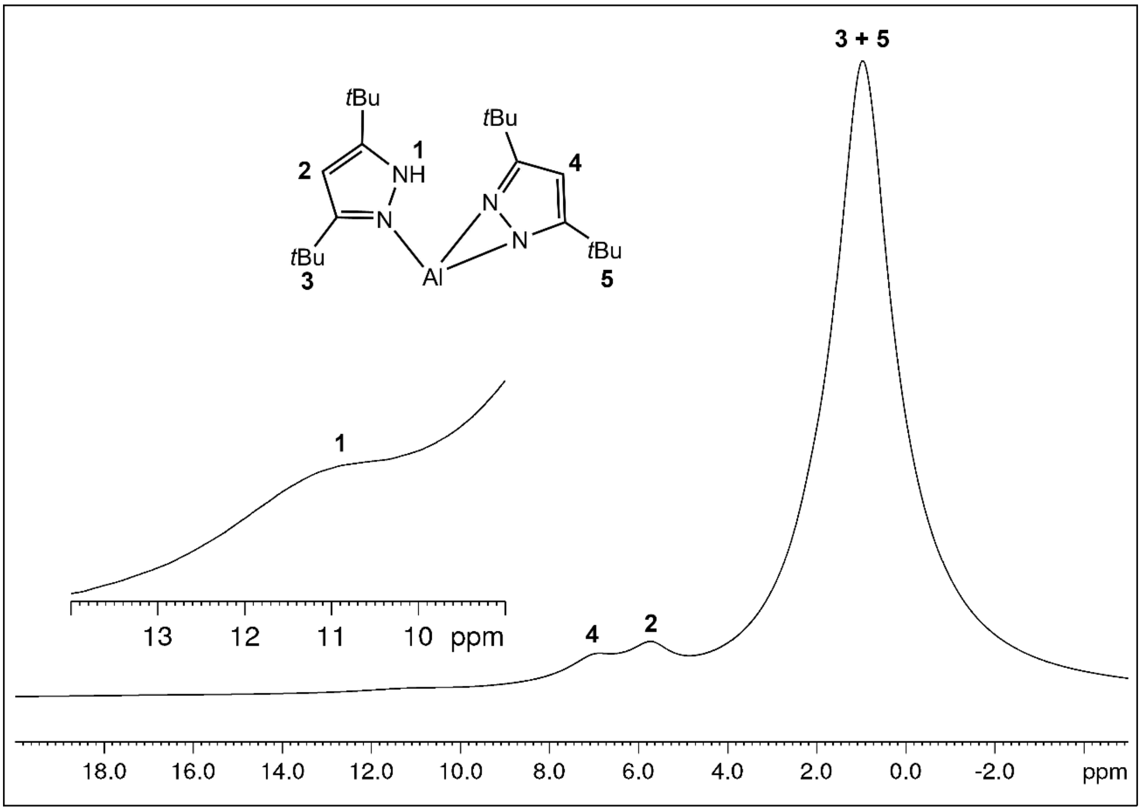

Figure S4. ^{1}H MAS NMR spectrum (300.13 MHz, MAS at 8 kHz) of Al(pz^{tBu_2})$_3$@SBA-15$_{500}$ (**H2-Al**).

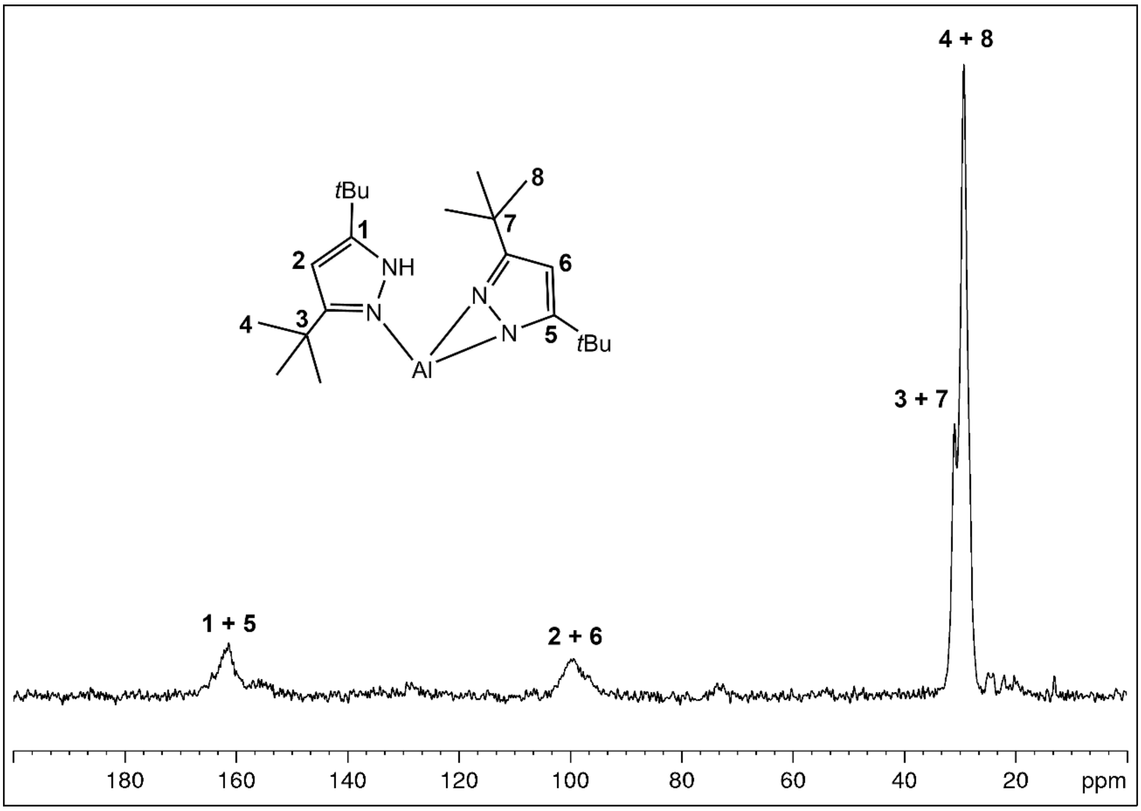

Figure S5. ^{13}C CP/MAS NMR spectrum (75.47 MHz, MAS at 8 kHz) of Al(pz^{tBu_2})$_3$@SBA-15$_{500}$ (**H2-Al**).

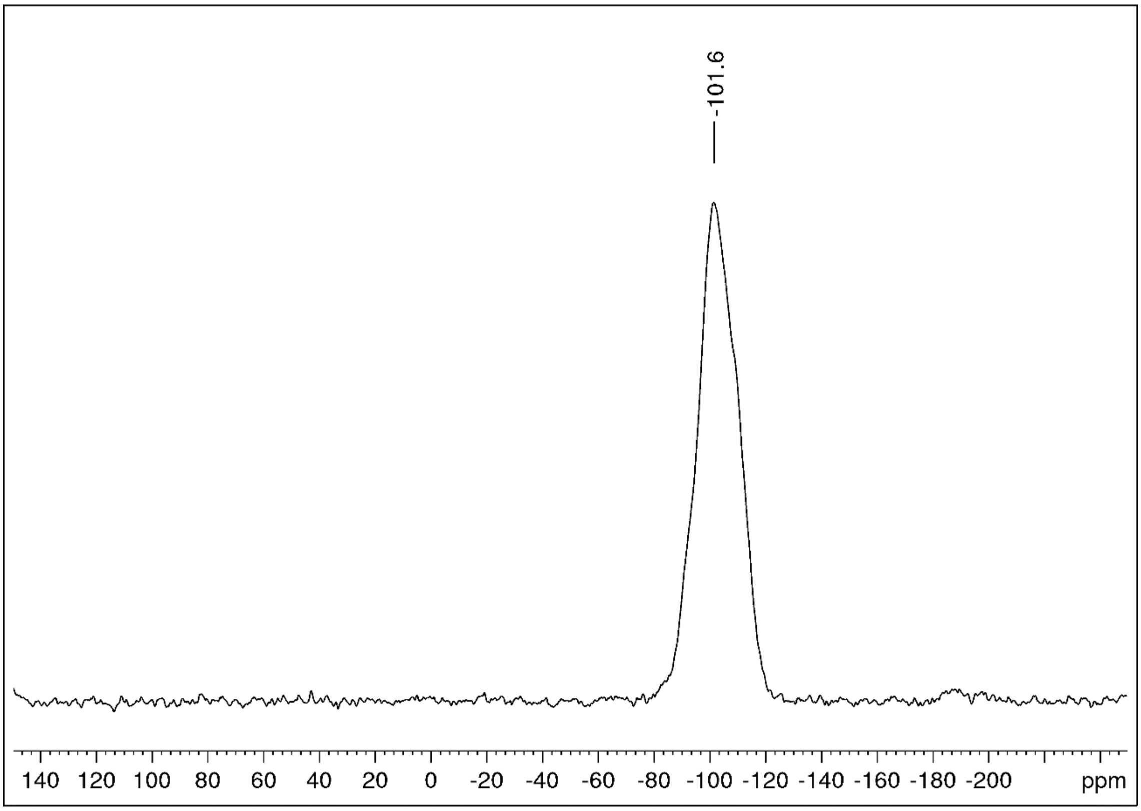

Figure S6. ^{29}Si CP/MAS NMR spectrum (59.63 MHz, MAS at 5 kHz) of Al(pz^{tBu_2})$_3$@SBA-15$_{500}$ (**H2-Al**).

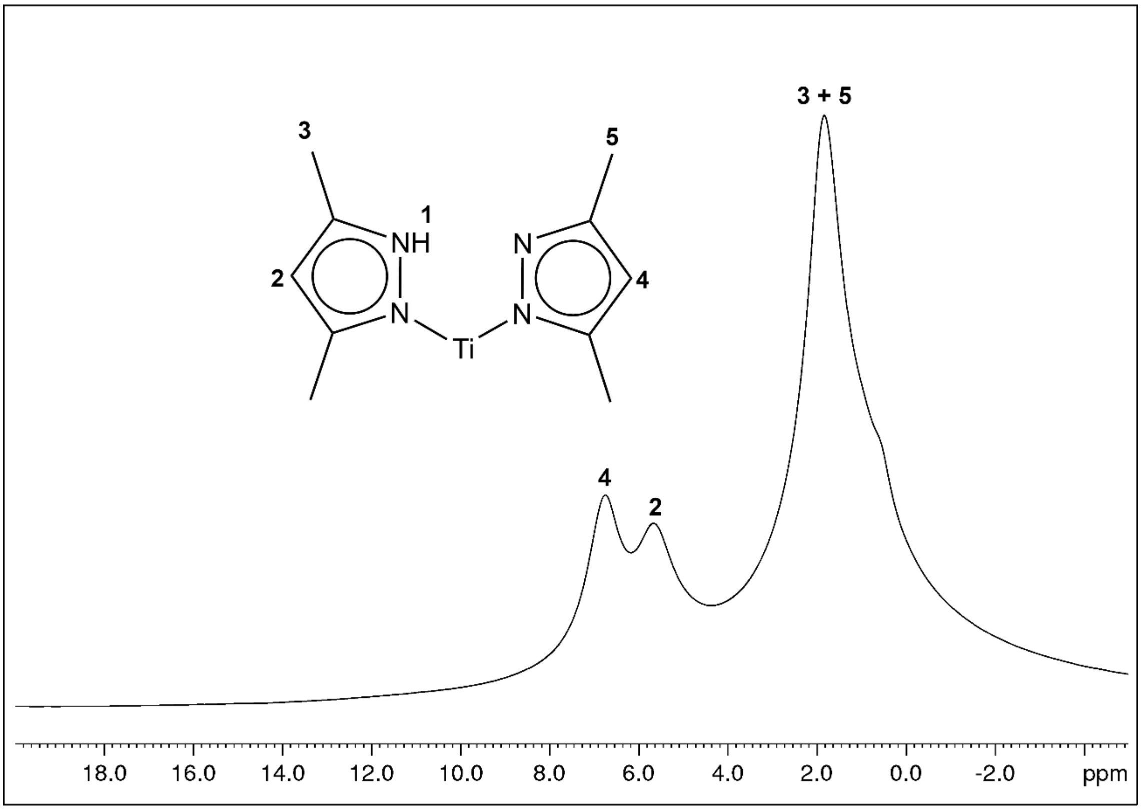

Figure S7. ^{1}H MAS NMR spectrum (300.13 MHz, MAS at 8 kHz) of Ti(pz^{Me2})$_4$@SBA-15$_{500}$ (**H3-Ti^{+IV}**).

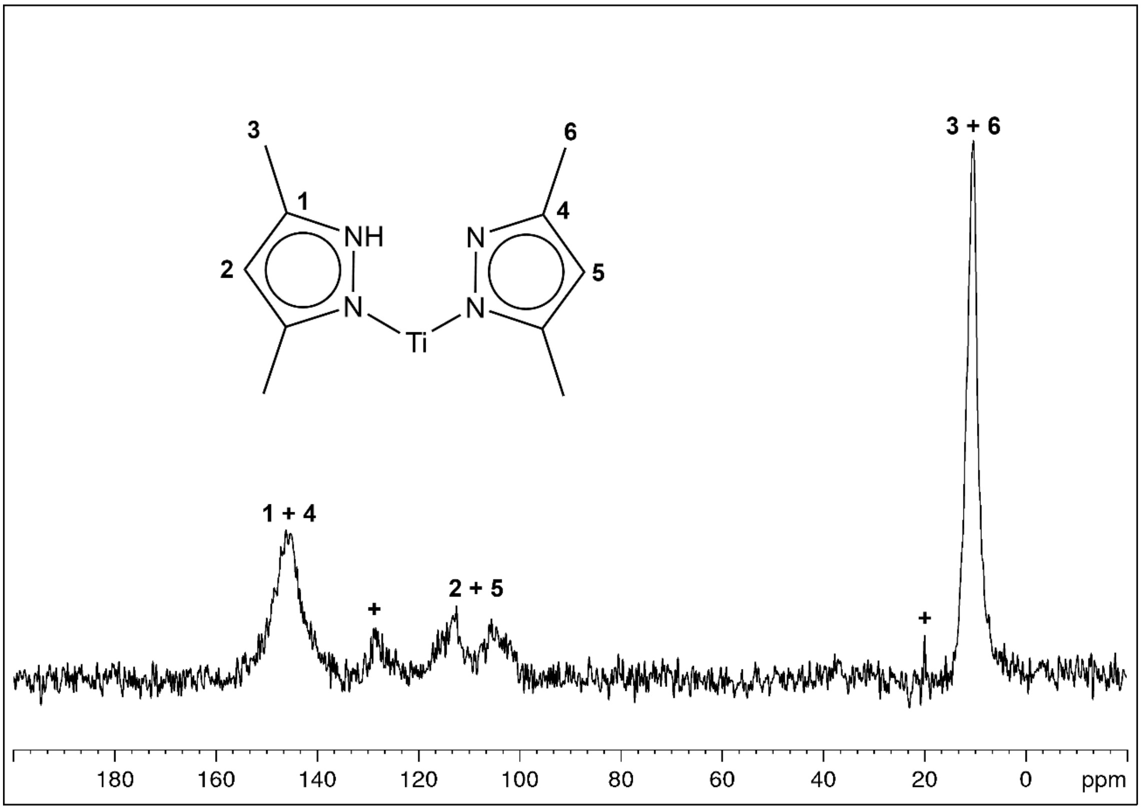

Figure S8. ^{13}C CP/MAS NMR spectrum (75.47 MHz, MAS at 8 kHz) of Ti(pz^{Me2})$_4$@SBA-15$_{500}$ (**H3-Ti^{+IV}**) (+ residual toluene).

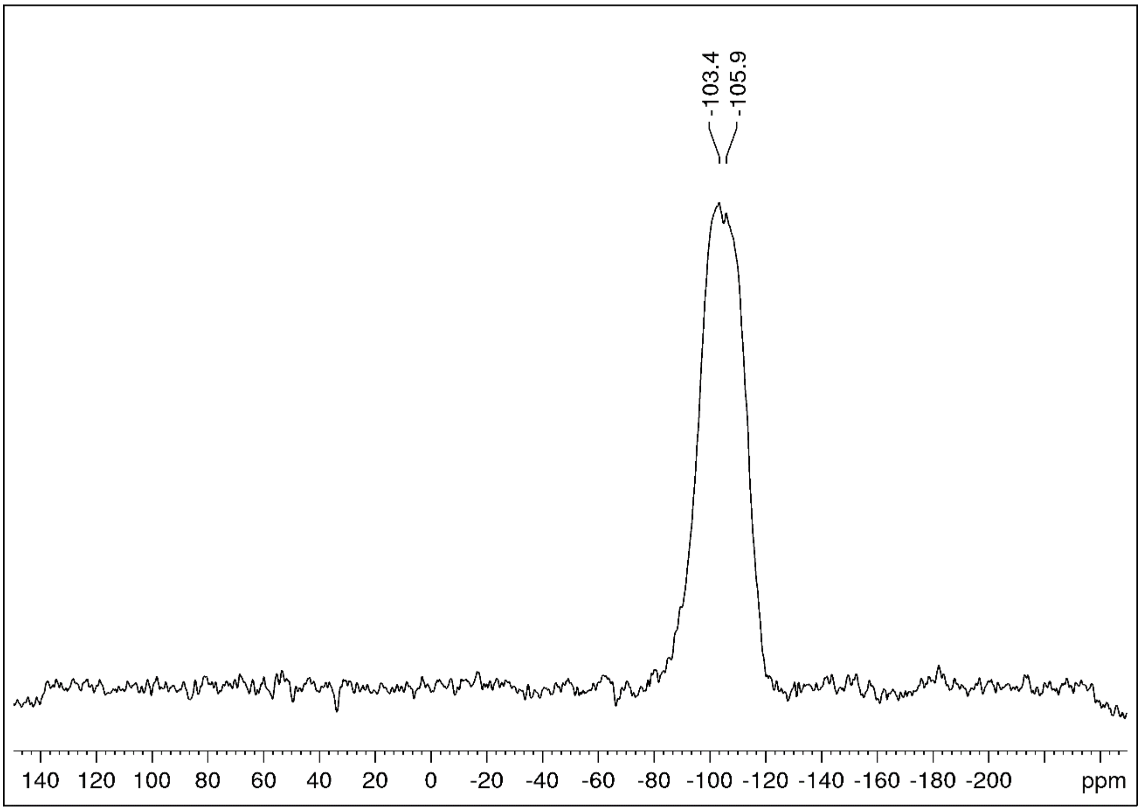

Figure S9. ^{29}Si CP/MAS NMR spectrum (59.63 MHz, MAS at 5 kHz) of Ti(pz^{Me2})$_4$@SBA-15$_{500}$ (**H3-Ti^{+IV}**).

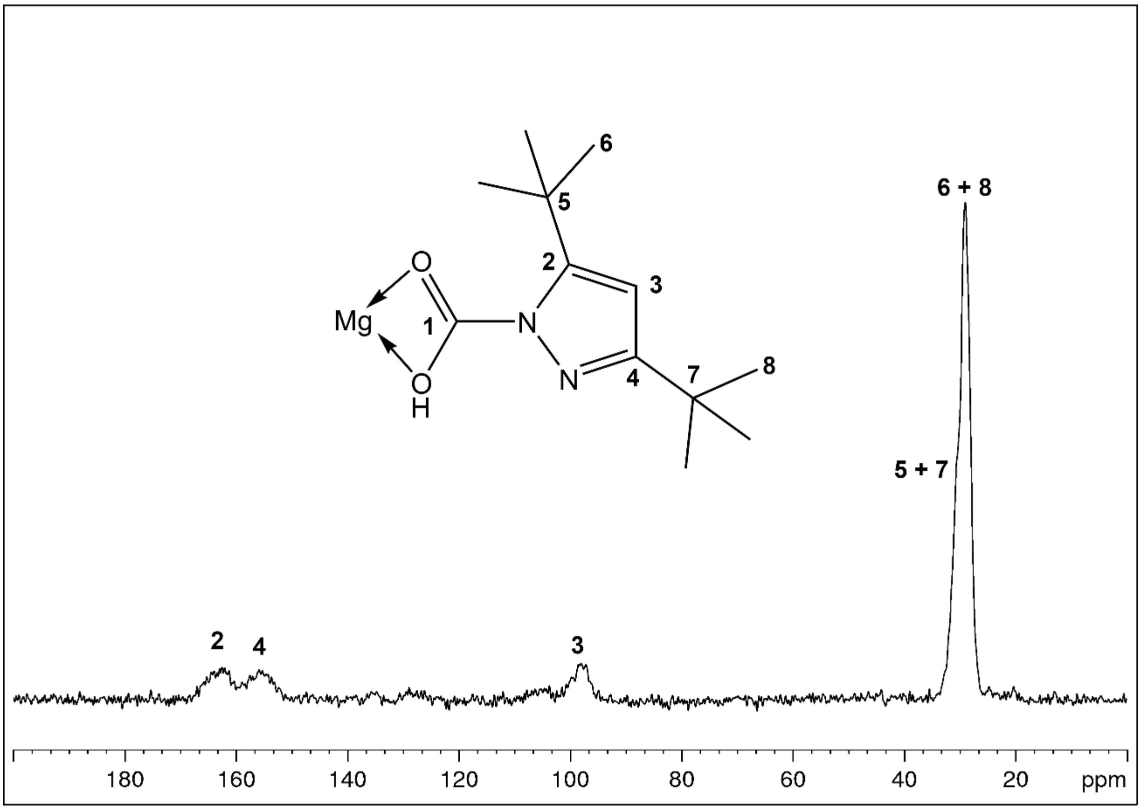

Figure S10. ^{13}C CP/MAS NMR spectrum (75.47 MHz, MAS at 8 kHz) of CO$_2$@[Mg(pz^{tBu2})$_2$]$_2$@SBA-15$_{500}$ (**CO$_2$@H1-Mg**).

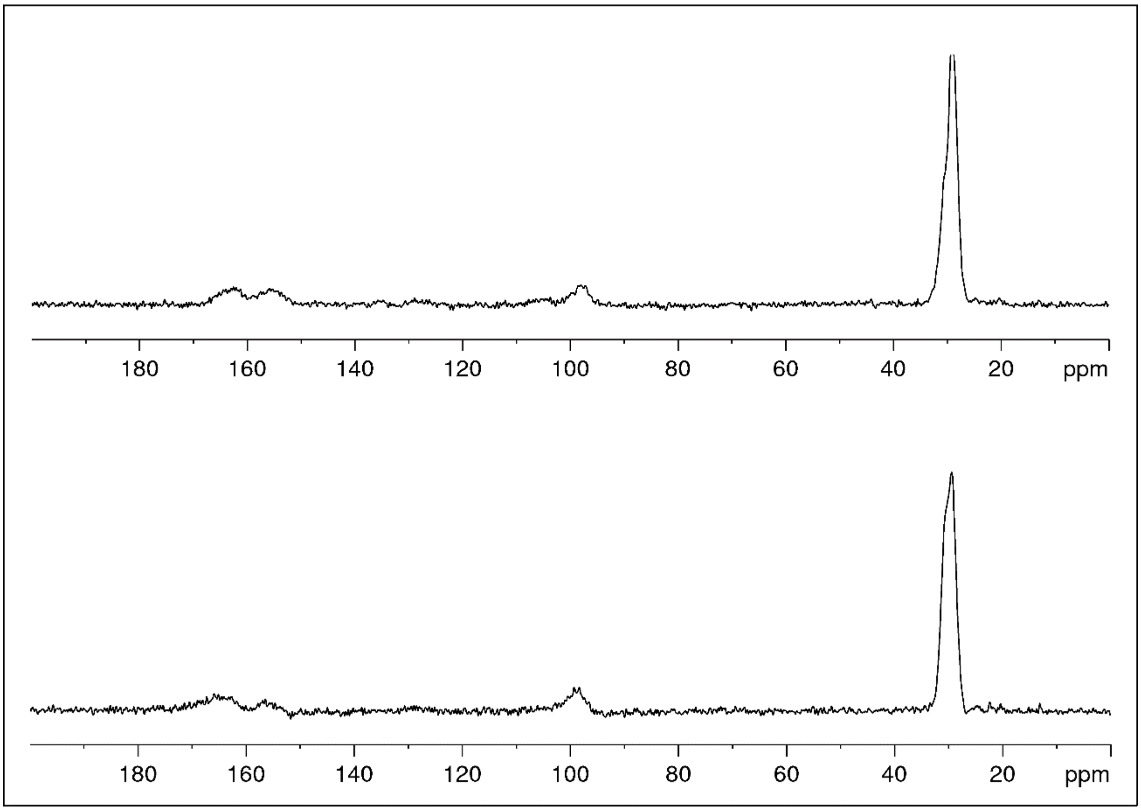

Figure S11. Comparison of ^{13}C CP/MAS NMR spectra (75.47 MHz, MAS at 8 kHz) of $[Mg(pz^{tBu_2})_2]_2$@SBA-15_{500} (**H1-Mg**) and CO_2@$[Mg(pz^{tBu_2})_2]_2$@SBA-15_{500} (**CO_2@H1-Mg**).

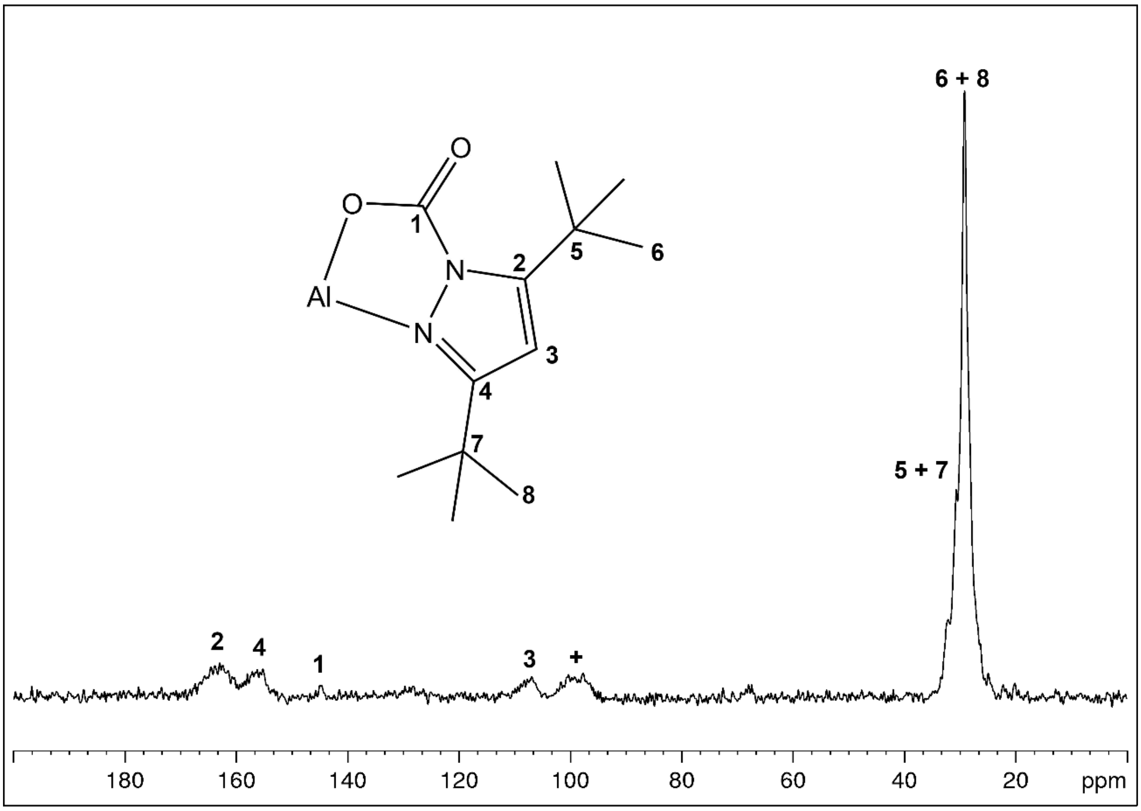

Figure S12. ^{13}C CP/MAS NMR spectrum (75.47 MHz, MAS at 8 kHz) of CO_2@$Al(pz^{tBu_2})_3$@SBA-15_{500} (**CO_2@H2-Al**) (+ carbamic acid HO_2C pz^{tBu_2} coordinated to the surface).

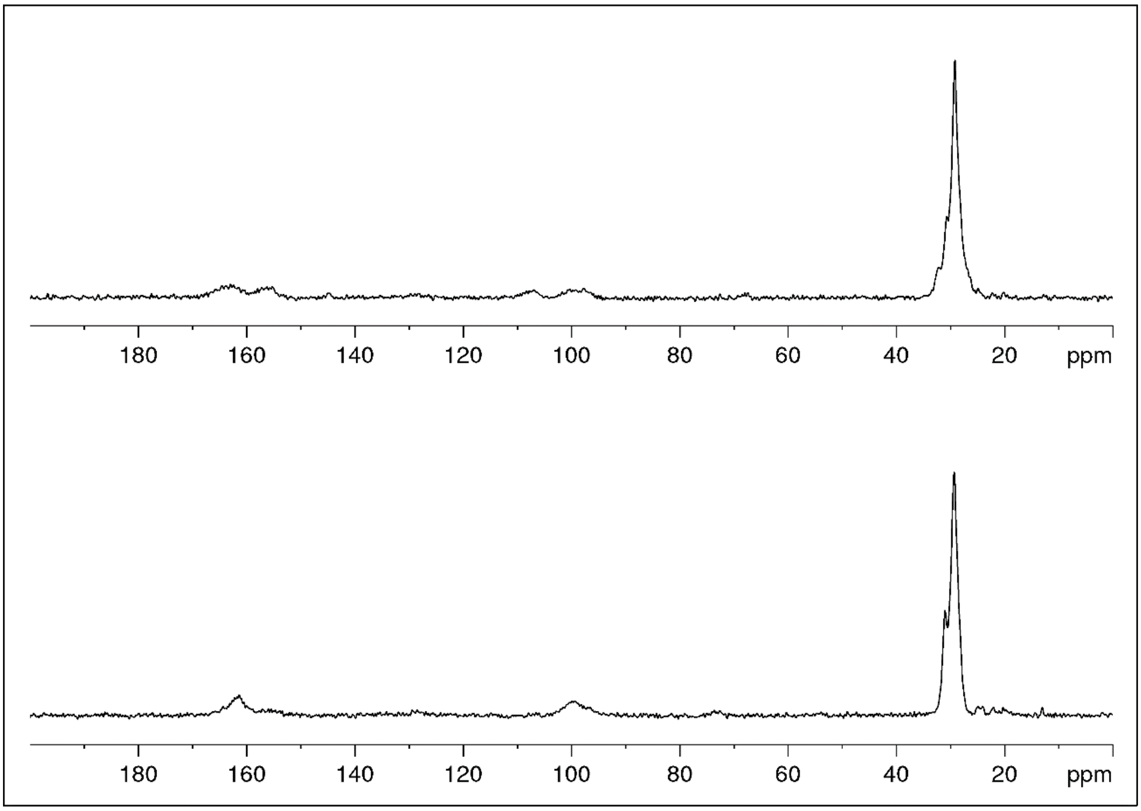

Figure S13. Comparison of ^{13}C CP/MAS NMR spectra (75.47 MHz, MAS at 8 kHz) of Al(pz^{tBu_2})$_3$@SBA-15$_{500}$ (**H2-Al**) and CO_2@Al(pz^{tBu_2})$_3$@SBA-15$_{500}$ (**CO_2@H2-Al**).

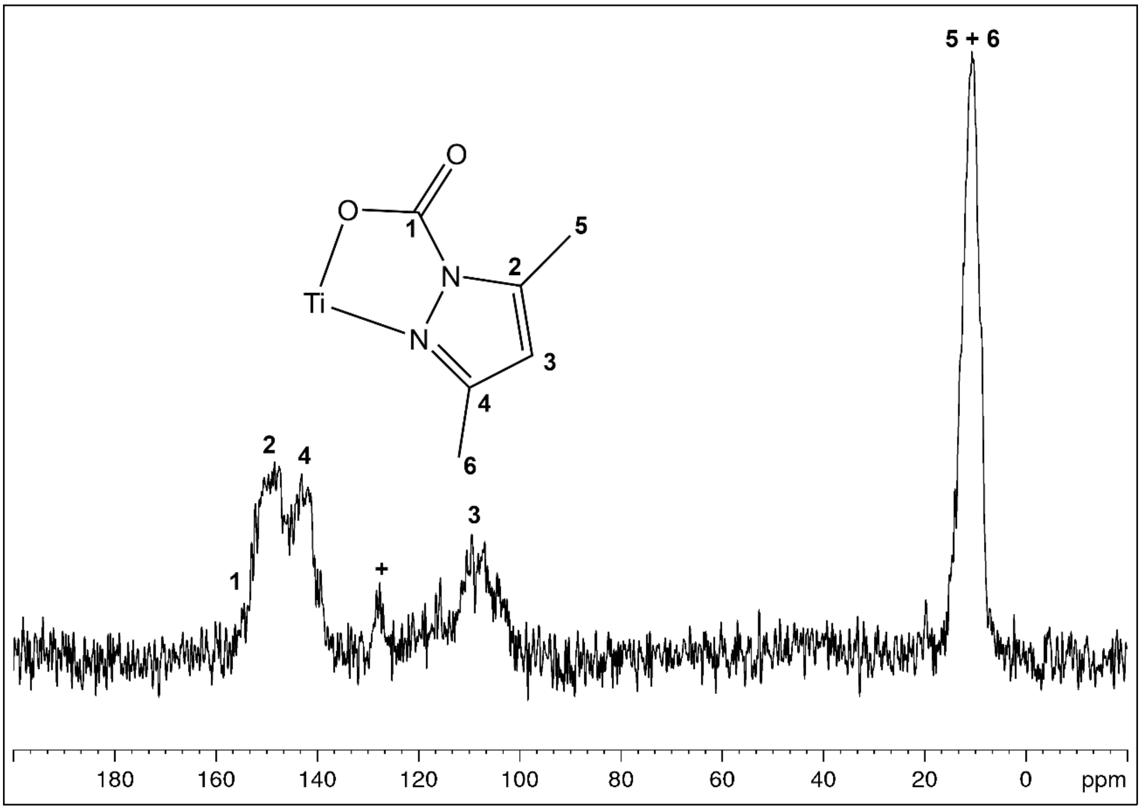

Figure S14. ^{13}C CP/MAS NMR spectrum (75.47 MHz, MAS at 8 kHz) of CO_2@Ti(pz^{Me_2})$_4$@SBA-15$_{500}$ (**CO_2@H3-Ti^{+IV}**) (+ residual toluene).

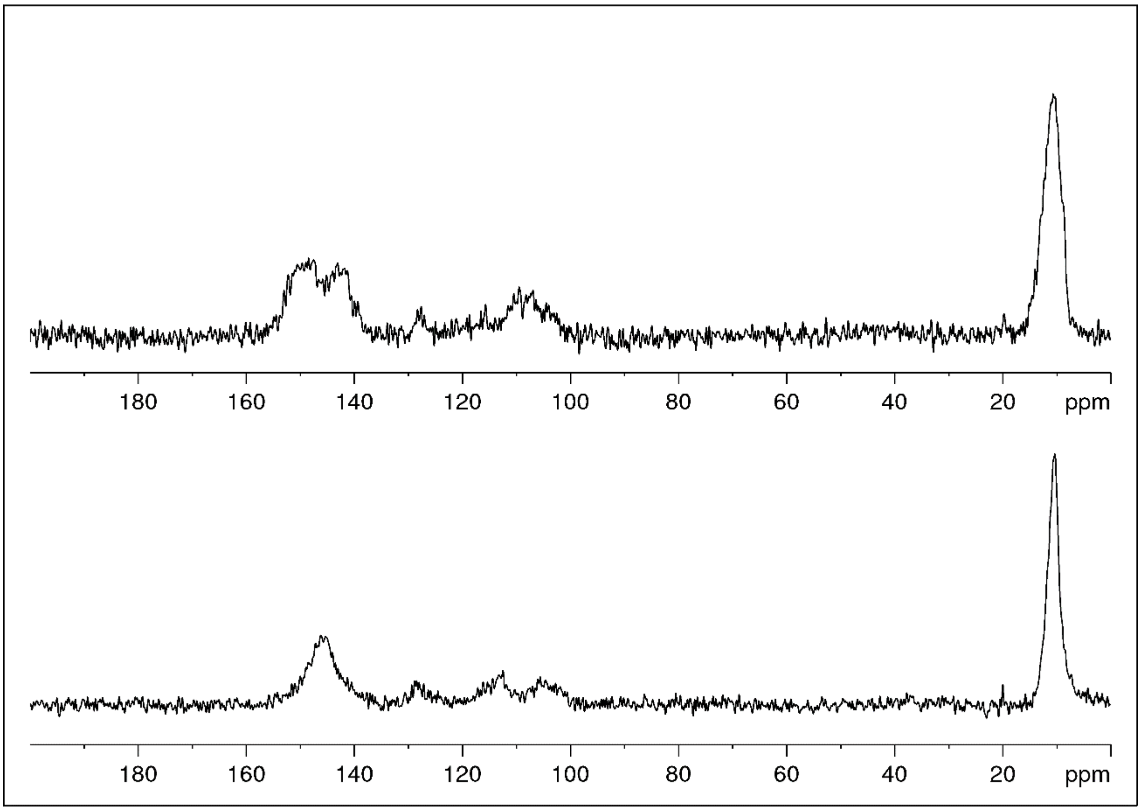

Figure S15. Comparison of ^{13}C CP/MAS NMR spectra (75.47 MHz, MAS at 8 kHz) of Ti(pz^{Me_2})$_4$@SBA-15$_{500}$ (**H3-Ti^{+IV}**) and CO_2@Ti(pz^{Me_2})$_4$@SBA-15$_{500}$ (**CO_2@H3-Ti^{+IV}**).

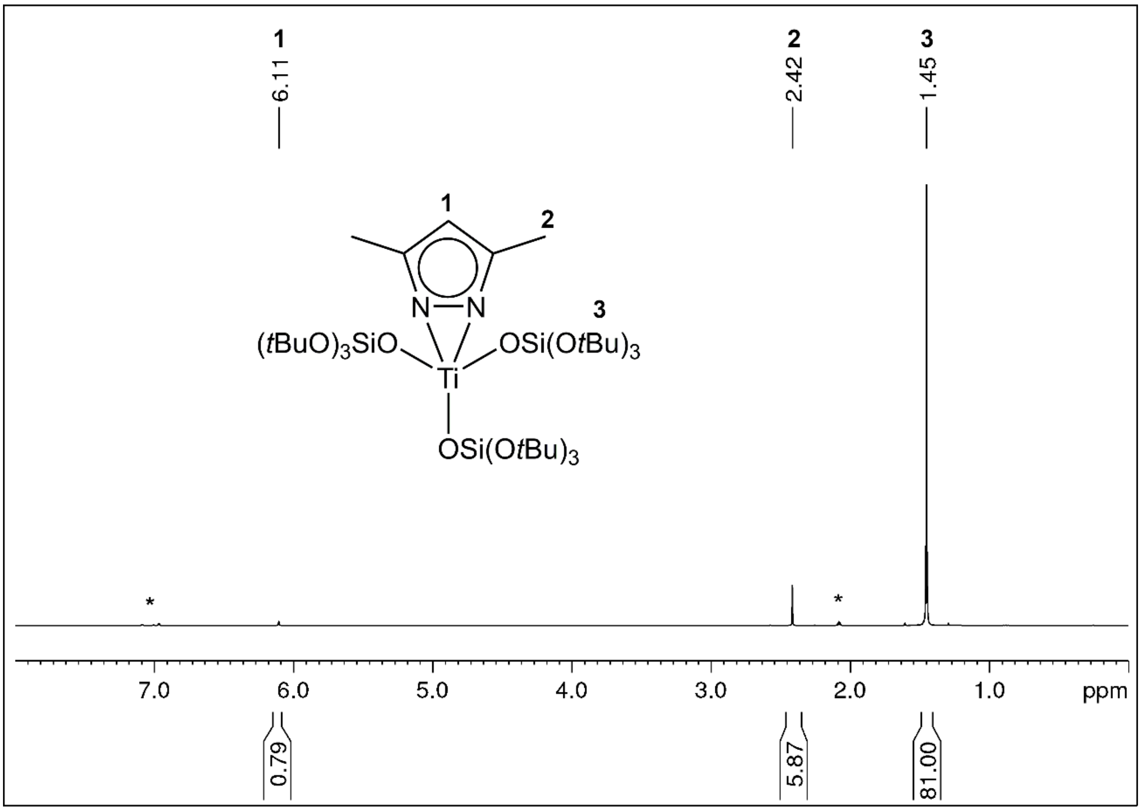

Figure S16. ^{1}H NMR spectrum (26 °C, 400.11 MHz, toluene-*d*$_8$) of Ti^{+IV}(pz^{Me2})[OSi(O*t*Bu)$_3$]$_3$ (**M1-Ti^{+IV}**).

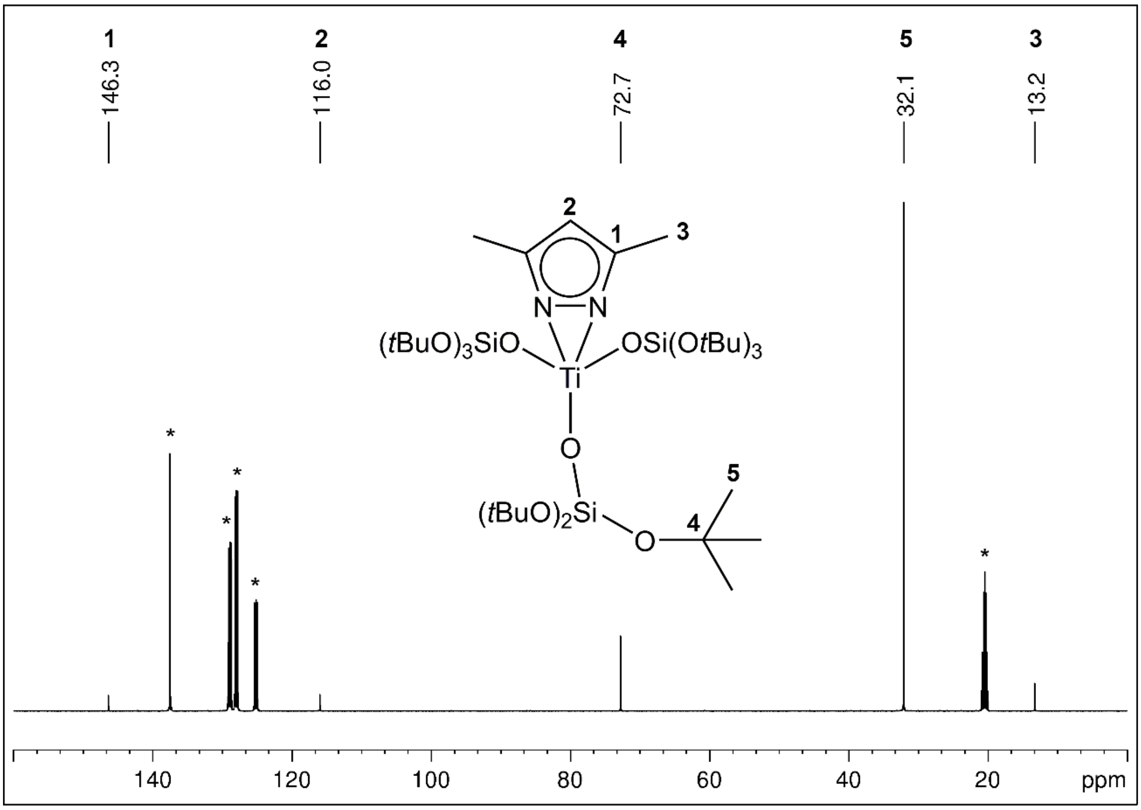

Figure S17. ^{13}C{^{1}H} NMR spectrum (26 °C, 125.76 MHz, toluene-*d*$_8$) of Ti^{+IV}(pz^{Me2})[OSi(O*t*Bu)$_3$]$_3$ (**M1-Ti^{+IV}**).

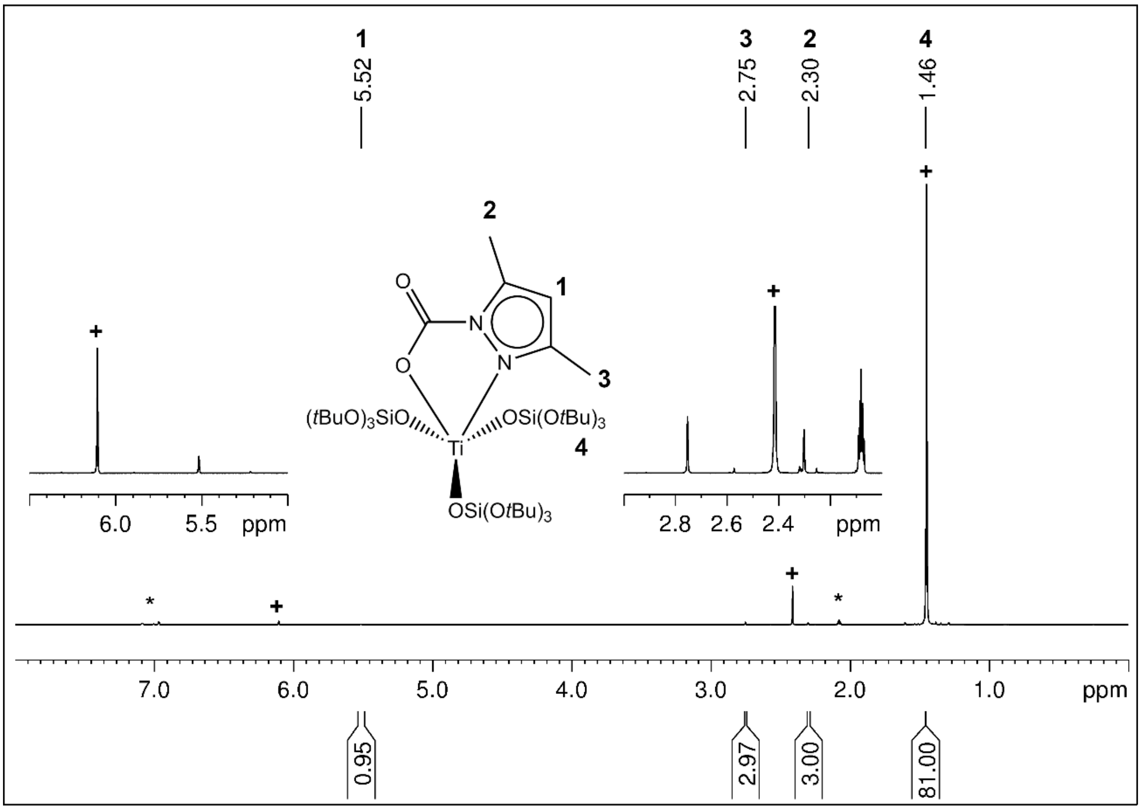

Figure S18. ^{1}H NMR spectrum (26 °C, 400.11 MHz, toluene-*d*$_8$)) of $Ti^{+IV}(CO_2{\cdot}pz^{Me2})[OSi(OtBu)_3]_3$ (**M2-Ti^{+IV}**) (+ starting material: $Ti^{+IV}(pz^{Me2})[OSi(OtBu)_3]_3$ (**M1-Ti^{+IV}**)).

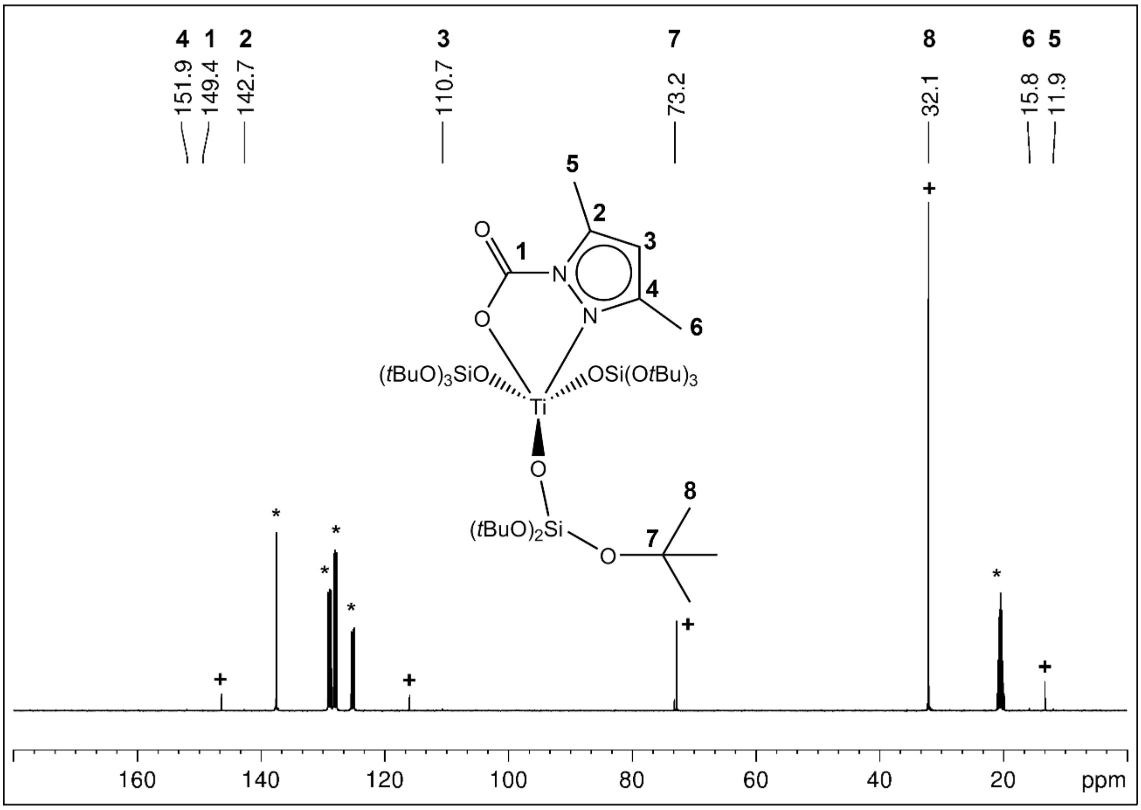

Figure S19. ^{13}C{^{1}H} NMR spectrum (26 °C, 100.61 MHz, toluene-*d*$_8$) of) of $Ti^{+IV}(CO_2{\cdot}pz^{Me2})[OSi(OtBu)_3]_3$ (**M2-Ti^{+IV}**) (+ starting material: $Ti^{+IV}(pz^{Me2})[OSi(OtBu)_3]_3$ (**M1-Ti^{+IV}**)).

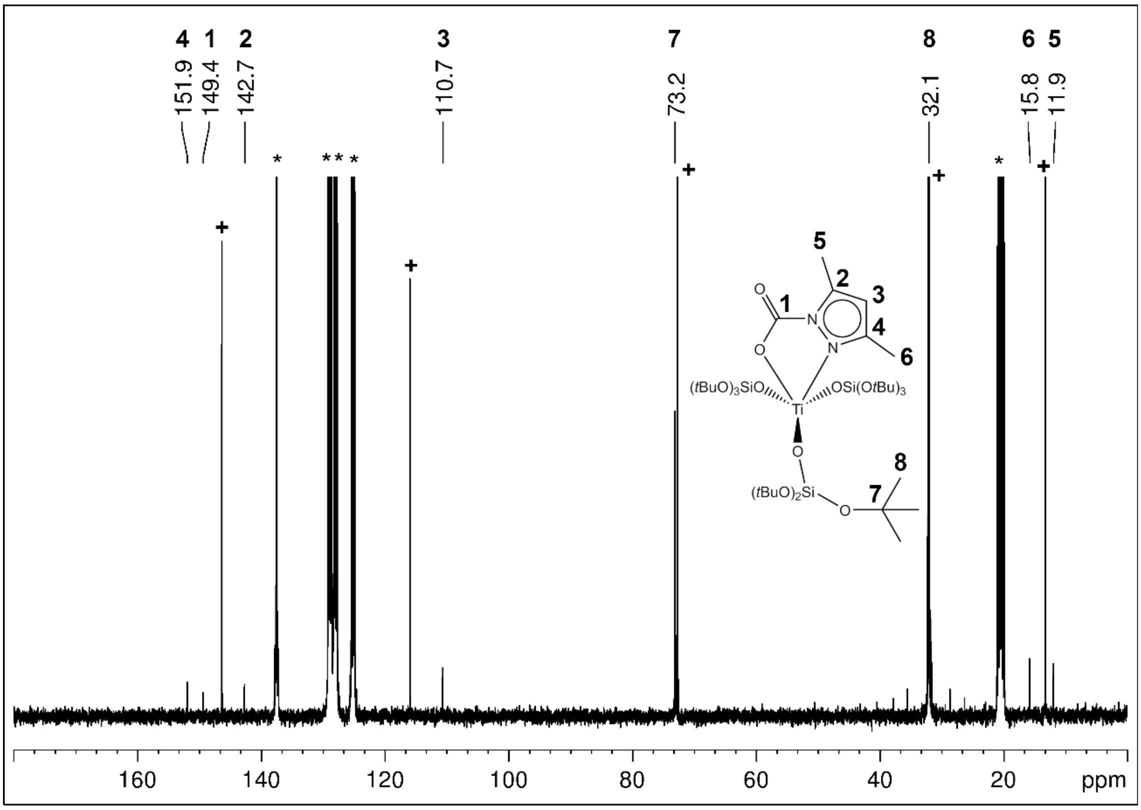

Figure S20. Zoomed in $^{13}C\{^{1}H\}$ NMR spectrum (26 °C, 100.61 MHz, toluene-d_8) of $Ti^{+IV}(CO_2{\cdot}pz^{Me2})[OSi(O\mathit{t}Bu)_3]_3$ (**M2-Ti^{+IV}**) (+ starting material: $Ti^{+IV}(pz^{Me2})[OSi(O\mathit{t}Bu)_3]_3$ (**M1-Ti^{+IV}**)).

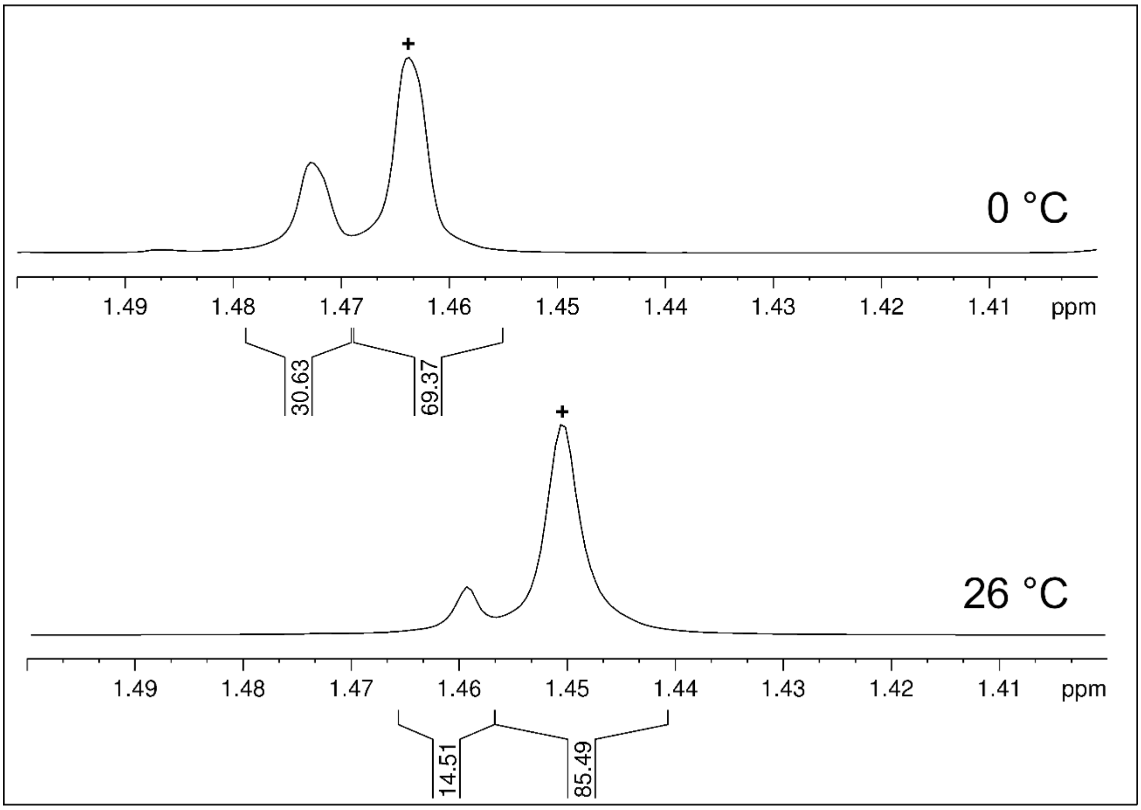

Figure S21. CO_2 uptake of $Ti^{+IV}(pz^{Me2})[OSi(O\mathit{t}Bu)_3]_3$ (**M1-Ti^{+IV}**, marked with +) to $Ti^{+IV}(CO_2{\cdot}pz^{Me2})[OSi(O\mathit{t}Bu)_3]_3$ (**M2-Ti^{+IV}**) in dependence of the temperature. Comparison of the ^{1}H NMR spectra at 26 °C (400.11 MHz, Tol-d_8) and 0 °C (500.11 MHz, toluene-d_8).

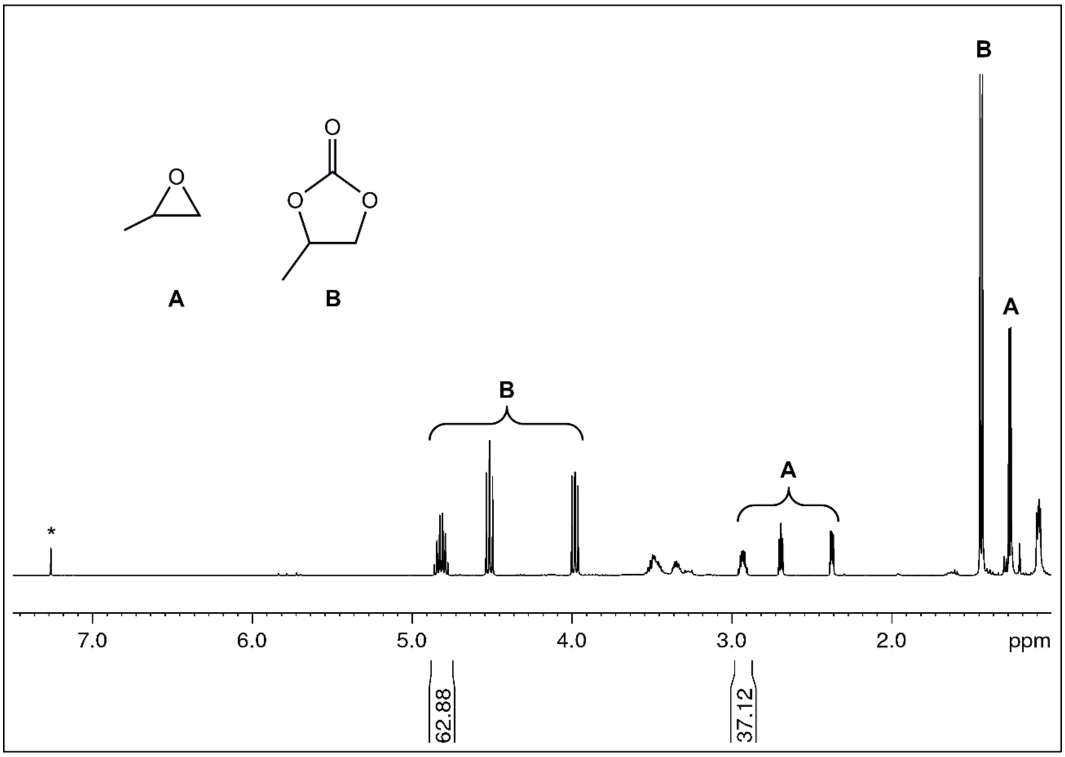

Figure S22. ^{1}H NMR spectrum (26 °C, 400.11 MHz, chloroform-*d*) of the reaction mixture of the catalytic conversion of propylene oxide and CO_2 to propylene carbonate by using 0.5 mol% of $[Mg(pz^{tBu_2})_2]_2$@SBA-15_{500} (**H1-Mg**) as catalyst and TBAB as cocatalyst.

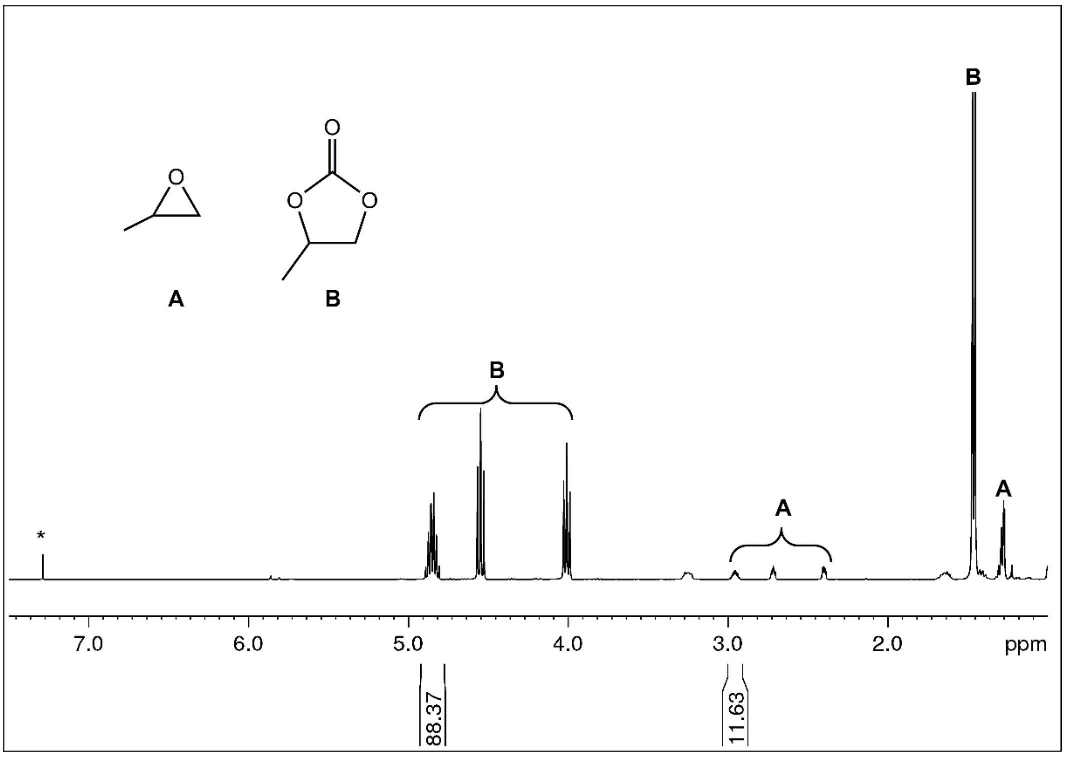

Figure S23. ^{1}H NMR spectrum (26 °C, 400.11 MHz, chloroform-*d*) of the reaction mixture of the catalytic conversion of propylene oxide and CO_2 to propylene carbonate by using 0.5 mol% of $[Mg(pz^{tBu_2})_2]_2$@SBA-15_{500} (**H1-Mg**) as catalyst and TBAI as cocatalyst.

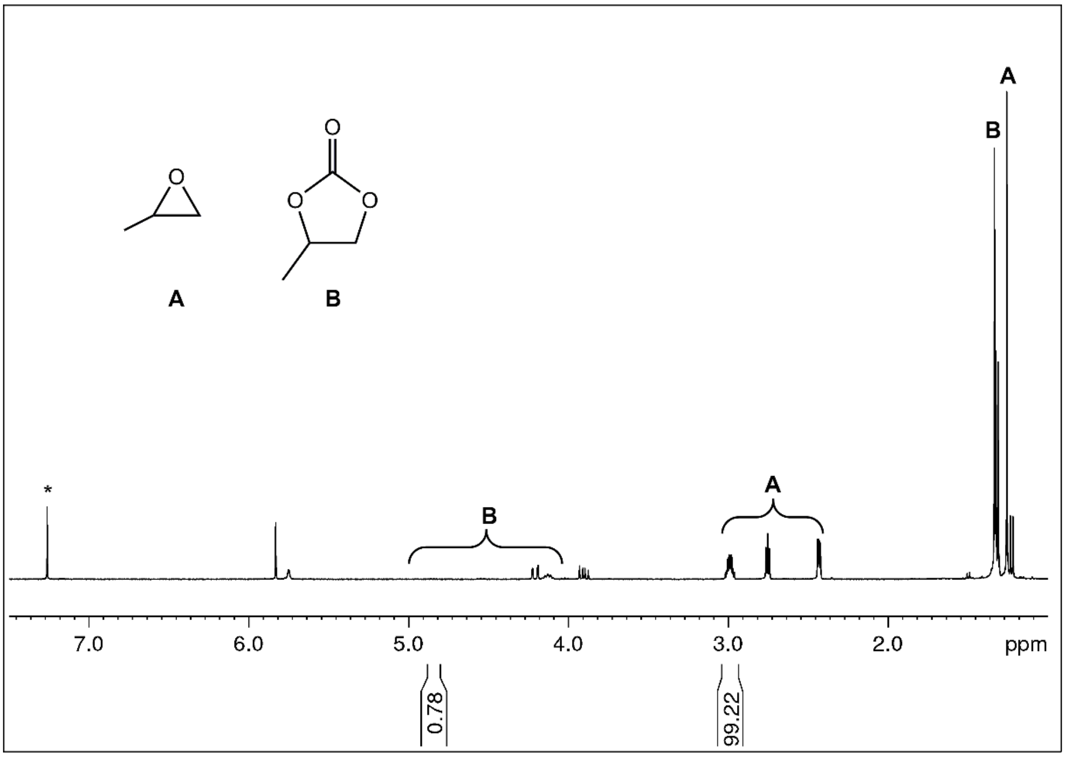

Figure S24. ^{1}H NMR spectrum (26 °C, 400.11 MHz, chloroform-*d*) of the reaction mixture of the catalytic conversion of propylene oxide and CO_2 to propylene carbonate by using 0.5 mol% of [Mg(pz^{tBu_2})$_2$]$_2$@SBA-15$_{500}$ (**H1-Mg**) as catalyst and without cocatalyst.

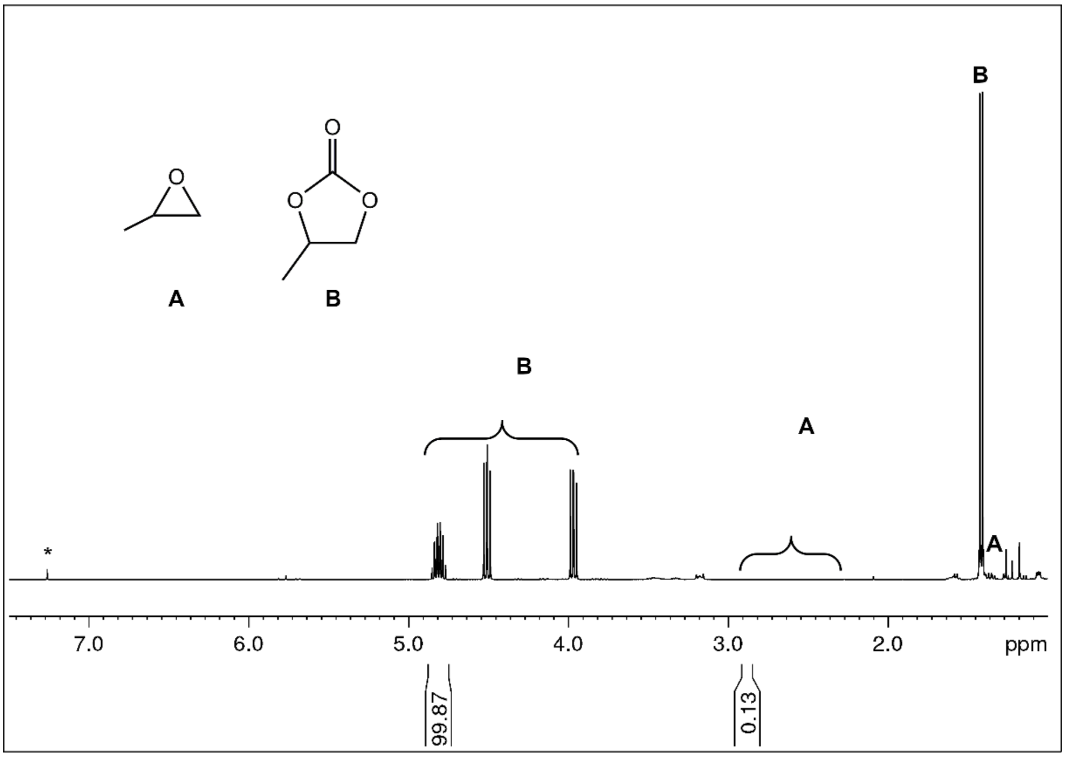

Figure S25. ^{1}H NMR spectrum (26 °C, 400.11 MHz, chloroform-*d*) of the reaction mixture of the catalytic conversion of propylene oxide and CO_2 to propylene carbonate by using 0.5 mol% of [Mg(pz^{tBu_2})$_2$]$_2$@SBA-15$_{500}$ (**H1-Mg**) as catalyst and TBAI as cocatalyst at 90 °C and 10 bar CO_2.

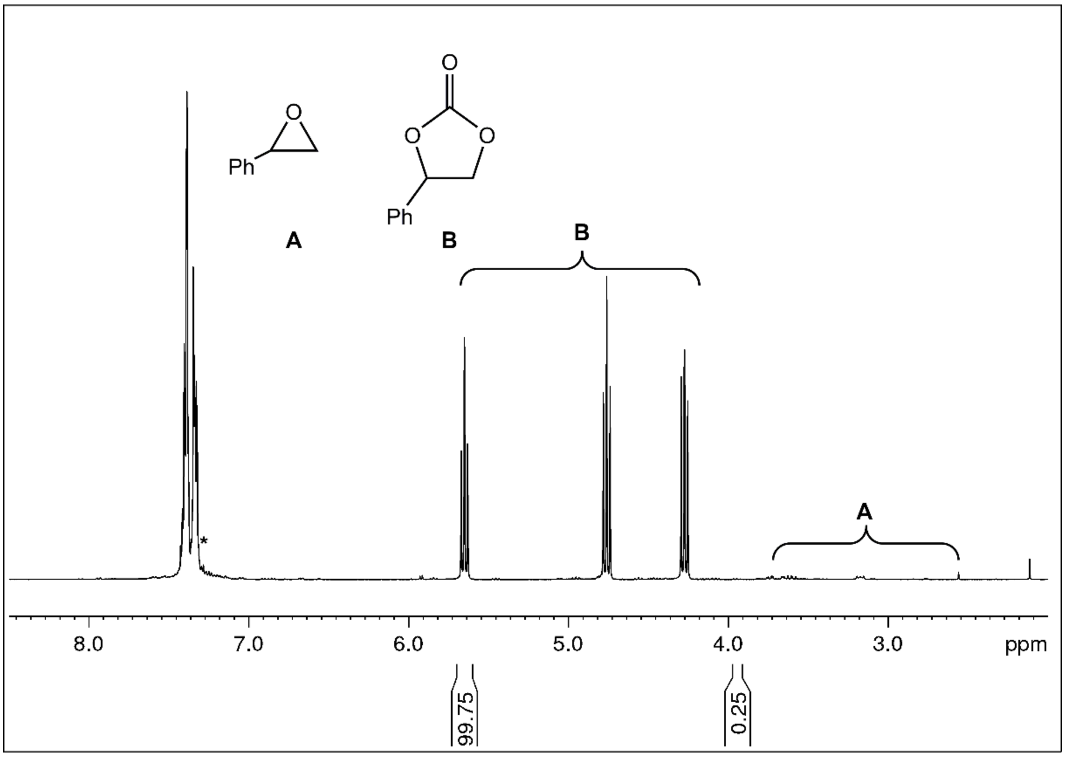

Figure S26. ^{1}H NMR spectrum (26 °C, 400.11 MHz, chloroform-*d*) of the reaction mixture of the catalytic conversion of styrene oxide and CO_2 to styrene carbonate by using 0.5 mol% of $[Mg(pz^{tBu_2})_2]_2$@SBA-15_{500} (**H1-Mg**) as catalyst and TBAI as cocatalyst at 90 °C and 10 bar CO_2.

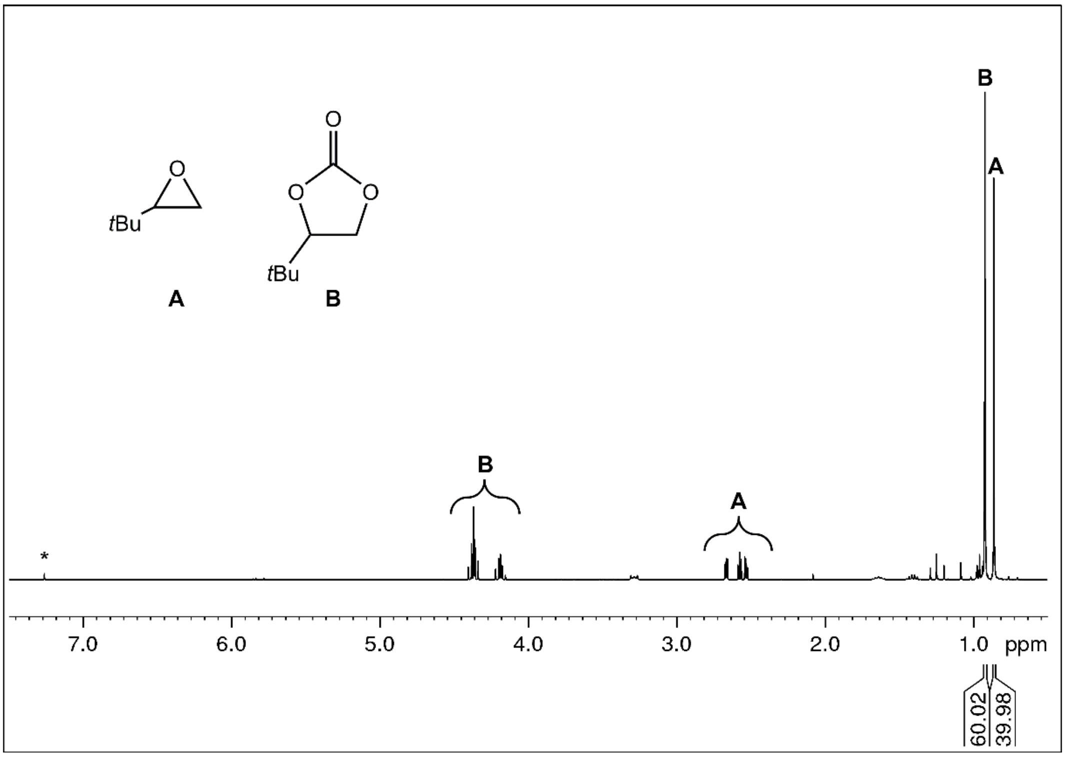

Figure S27. ^{1}H NMR spectrum (26 °C, 400.11 MHz, chloroform-*d*) of the reaction mixture of the catalytic conversion of 2-*tert*-butyloxirane and CO_2 to 3,3-dimethyl-1,2-butene carbonate by using 0.5 mol% of $[Mg(pz^{tBu_2})_2]_2$@SBA-15_{500} (**H1-Mg**) as catalyst and TBAI as cocatalyst at 90 °C and 10 bar CO_2.

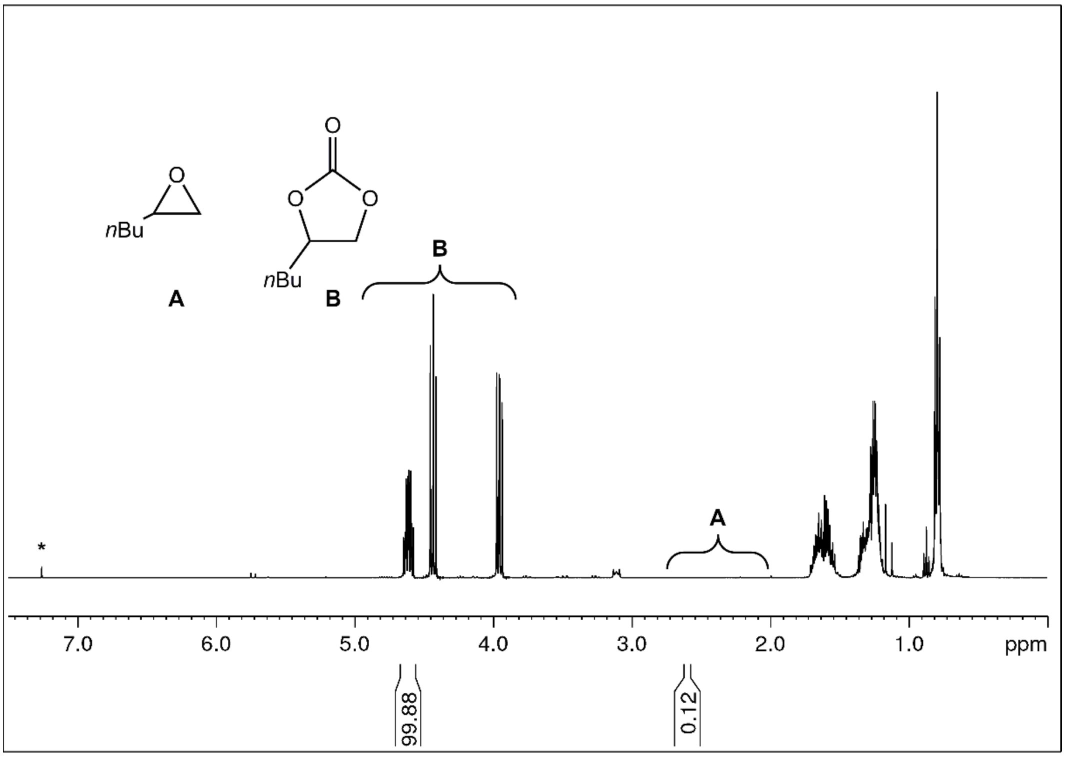

Figure S28. ^{1}H NMR spectrum (26 °C, 400.11 MHz, chloroform-*d*) of the reaction mixture of the catalytic conversion of 1,2-epoxyhexane and CO_2 to 1,2-*n*-hexylene carbonate by using 0.5 mol% of $[Mg(pz^{tBu_2})_2]_2$@SBA-15_{500} (**H1-Mg**) as catalyst and TBAI as cocatalyst at 90 °C and 10 bar CO_2.

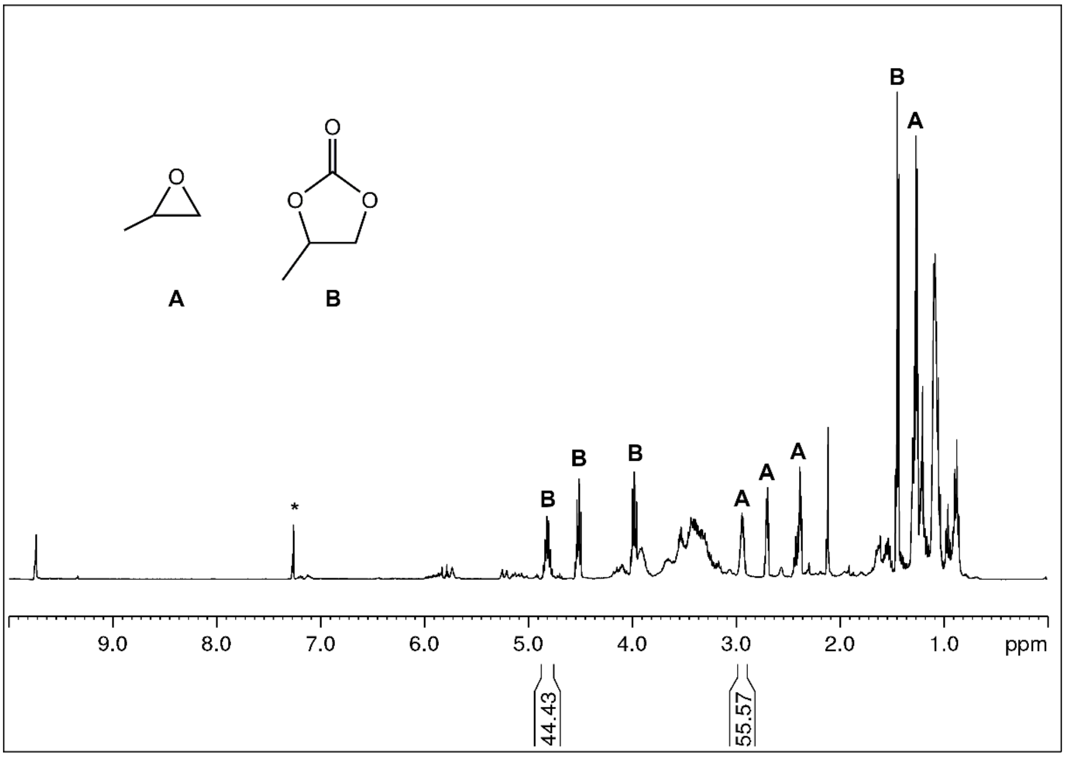

Figure S29. ^{1}H NMR spectrum (26 °C, 400.11 MHz, chloroform-*d*) of the reaction mixture of the catalytic conversion of propylene oxide and CO_2 to propylene carbonate by using 0.5 mol% of $Al(pz^{tBu_2})_3$@SBA-15_{500} (**H2-Al**) as catalyst and TBAB as cocatalyst.

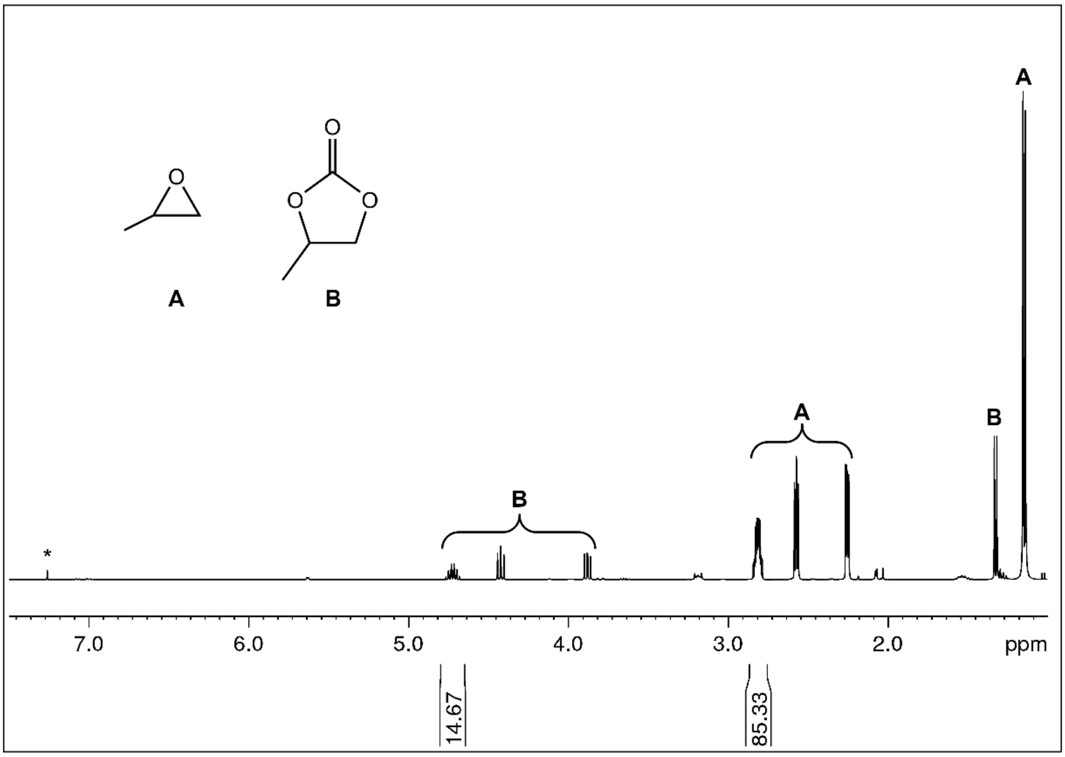

Figure S30. ^{1}H NMR spectrum (26 °C, 400.11 MHz, chloroform-*d*) of the reaction mixture of the catalytic conversion of propylene oxide and CO_2 to propylene carbonate by using 0.5 mol% of Ti(pz^{Me2})$_4$@SBA-15$_{500}$ (**H3-Ti^{+IV}**) as catalyst and TBAB as cocatalyst.

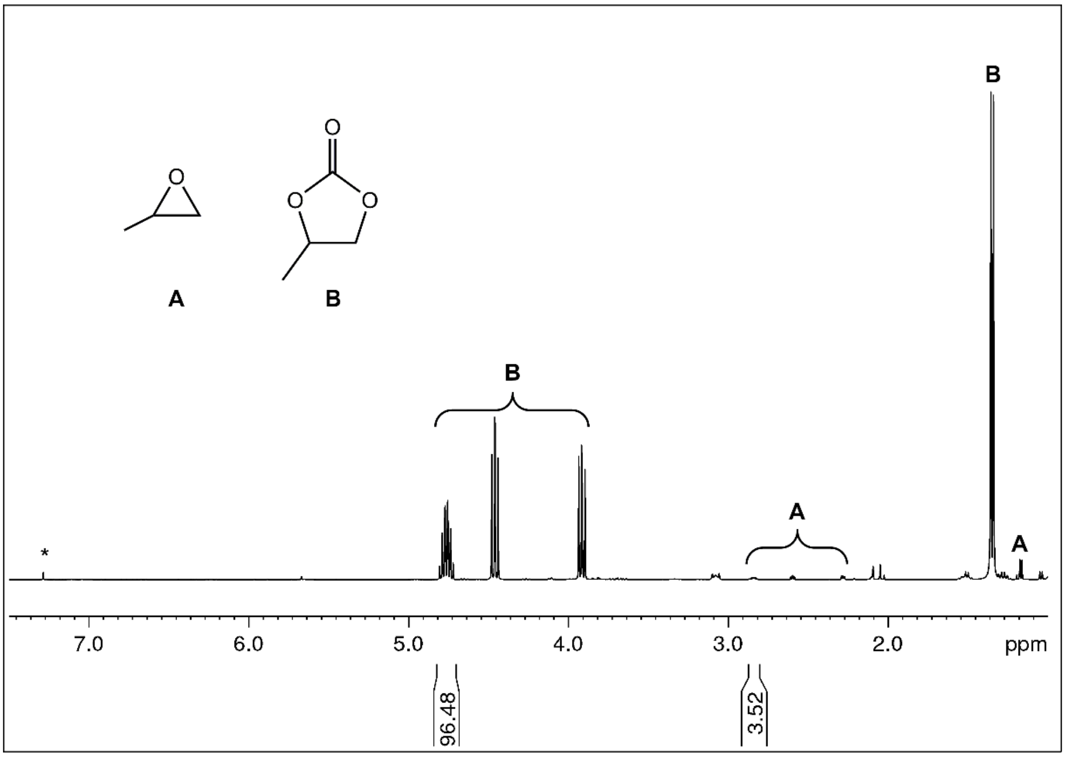

Figure S31. ^{1}H NMR spectrum (26 °C, 400.11 MHz, chloroform-*d*) of the reaction mixture of the catalytic conversion of propylene oxide and CO_2 to propylene carbonate by using 0.5 mol% of Ti(pz^{Me2})$_4$@SBA-15$_{500}$ (**H3-Ti^{+IV}**) as catalyst and TBAI as cocatalyst at 90 °C and 10 bar CO_2.

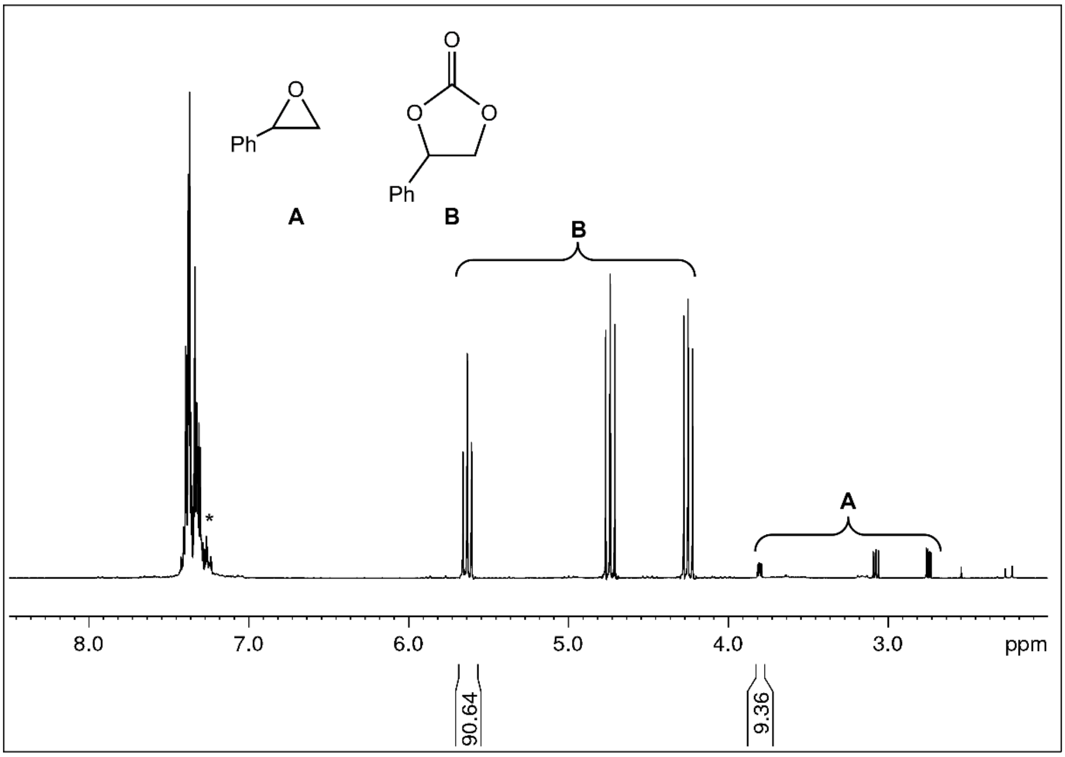

Figure S32. ^{1}H NMR spectrum (26 °C, 400.11 MHz, chloroform-*d*) of the reaction mixture of the catalytic conversion of styrene oxide and CO_2 to styrene carbonate by using 0.5 mol% of Ti(pz^{Me_2})$_4$@SBA-15$_{500}$ (**H3-Ti^{+IV}**) as catalyst and TBAI as cocatalyst at 90 °C and 10 bar CO_2.

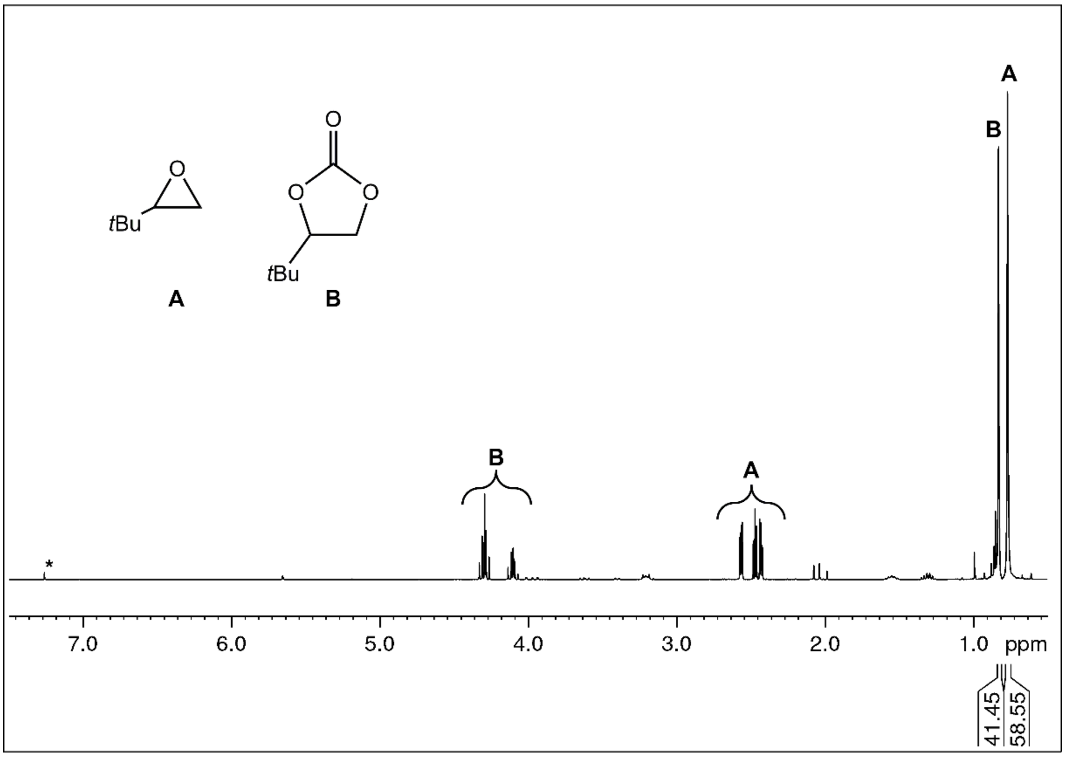

Figure S33. ^{1}H NMR spectrum (26 °C, 400.11 MHz, chloroform-*d*) of the reaction mixture of the catalytic conversion of 2-*tert*-butyloxirane and CO_2 to 3,3-dimethyl-1,2-butene carbonate by using 0.5 mol% of Ti(pz^{Me_2})$_4$@SBA-15$_{500}$ (**H3-Ti^{+IV}**) as catalyst and TBAI as cocatalyst at 90 °C and 10 bar CO_2.

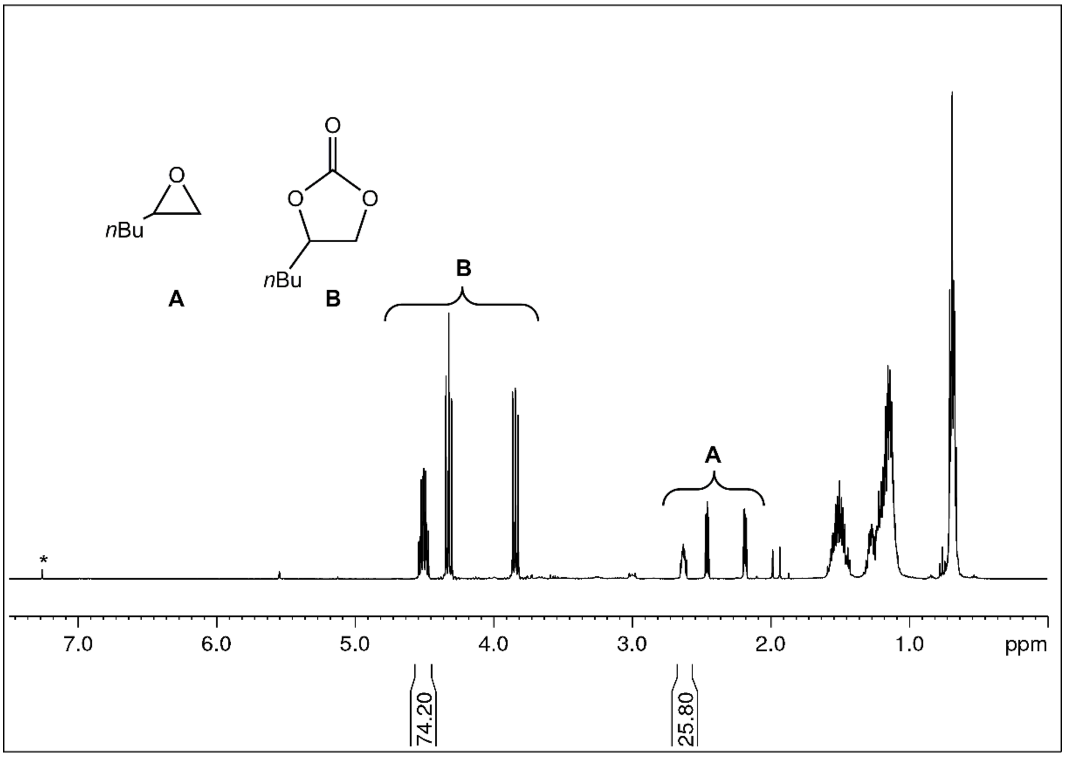

Figure S34. ^{1}H NMR spectrum (26 °C, 400.11 MHz, chloroform-*d*) of the reaction mixture of the catalytic conversion of 1,2-epoxyhexane and CO_2 to 1,2-*n*-hexylene carbonate by using 0.5 mol% of Ti(pz^{Me2})$_4$@SBA-15$_{500}$ (**H3-Ti^{+IV}**) as catalyst and TBAI as cocatalyst at 90 °C and 10 bar CO_2.

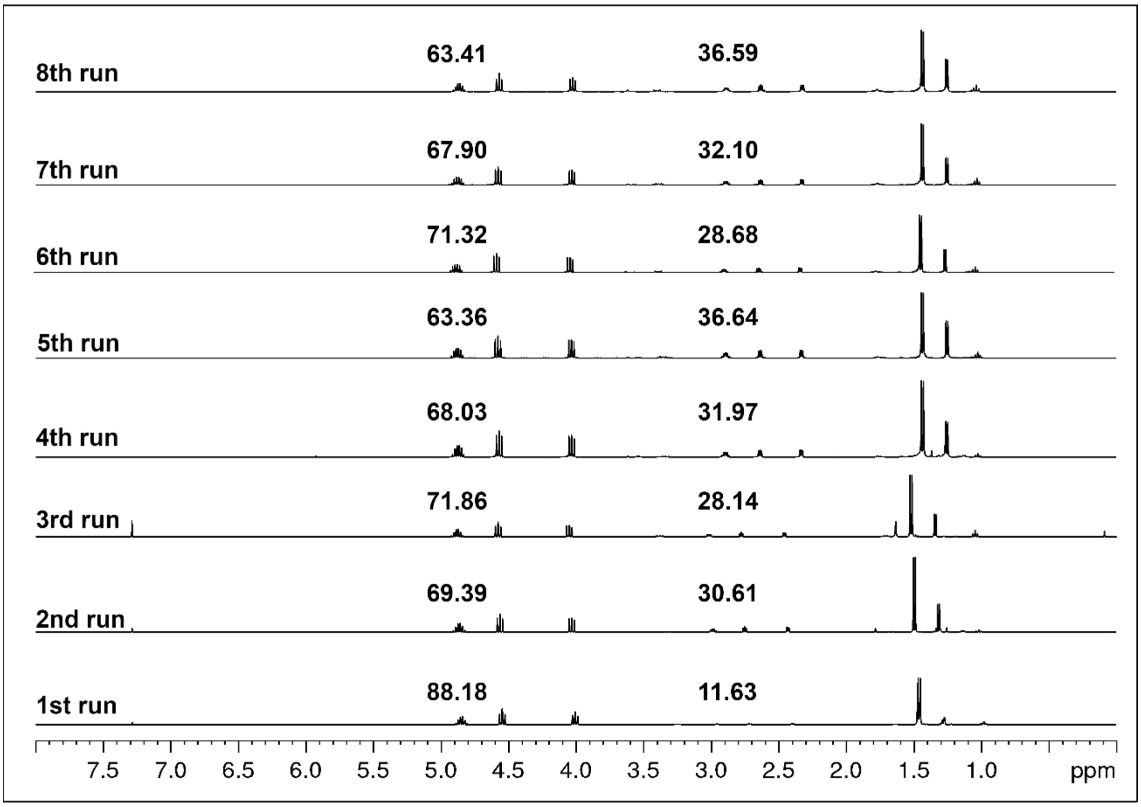

Figure S35. ^{1}H NMR spectra (26 °C, 400.11 MHz, 1–3. run: chloroform-*d*, 4.–8. run: THF-*d*$_8$) of the reaction mixture of the catalytic conversion of propylene oxide and CO_2 to propylene carbonate by using 0.5 mol% of [Mg(pz^{tBu2})$_2$]$_2$@SBA-15$_{500}$ (**H1-Mg**) as catalyst and TBAI as cocatalyst for reuse in eight cycles.

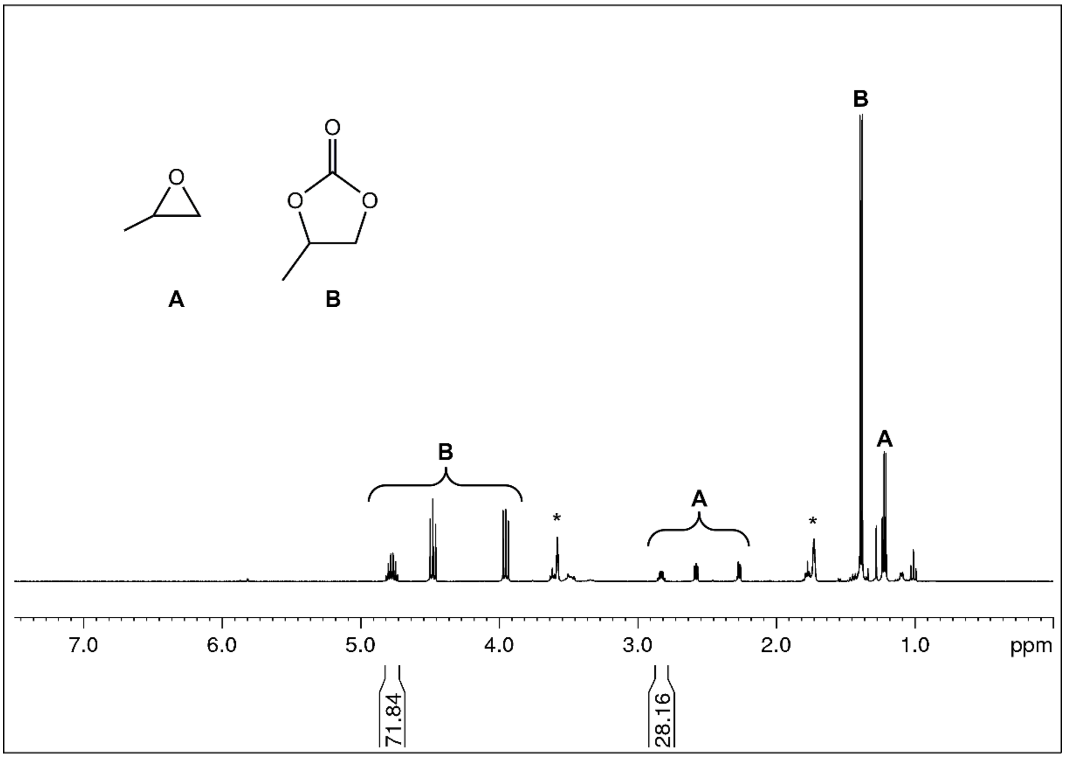

Figure S36. ^{1}H NMR spectrum (26 °C, 400.11 MHz, THF-d_8) of the reaction mixture after 6 h of the catalytic conversion of propylene oxide and CO_2 to propylene carbonate by using 0.5 mol% of [Mg(pz^{tBu_2})$_2$]$_2$@SBA-15$_{500}$ (**H1-Mg**) as catalyst and TBAI as cocatalyst.

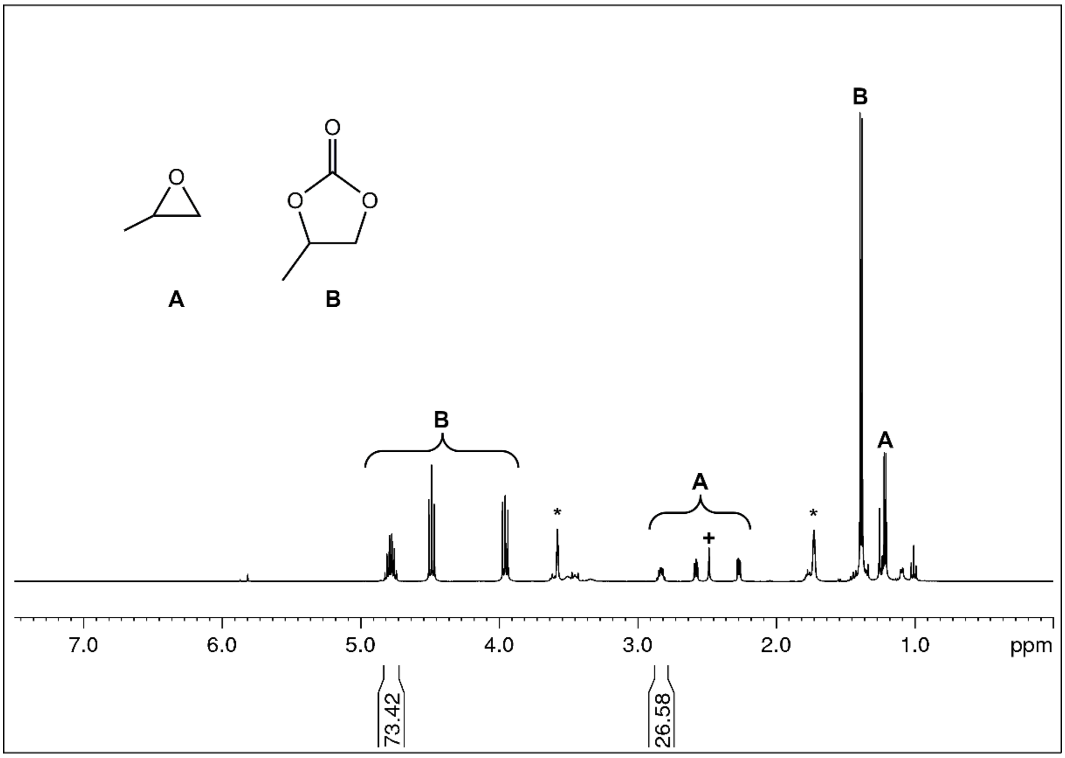

Figure S37. ^{1}H NMR spectrum (26 °C, 400.11 MHz, THF-d_8) of the filtered supernatant solution of the reaction mixture seen in S36 after 18 h.

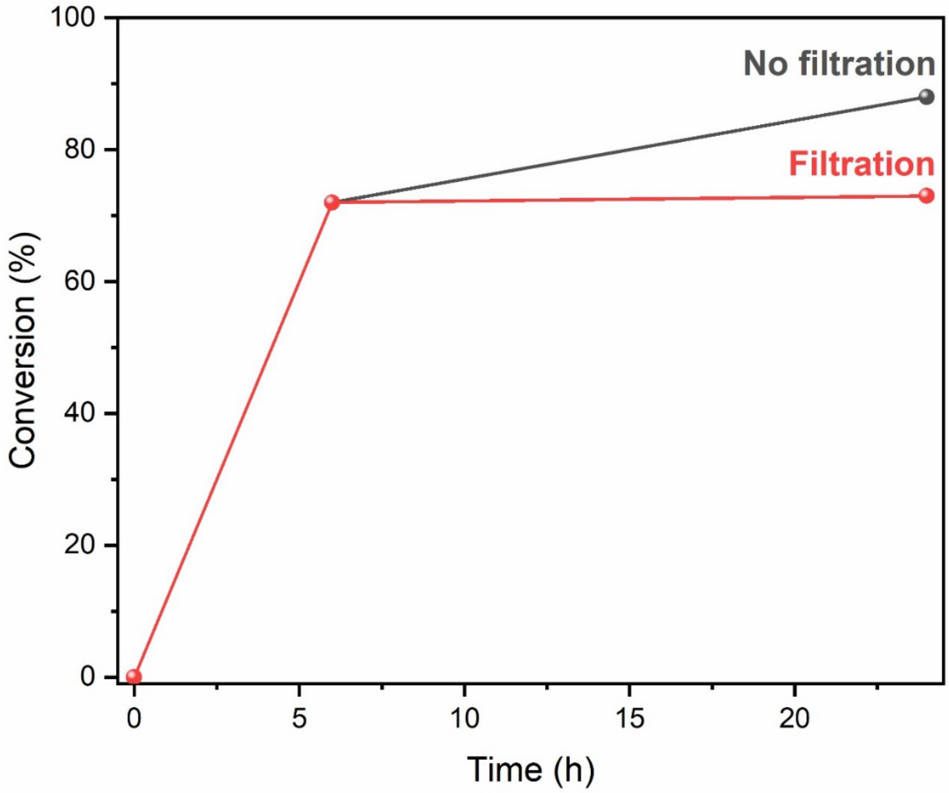

Figure S38. Black: Catalytic conversion of propylene oxide and CO_2 to propylene carbonate by using 0.5 mol% of $[Mg(pz^{tBu_2})_2]_2$@SBA-15$_{500}$ (**H1-Mg**) as catalyst and TBAI as cocatalyst. Red: Catalytic conversion after 6 h under the same conditions and after additional 18 h when **H1-Mg** is filtered off. Determined via ^{1}H NMR (see S25 and S38–39).

Table S1. Leaching test of magnesium when $[Mg(pz^{tBu_2})_2]_2$@SBA-15$_{500}$ (**H1-Mg**) is used as catalyst in the cycloaddition of propylene oxide and CO_2 to propylene carbonate in the span of six catalytic cycles monitored via ICP-OES experiments

**Cycles**	**Mg Leaching [mg]**
1st + 2nd. cycle	0.000000
3rd cycle	0.000000
4th + 5th cycle	0.000504
6th cycle	0.000000

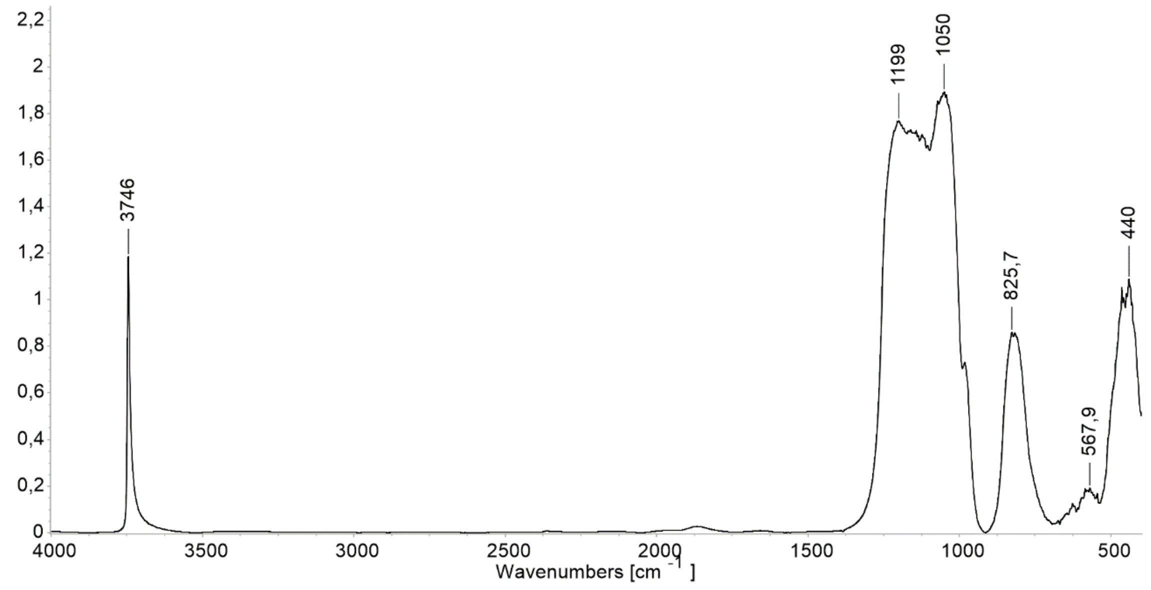

Figure S39. DRIFT spectrum of parent material SBA-15$_{500}$ at 25 °C. Adapted from previous work.[1]

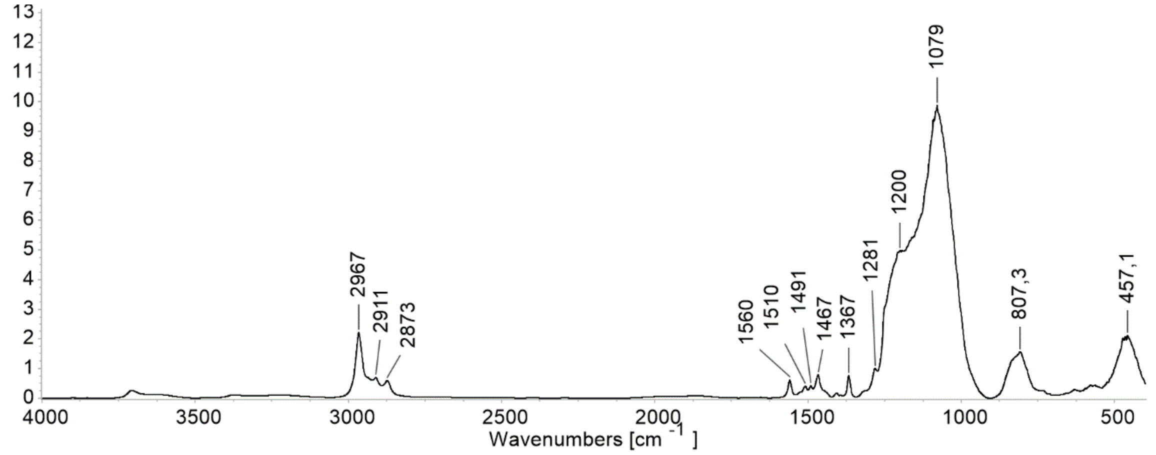

Figure S40. DRIFT spectrum of material [Mg(pz^{tBu_2})$_2$]$_2$@SBA-15$_{500}$ (**H1-Mg**) at 25 °C.

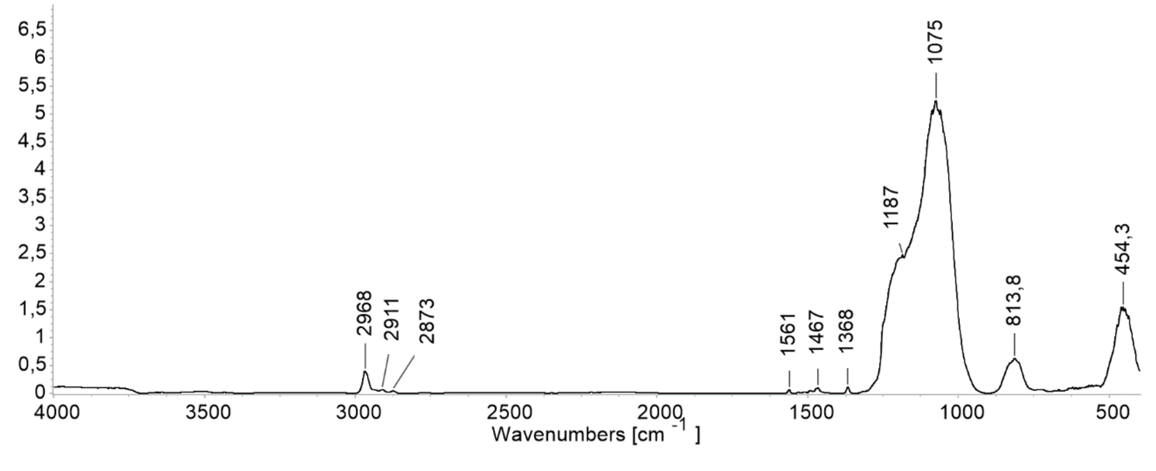

Figure S41. DRIFT spectrum of material Al(pz^{tBu_2})$_3$@SBA-15$_{500}$ (**H2-Al**) at 25 °C.

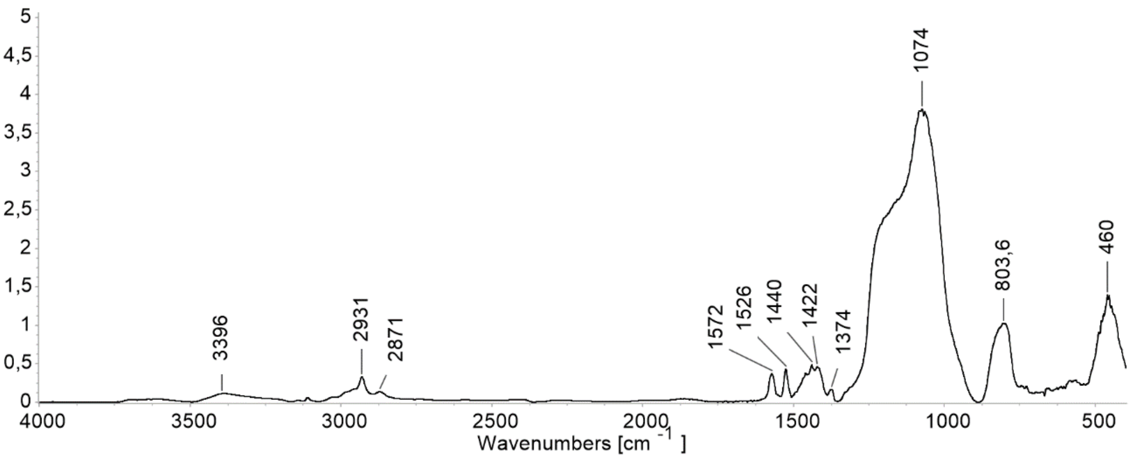

Figure S42. DRIFT spectrum of material $Ti^{+IV}(pz^{Me_2})_4$@SBA-15_{500} (**H3-Ti^{+IV}**) at 25 °C.

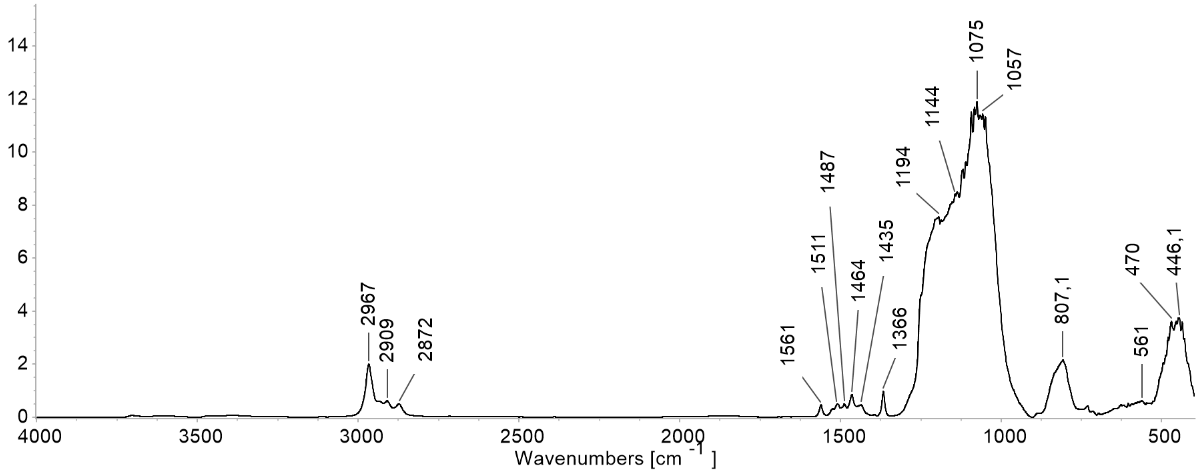

Figure S43. DRIFT spectrum of material $Ti^{+III}(pz^{tBu_2})_4$@SBA-15_{500} (**H4-Ti^{+III}**) at 25 °C.

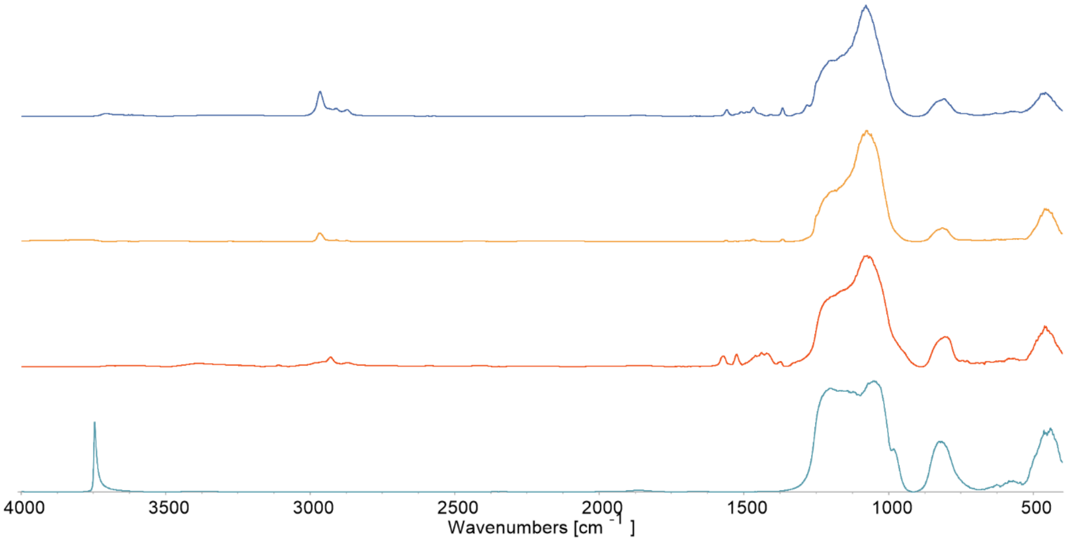

Figure S44. Comparison of DRIFT spectra of parent material SBA-15_{500} (bottom, light blue), $Ti(pz^{Me_2})_4$@SBA-15_{500} (**H3-Ti^{+IV}**, red), $Al(pz^{tBu_2})_3$@SBA-15_{500} (**H2-Al**, yellow) and $[Mg(pz^{tBu_2})_2]_2$@SBA-15_{500} (**H1-Mg**, top, dark blue) at 25 °C.

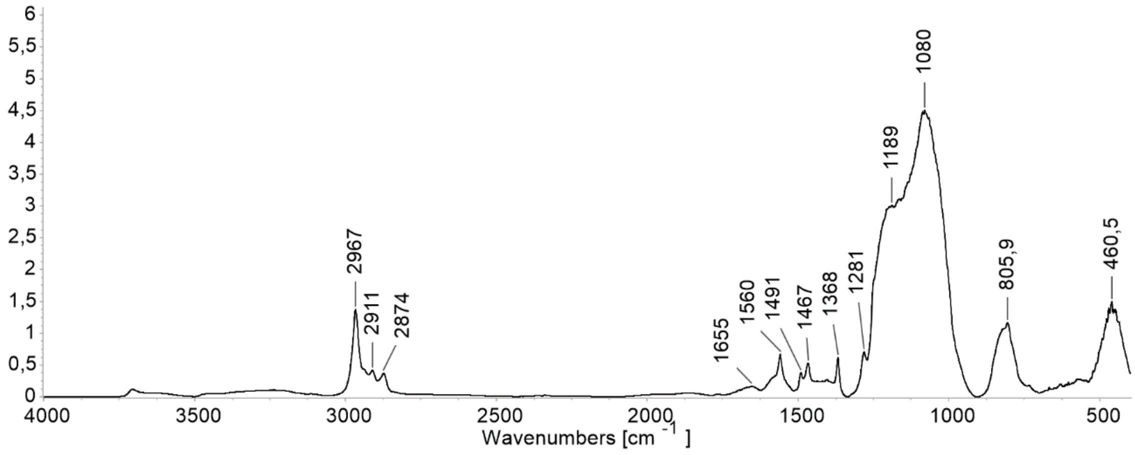

Figure S45. DRIFT spectrum of CO_2@[Mg(pz^{tBu_2})$_2$]$_2$@SBA-15$_{500}$ (**CO_2@H1-Mg**) at 25 °C.

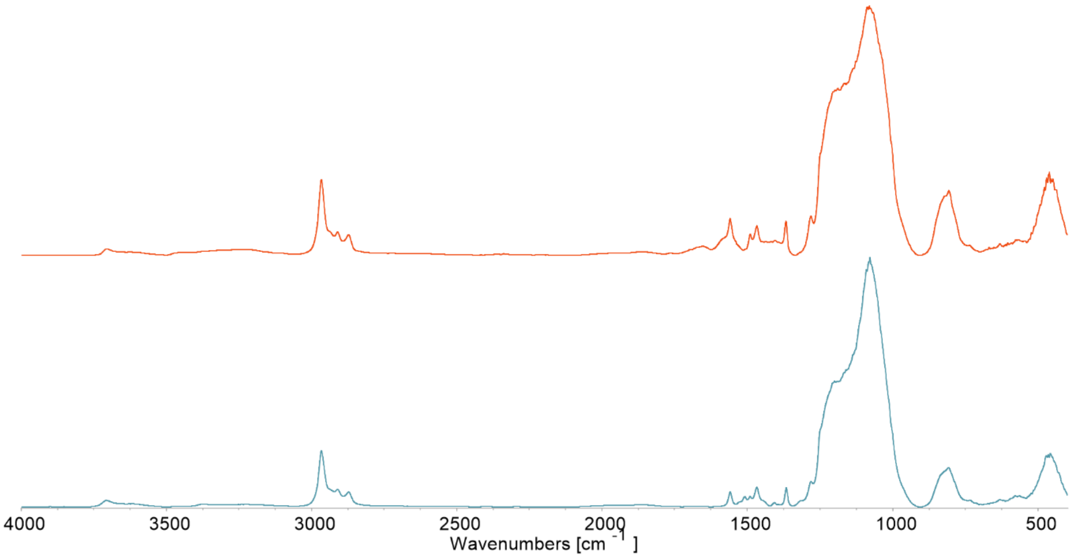

Figure S46. Comparison of DRIFT spectra of [Mg(pz^{tBu_2})$_2$]$_2$@SBA-15$_{500}$ (**H1-Mg**, bottom) and CO_2@[Mg(pz^{tBu_2})$_2$]$_2$@SBA-15$_{500}$ (**CO_2@H1-Mg**, top) at 26°C.

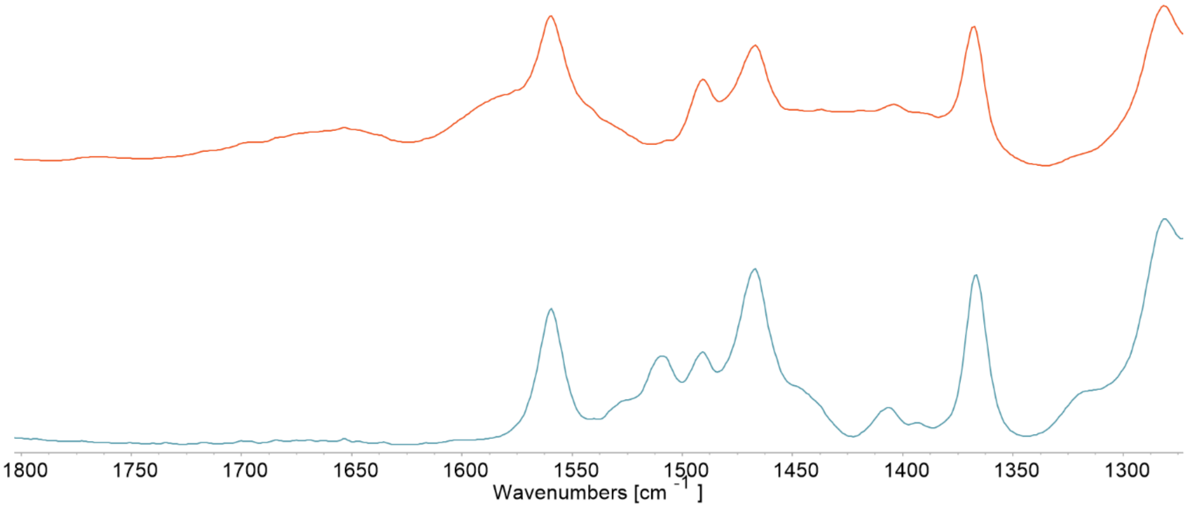

Figure S47. Comparison of zoomed in DRIFT spectra of [Mg(pz^{tBu_2})$_2$]$_2$@SBA-15$_{500}$ (**H1-Mg**, bottom) and CO_2@[Mg(pz^{tBu_2})$_2$]$_2$@SBA-15$_{500}$ (**CO_2@H1-Mg**, top) at 26°C.

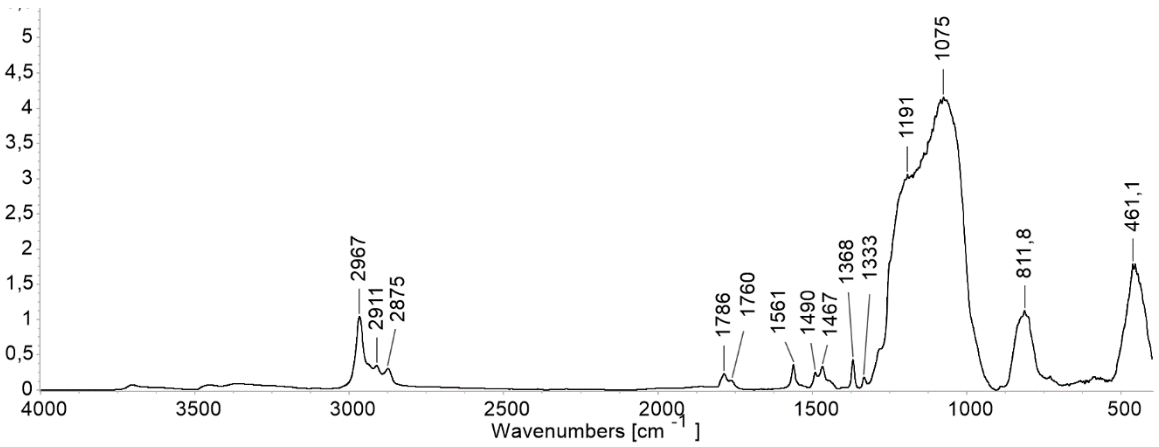

Figure S48. DRIFT spectrum of CO_2@Al(pz^{tBu_2})$_3$@SBA-15$_{500}$ (**CO_2@H2-Al**) at 25 °C.

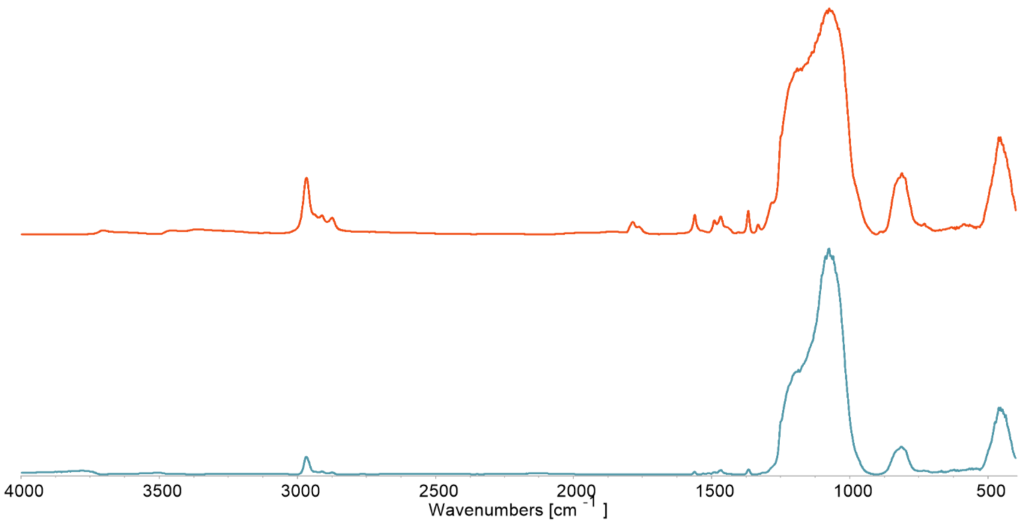

Figure S49. Comparison of DRIFT spectra of Al(pz^{tBu_2})$_3$@SBA-15$_{500}$ (**H2-Al**, bottom) and CO_2@Al(pz^{tBu_2})$_3$@SBA-15$_{500}$ (**CO_2@H2-Al**, top) at 26°C.

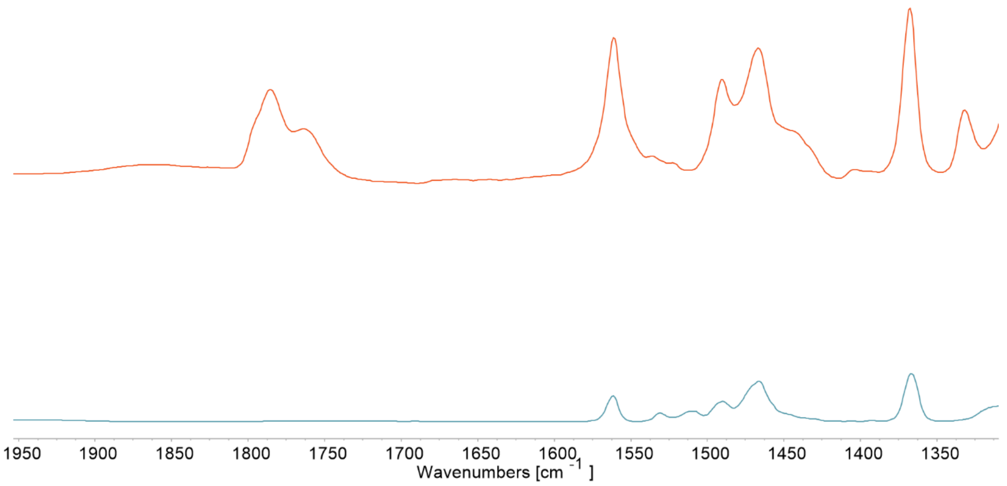

Figure S50. Comparison of zoomed in DRIFT spectra of Al(pz^{tBu_2})$_3$@SBA-15$_{500}$ (**H2-Al**, bottom) and CO_2@Al(pz^{tBu_2})$_3$@SBA-15$_{500}$ (**CO_2@H2-Al**, top) at 26°C.

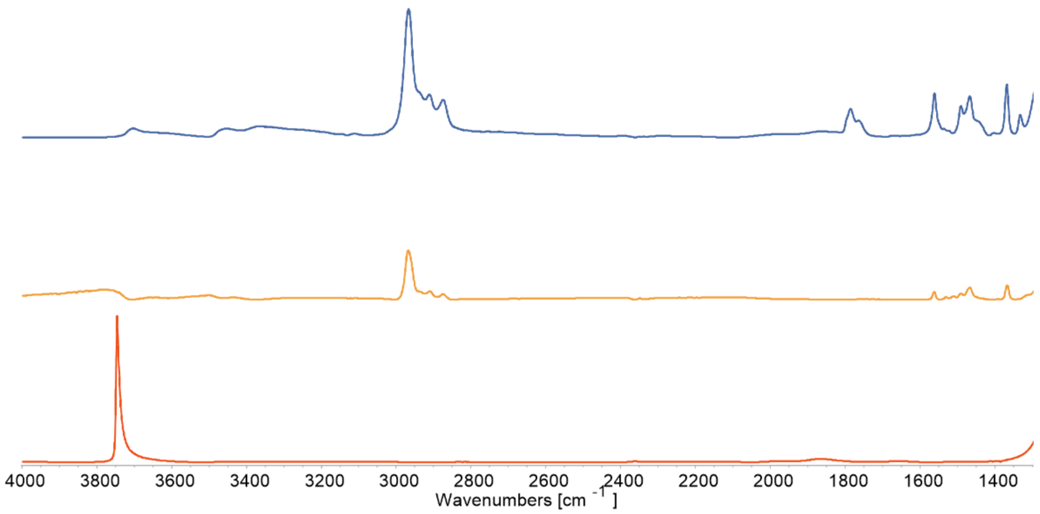

Figure S51. Comparison of DRIFT spectra of parent material SBA-15$_{500}$ (bottom, red), Al(pz^{tBu2})$_3$@SBA-15$_{500}$ (**H2-Al**, middle, yellow), CO_2@Al(pz^{tBu2})$_3$@SBA-15$_{500}$ (**CO_2@H2-Al**, top, blue) at 26 °C.

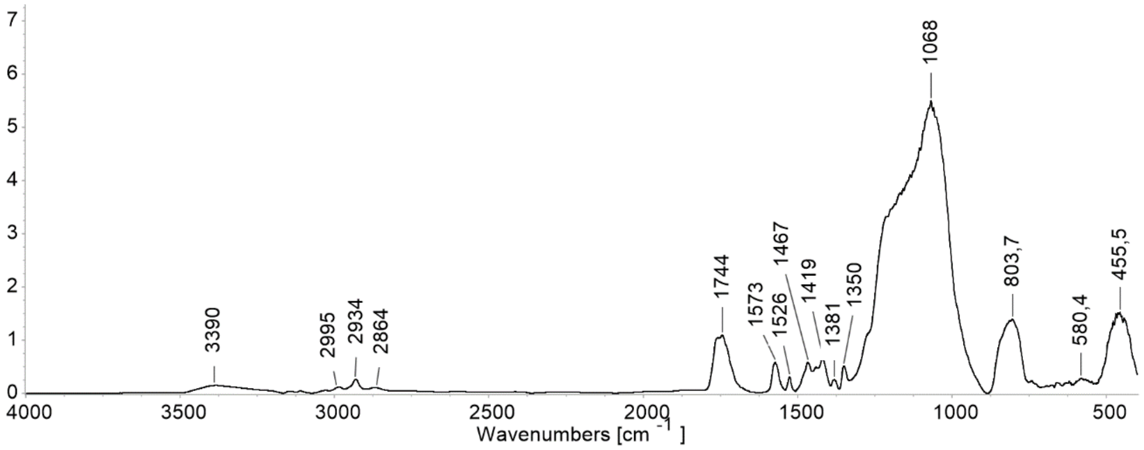

Figure S52. DRIFT spectrum of CO_2@Ti(pz^{Me2})$_4$@SBA-15$_{500}$ (**CO_2@H3-Ti^{+IV}**) at 25 °C.

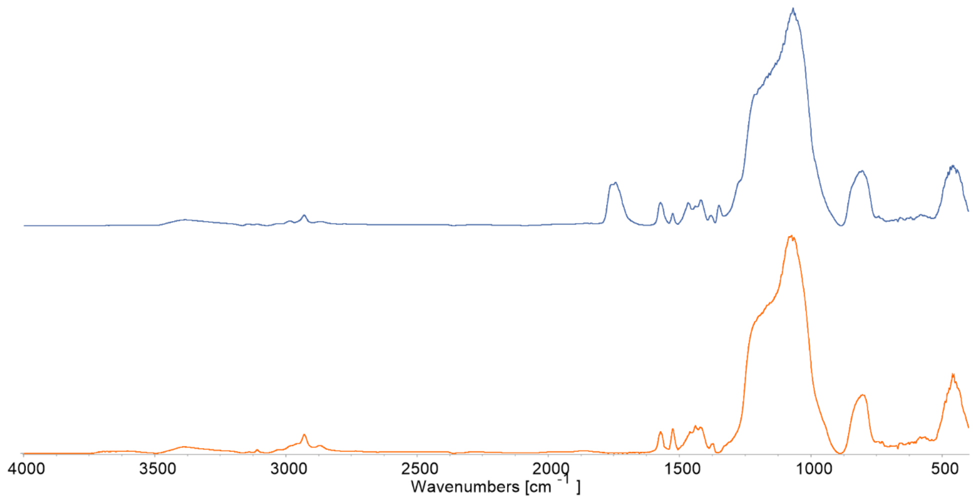

Figure S53. Comparison of DRIFT spectra of Ti(pz^{Me2})$_4$@SBA-15$_{500}$ (**H3-Ti^{+IV}**, bottom) and CO_2@Ti(pz^{Me2})$_4$@SBA-15$_{500}$ (**CO_2@H3-Ti^{+IV}**, top) at 26°C.

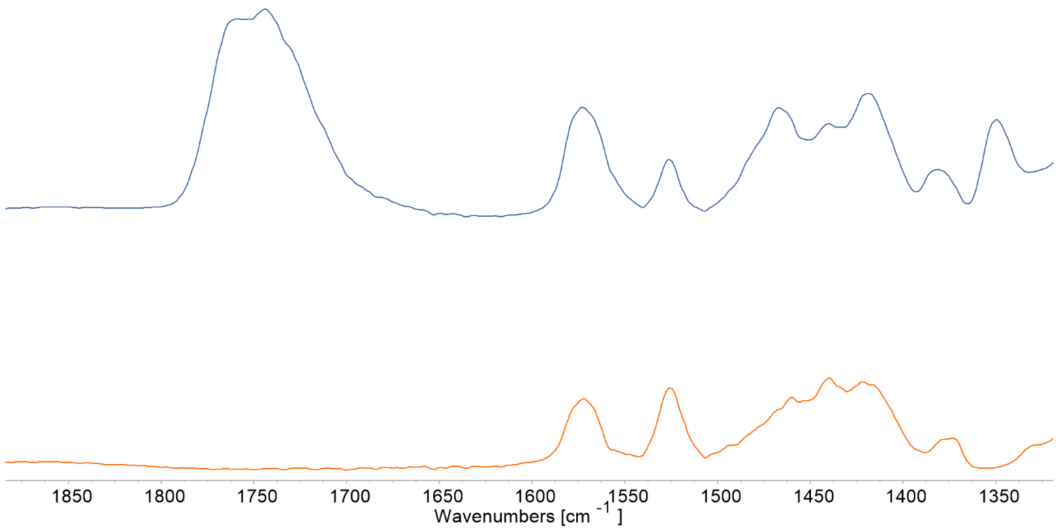

Figure S54. Comparison of zoomed in DRIFT spectra of Ti(pz^{Me2})$_4$@SBA-15$_{500}$ (**H3-Ti^{+IV}**, bottom) and CO_2@Ti(pz^{Me2})$_4$@SBA-15$_{500}$ (**CO_2@H3-Ti^{+IV}**, top) at 26°C.

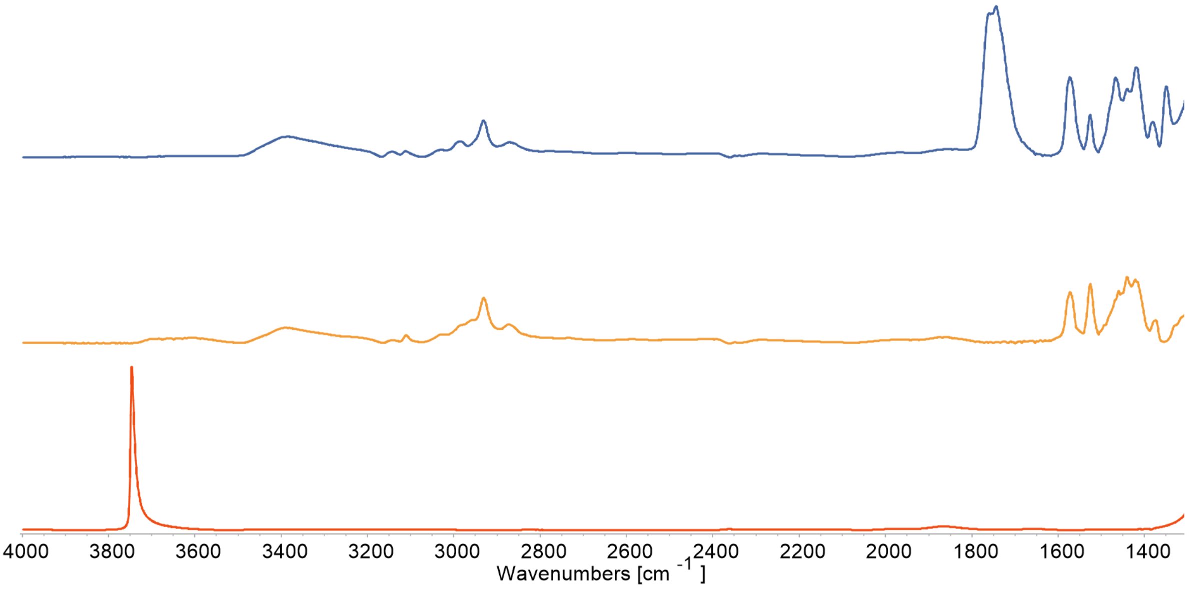

Figure S55. Comparison of DRIFT spectra of parent material SBA-15$_{500}$ (bottom, red), Ti(pz^{Me2})$_4$@SBA-15$_{500}$ (**H3-Ti^{+IV}**, middle, yellow), CO_2@Ti(pz^{Me2})$_4$@SBA-15$_{500}$ (**CO_2@H3-Ti^{+IV}**, top, blue) at 26 °C.

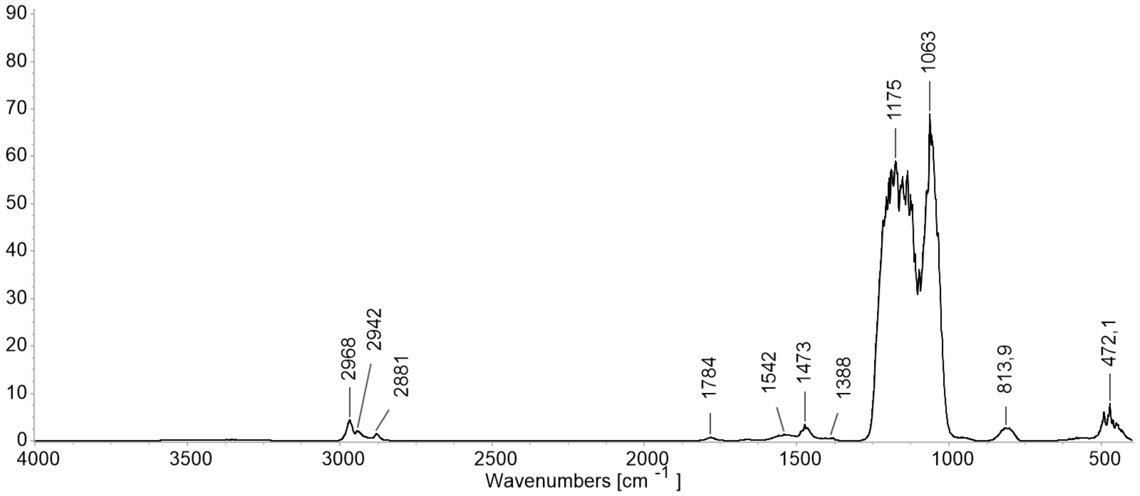

Figure S56. DRIFT spectrum of material [Mg(pz^{tBu_2})$_2$]$_2$@SBA-15$_{500}$ (**H1-Mg**) after eight consecutive cycles of the catalytic conversion of proyplene oxide and CO_2 to propylene carbonate measured at 25 °C.

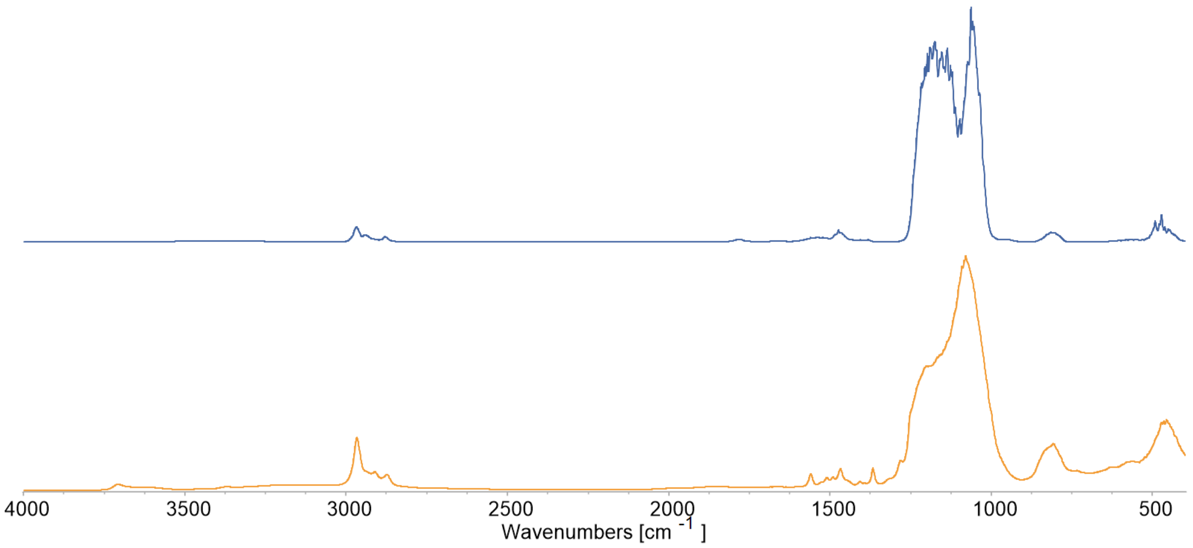

Figure S57. Comparison of DRIFT spectra at 26 °C of [Mg(pz^{tBu_2})$_2$]$_2$@SBA-15$_{500}$ (**H1-Mg**) before the catalytic conversion (bottom) and after eight catalytic cycles (top) of the cycloaddition of CO_2 and propylene oxide.

Crystal Structure and Crystallographic Data

Crystals of **M1-Ti^{+IV}** suitable for X-Ray crystallography were grown by standard techniques from solutions using toluene at –40°C. For X-ray crystallography, crystals were handpicked in a glovebox, coated with Parabar 10312 and stored on microscopic slides and fixed on a microloop. Data collection was carried out on a Bruker APEX II Duo diffractometer using QUAZAR optics for Mo K_α (λ = 0.71073 Å). The data collection strategy was determined using COSMO with ω scans.[2] Raw data were processed by APEX[3] and SAINT,[4] corrections for absorption effects were applied using SADABS.[5] The structures were solved by direct methods and refined against all data by full-matrix least-squares methods on F^2 using SHELXTL[6] and Shelxle.[7] Plots were generated by using MERCURY[8] and POV-Ray.[9]

Only a connectivity could be given for this measurement. The crystals were of poor quality. Additional reflections can be observed in the reciprocal space, attempts to assign them to a second individual and refining them as a twin did not yield in better results. Restraints (RIGU, SIMU, SADI) due to large thermal motion of the molecule. This could not be resolved by a disorder model. Distances and angles are not reliable.

Space group: $P2_1$

Unit Cell:	a = 25.75(2)	
	b = 19.064(18)	β = 117.775(11)
	c = 26.49(2)	

CCDC: 2505638

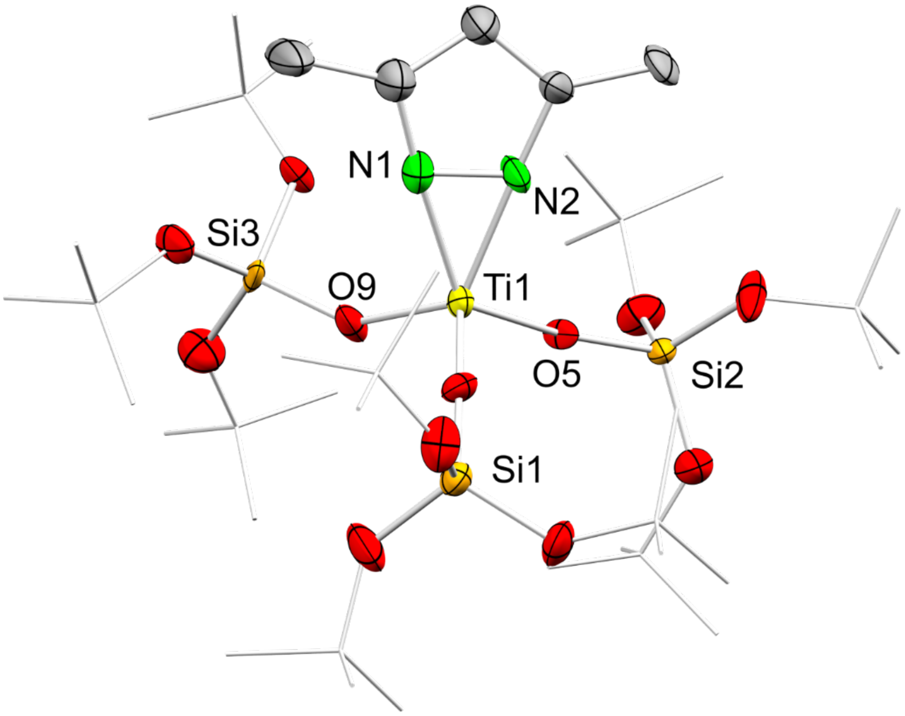

Figure S58. Connectivity of Ti^{+IV}(pz^{Me2})[OSi(O*t*Bu)$_3$]$_3$ (**M1-Ti^{+IV}**). Thermal ellipsoids set at 50% probability. Hydrogen atoms are omitted and *t*Bu moieties are set as wireframe for clarity.

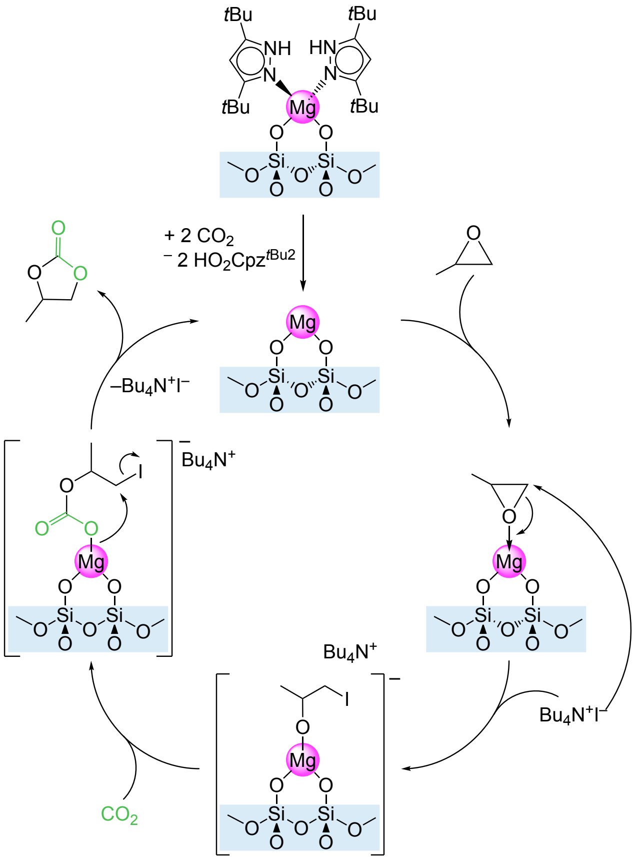

Figure S59. Proposed catalytic scenario for the conversion of CO_2 and epoxides to cyclic carbonates at the surface species [(≡SiO)$_2$Mg(Hpz^{tBu2})$_2$] of material **H1-Mg**.

(1) Poolwong, J.; Kracht, F.; Moinet, E.; Liang, Y.; D'Elia, V.; Anwander, R. Samarium- and Ytterbium-Grafted Periodic Mesoporous Silica for Carbon Dioxide Capture and Conversion. *Inorg. Chem.* **2023**, *62* (43), 17972–17984. https://doi.org/10.1021/acs.inorgchem.3c02995.

(2) *COSMO v. 1.61*, Bruker AXS Inc., Madison, WI, **2012**.

(3) *APEX 3 V. 2017.3-0*, Bruker AXS Inc., Madison, WI, **2016**.

(4) *SAINT V. 8.40B*, Bruker Nano, Inc., Madison, WI, **2019**.

(5) *SADABS* Krause, L.; Herbst-Irmer, R.; Sheldrick, G. M. & Stalke, D. *J. Appl. Cryst.* **2015**, *48*, 3–10.

(6) *SHELXTL*, Sheldrick G. M. Integrated space-group and crystal structure determination. *Acta Cryst.* **2015**, *A71*, 3–8.

(7) *SHELXLE*, C. B. Hübschle, G. M. Sheldrick, B. Dittrich, ShelXle: A Qt graphical user interface for SHELXL. *J. Appl. Crystallogr.* **2011**, *44*, 1281–1284.

(8) Mercury: Visualization and analysis of crystal structure. C. F. Macrae, P. R. Edgington, P. McCabe, E. Pidcock, G. P. Shields, R. Taylor, M. Towler and J. van de Streek, J. *Appl. Cryst.,* **2006**, *39*, 453–457.

(9) *POV-Ray v. 3.6*, Persistence of Vision Pty. Ltd., Williamstown, Victoria, Australia, **2004**. http://www.povray.org/
